# Intranasal delivery of imaging agents to the brain

**DOI:** 10.7150/thno.98473

**Published:** 2024-08-19

**Authors:** Abdallah Almahmoud, Harendra S Parekh, Brett M Paterson, Karnaker Reddy Tupally, Viktor Vegh

**Affiliations:** 1 Centre for Advanced Imaging, Australian Institute for Bioengineering and Nanotechnology, The University of Queensland, Brisbane, QLD, Australia.; 2 School of Pharmacy, The University of Queensland, Brisbane, QLD, Australia.; 3 Department of Allied Medical Sciences, Faculty of Applied Medical Sciences, Jordan University of Science and Technology, Irbid, Jordan.; 4 ARC Training Centre for Innovation in Biomedical Imaging Technology, Brisbane, QLD, Australia.

**Keywords:** intranasal administration, neuroimaging agents, brain imaging, imaging modalities, nose-to-brain

## Abstract

The potential of intranasal administered imaging agents to altogether bypass the blood-brain barrier offers a promising non-invasive approach for delivery directly to the brain. This review provides a comprehensive analysis of the advancements and challenges of delivering neuroimaging agents to the brain by way of the intranasal route, focusing on the various imaging modalities and their applications in central nervous system diagnostics and therapeutics. The various imaging modalities provide distinct insights into the pharmacokinetics, biodistribution, and specific interactions of imaging agents within the brain, facilitated by the use of tailored tracers and contrast agents.

**Methods:** A comprehensive literature search spanned PubMed, Scopus, Embase, and Web of Science, covering publications from 1989 to 2024 inclusive. Starting with advancements in tracer development, we going to explore the rationale for integration of imaging techniques, and the critical role novel formulations such as nanoparticles, nano- and micro-emulsions in enhancing imaging agent delivery and visualisation.

**Results:** The review highlights the use of innovative formulations in improving intranasal administration of neuroimaging agents, showcasing their ability to navigate the complex anatomical and physiological barriers of the nose-to-brain pathway. Various imaging techniques, MRI, PET, SPECT, CT, FUS and OI, were evaluated for their effectiveness in tracking these agents. The findings indicate significant improvements in brain targeting efficiency, rapid uptake, and sustained brain presence using innovative formulations.

**Conclusion:** Future directions involve the development of optimised tracers tailored for intranasal administration, the potential of multimodal imaging approaches, and the implications of these advancements for diagnosing and treating neurological disorders.

## 1. Introduction

**Figure 1:**
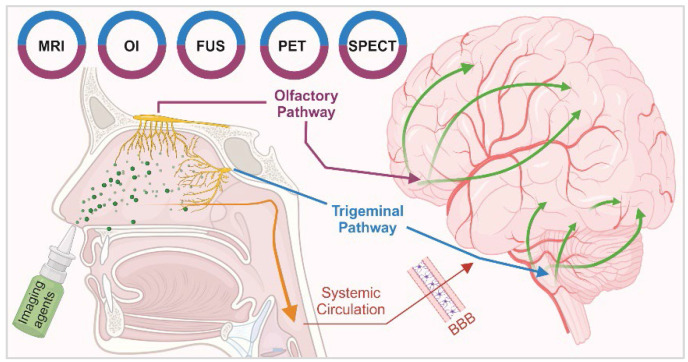
Pathways of intranasal drug delivery and associated imaging modalities: This illustration depicts the IN administration of imaging agents through the nasal cavity and their subsequent pathways to the brain. It highlights two primary routes: the olfactory (purple) and trigeminal (blue) pathways and the secondary systemic circulation route across the BBB. Various imaging techniques are employed to trace these agents: MRI, OI, FUS, SPECT, and PET are color-coded to represent the respective IN route that each imaging technique monitors. [created with BioRender.com].

The intranasal (IN) administration route for delivering neuroimaging agents has emerged as a promising alternative to traditional approaches of systemic administration. This method capitalises on the unique anatomical connections between distinct regions of the nasal cavity and the central nervous system (CNS), allowing for a direct, rapid and potentially sustained pathway from nose-to-brain 1. IN administration bypasses the blood-brain barrier (BBB) and reduces systemic exposure, potentially minimising side effects while maximising the brain bioavailability of imaging agents. This is particularly beneficial in the context of CNS disorders, as features inherent to the BBB prevent or significantly hamper the delivery of agents, therapeutic and diagnostic, intended for brain delivery 2, 3. Figure 1 illustrates the IN administration of imaging agents through the nasal cavity and their subsequent pathways to the brain, highlighting the primary routes: the olfactory (purple) and trigeminal (blue) pathways and the secondary systemic circulation route across the BBB. Various imaging techniques are employed to trace these agents: magnetic resonance imaging (MRI), optical imaging (OI), focused ultrasound (FUS), single photon emission computed tomography (SPECT), and positron emission tomography (PET) are colour-coded to represent the respective IN route that each imaging technique monitors.

IN drug delivery (INDD) while offering numerous benefits, also presents challenges needing to be addressed for optimal effectiveness, including

i) mucociliary clearance in the respiratory region, which rapidly removes foreign substances limiting drug residence time and absorption;

ii) enzymatic degradation by nasal mucosa enzymes, particularly affecting peptides and proteins; poor permeability of large, hydrophilic molecules through the nasal mucosa;

iii) potential formulation-induced irritation and toxicity, impacting patient compliance and safety; and

iv) variable, unpredictable absorption due to individual differences in nasal anatomy and physiology.

To overcome these challenges, various formulation strategies can be employed, such as

i) using mucoadhesive polymers to prolong drug residence time;

ii) adding enzyme inhibitors to protect drugs from degradation;

iii) incorporating permeation enhancers such as *in situ* gel systems, cyclodextrins, and polymers to facilitate larger molecule absorption;

iv) encapsulating drugs in nanoparticles (NPs) or liposomes to protect them and enhance absorption;

v) developing controlled release systems to maintain therapeutic drug levels over time; and

vi) optimising the pH and osmolarity of formulations to minimise irritation and enhance patient compliance.

Additionally, innovative formulations like nano-emulsions (NEs) and micro-emulsions (MEs) can be used to further enhance delivery efficiency and diagnostic precision. By addressing these challenges with innovative formulation strategies the effectiveness of INDD can be significantly improved 4-6. Furthermore, advanced delivery devices designed for precise/preferential targeting (e.g. to olfactory region) can improve the distribution and absorption of imaging agents within the nasal cavity, and into the brain. Devices that facilitate the delivery of formulations to the olfactory cleft region of the nasal cavity would minimise loss to swallowing or lung deposition, enhancing the efficiency of nose-to-brain delivery 6.

Medical imaging techniques used in evaluating IN administration of imaging agents, such as MRI, PET, SPECT, OI, gamma scintigraphy, autoradiography, and certain types of computed tomography (CT) scans, have revolutionised our ability to visualise and map brain structures and functions *in vivo* 7, 8. In contrast, other diagnostic imaging procedures like conventional X-ray images, CT, and US mainly offer images of physical form and often rely on intravenous (IV) administration of imaging agents, which can present challenges in terms of bioavailability due to insufficient concentrations crossing the intact BBB, and potential systemic side effects from accumulation in the periphery. Conversely, when the BBB is compromised, such as in brain cancer, these imaging agents can take advantage of the altered barrier function 9. These various imaging modalities span the spectrum of structural to functional imaging. The choice of imaging modality and the delivery method of agents, such as via IN administration, can significantly affect both the type and quality of the images obtained, as well as the insights gleaned from these images 10. Therefore, it is critical to understand the differences between these modalities when designing studies and interpreting results that involve IN administration of imaging agents.

Despite these advantages, the field of IN administration of neuroimaging agents to the brain remains nascent, with a growing but still relatively limited body of literature and evidence. This systematic review aims to synthesise current knowledge on the topic, evaluate the effectiveness of this route based on available evidence, and identify directions for future research. In doing so, we strive to contribute to the understanding and development of this promising approach in the field of neuroimaging.

## 2. Imaging of intranasal delivery

The speed and sensitivity of PET imaging make it an invaluable tool for real-time tracking of metabolic processes and physiological activities within the body. Although the potential toxicity is associated with long-lived positron-emitting radionuclides (half-life > 2 h), these substances can pose safety concerns due to prolonged radiation exposure. SPECT and gamma scintigraphy provide complementary insights to PET, and the methods are particularly useful in visualising the physical transit of radioisotopes and their distribution in the brain 11. OI offers a distinct advantage in visualising biological processes at the cellular level 12. MRI stands out for its non-invasive nature and high-resolution structural imaging capability. Here, the use of contrast agents provides opportunities for assessing drug movement and BBB interaction 13, 14. Although less sensitive to soft tissue contrast, CT gains significant value when combined with PET or SPECT as it provides precise anatomical details with functional insights, leading to more accurate disease identification 15, 16. Gold NPs (AuNPs) as a source of X-ray attenuation provide a mechanism by which CT images can be used as a real-time tracking device 16.

**Figure 2:**
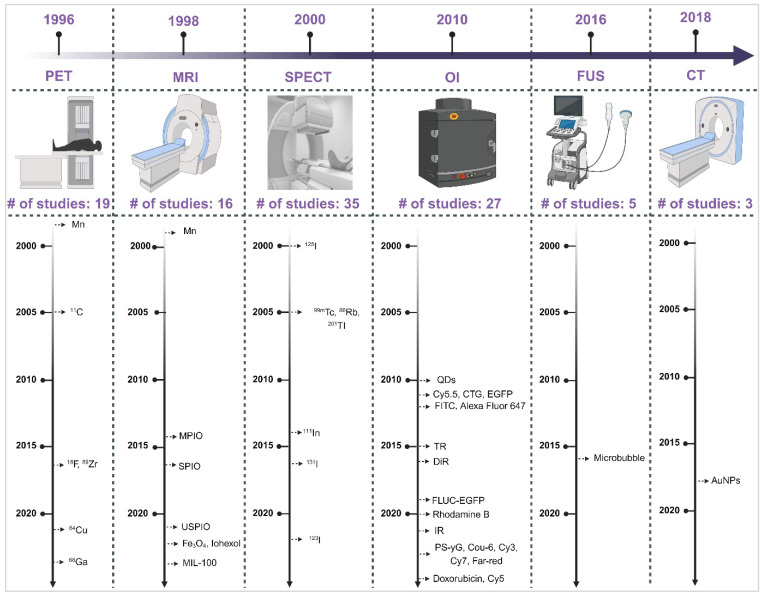
Timeline of development for imaging modalities and neuroimaging agents in INDD. [created with BioRender.com].

Understanding the strengths and weaknesses of each imaging modality is crucial for optimising their use in the INDD pathway. Table 1 presents a comprehensive comparison of the pros and cons of various imaging modalities and neuroimaging agents. The table highlights plausible applications for each modality based on their unique characteristics, providing a clear guide for researchers and clinicians in selecting the appropriate imaging technique for their specific needs. Moreover, the timeline provided in Figure 2 illustrates the progression and number of studies involving various imaging modalities (SPECT, PET, OI, MRI, FUS, CT) for IN neuroimaging agents from 1996 to 2024. The timeline highlights the emergence and adoption of different neuroimaging agents specific to each modality, providing a systematic understanding of the technological advancements in this research area.

Building on this foundational understanding, the subsequent sections will provide an in-depth analysis of each imaging modality and their respective agents in nose-to-brain imaging.

### 2.1. Single Photon Emission Computed Tomography and Gamma Scintigraphy

Gamma scintigraphy and SPECT are both nuclear medicine imaging techniques that use gamma-emitting rays to visualise internal physiological processes. Gamma scintigraphy provides 2D images and is primarily used for simpler diagnostic tasks such as evaluating organ function and detecting infections. In contrast, SPECT offers 3D imaging by rotating around the object to capture multiple angles, enabling more detailed examinations 17. Gamma scintigraphy enables precise tracking and visualisation of the radiotracer transit and distribution within brain tissue. It has been applied for evaluating the INDD. The radiotracers are designed to travel along the olfactory and trigeminal pathways to the brain.

A range of imaging agents for INDD have been investigated using SPECT, each with unique properties and half-lives catering to specific study requirements. Technetium-99m [^99m^Tc] is the predominant radionuclide utilised for SPECT, valued for its widespread availability, favourable photon energy for imaging and short half-life of approximately 6 h 18. Indium-111 [^111^In], with a longer half-life of 2.8 days, is ideal for extended studies and is often used with peptides and antibodies for targeted imaging 19. Iodine-123 [^123^I] offers a balanced half-life of 13.2 h, enabling specific molecule and peptide labelling 20. Whereas the longer-lived iodine isotopes (i.e. [^125^I] and [^131^I]) have specialised applications in research and therapy. [^125^I], a low-energy radioactive isotope with a 60 days half-life, is employed in both medical treatments, such as brachytherapy for cancer and research for labelling biological molecules. [^131^I], with a half-life of approximately 8 days, is used extensively in the treatment of thyroid cancer and hyperthyroidism, as well as in diagnostic imaging 21. Thallium-201 [^201^TI], with half-lives of 73 h, serves in specific domains such as radioimmunotherapy and cardiac imaging 22. Lastly, Rubidium-86 [^86^Rb], with a half-life of approximately 18.6 days, is utilised for its positron emission properties, primarily in cardiac studies 23. Together, these agents provide a comprehensive toolkit, ensuring optimal imaging and facilitating advancements in INDD.

#### 2.1.1. Technetium-99m ([^99m^Tc])

In 2005, Vyas *et al.* assessed nose-to-brain delivery of [^99m^Tc]-labelled zolmitriptan MEs and zolmitriptan mucoadhesive MEs in rats 24. The aim was to explore the efficacy of these formulations in delivering zolmitriptan directly to the brain through nose-to-brain, particularly for treating acute migraine attacks. Radiolabelling zolmitriptan with [^99m^Tc] was critical for tracking the *in vivo* distribution of the drug, enabling the quantification of drug accumulation in the brain and other organs following IN administration and IV injection. Measurements of drug concentrations in blood and brain at predetermined intervals, along with calculations of drug targeting efficiency percentage (DTE%), as described by Behl *et al.* in 1998, assess the average ratio of drug delivery between IN administration and IV injection over time 25. The nose-to-brain drug direct transport percentage (DTP%) measures how much of the drug reaches the brain directly from the nasal route, compared to the total drug amount reaching the brain from the same route. The study revealed that the IN administration of zolmitriptan, particularly via mucoadhesive MEs, facilitated a rapid uptake of the drug into the brain with a DTE% of 533 and a DTP% of 81, surpassing other formulations, including zolmitriptan MEs a DTE% of 255 and a DTP% of 43 and zolmitriptan solutions a DTE% of 189 and a DTP% of 47. The rapid onset of drug action observed, characterised by a relatively short T_max_ (time to reach maximum concentration (C_max_) in the brain), highlights the direct transport efficiency of the mucoadhesive ME formulation via the IN administration. The tracer's retention time in the brain was monitored up to 8 h after administration, with zolmitriptan mucoadhesive MEs showing brain retention of 0.31 % injected dose per gram (ID/g) at 8 h. The formulations were administered in the nostrils using a micropipette. The administered volume of the drug formulation intranasally was 10 µL in each nostril. The quantification of the drug that actually reached the brain after IN administration of zolmitriptan mucoadhesive MEs was measured as a brain uptake ratio at 0.50 h post-administration, with values of ~1% ID/g. The gamma scintigraphy imaging provided a visual confirmation of this rapid uptake, showcasing a marked accumulation of the drug in the brain's olfactory region, which serves as a gateway for the nose-to-brain transport, indicating that the drug likely leverages olfactory neural pathways and systemic pathway for entry into the brain. The authors reported significantly higher activity in the brain following IN administration compared to IV injection for up to 8 h. Clearance is a critical aspect of drug delivery and pharmacokinetics, because it determines the duration of drug efficacy and potential side effects. Given the rapid uptake into the brain via the nose-to-brain route, it would be expected that the clearance mechanisms also play a significant role in mediating the overall drug residence time within brain parenchyma. Typically, drugs once delivered to the brain are cleared through metabolic processes within brain cells or via the cerebrospinal fluid (CSF), eventually re-entering the systemic circulation for elimination. The study's findings, indicated that the majority of activity resided in the abdomen despite the targeted brain delivery, suggesting that after exerting the therapeutic effects, zolmitriptan is likely metabolised and cleared through systemic pathways.

Similar results were reported in later studies to ascertain the delivery of MEs of clonazepam, sumatriptan, risperidone and cabergoline, as well as tizanidine hydrochloride-loaded thiolated chitosan NPs to the brain following IN administration 26-30. SPECT imaging of intranasally administered [^99m^Tc] radiolabelled tramadol hydrochloride microspheres confirmed significant and extended radioactivity accumulation in the rabbit's brain, demonstrating effective CNS targeting 31.

Jogani and co-workers 32 investigated the direct delivery of [^99m^Tc]-labelled tacrine, a cholinesterase inhibitor, to the brain via IN administration using a micropipette-assisted IN administration in for the treatment of Alzheimer's disease. The volume of the drug formulation given via the IN route was 5 µL per nare for mice, and 50 µL per nare for rabbits. The investigation involved using a tacrine solution in propylene glycol, radiolabelled with [^99m^Tc], via IN administration and IV injection in mice. The DTE% for IN administration was ~207, and the brain drug DTP% was ~52%, indicating that a significant portion of tacrine was directly transported to the brain from the nasal cavity. Additionally, brain scintigraphy imaging in rabbits confirmed higher brain uptake after IN administration than IV injection. The results demonstrated that IN delivery of tacrine led to faster peak times (T_max_ 60 min) in the brain, higher brain/blood ratios, and significant direct transport from the nasal cavity to the brain. These findings suggest that IN administration could enhance tacrine's bioavailability, reduce hepatotoxicity, and minimise exposure to non-target tissues, offering a potentially effective approach for Alzheimer's treatment. Notably, [^99m^Tc] was observed in the brain as early as 15 min post-administration, peaking at 60 min, and detectable up to 480 min later. The molecular weight and lipophilicity play a crucial role in the biodistribution of drugs, particularly in nose-to-brain delivery systems. Tacrine, a low molecular weight (235 kDa) and highly lipophilic drug (log P 2.71), is expected to travel via the extraneuronal epithelial pathway for direct nose-to-brain delivery. The elimination of the tracer from the brain involved both systemic circulation and direct transport mechanisms, with a significant portion bypassing the BBB.

In 2012, Mustafa *et al.* assessed the IN administration of lipophilic [^99m^Tc]-labelled ropinirole to the brain in rabbits through the olfactory pathway using NE and its homogenised version 33. The focus was on the fate of these formulations in the CNS. Brain bioavailability was assessed using gamma scintigraphy in a dynamic model involving radiolabelled nanoformulations of [^99m^Tc]-NE-ropinirole and [^99m^Tc]-suspension ropinirole. The study highlighted the superior brain localisation and C_max_ achieved with [^99m^Tc]-NE-ropinirole compared to [^99m^Tc]-suspension ropinirole, suggesting that the formulation is directly transported from the nasal cavity into the CSF, bypassing the BBB. Comparative dynamic mobility of the different formulations of [^99m^Tc]-NE-ropinirole and [^99m^Tc]-suspension ropinirole was performed by IN administration in rabbits for 30 min. Continuous imaging of the head was performed to track drug mobility from nose-to-brain and systemic circulation. The imaging revealed that the maximum residence time in the nasal cavity was not more than 10 min, likely due to mucociliary clearance. After 15 min, the drug was almost entirely cleared from the cavity, but a clear black signal was observed in the head region, indicating brain uptake. The imaging analysis revealed a faster onset of action, with the optimised homogenised NEs achieving a T_max_ at ~6.7 min post-IN administration, whereas [^99m^Tc]-suspension ropinirole had a T_max_ at ~11.7 min. Homogenisation was found to significantly improve brain uptake of ropinirole, underscoring its potential in enhancing CNS drug delivery. The enhanced brain delivery is attributed to the lipophilic nature of the NE carriers, and the smaller size facilitated faster absorption through the olfactory neurons, a crucial factor in overcoming mucociliary clearance, which is a significant challenge in INDD. The suspension form, being less effective, highlights the importance of the formulation's physicochemical properties in biodistribution and efficacy in the nose-to-brain pathway. Moreover, due to the fast absorption and low dose volume, there was minimal escape of therapeutic molecules to the systemic circulation, resulting in low systemic bioavailability.

Another study explored IN administration vs IV injection of chitosan NPs as a delivery vehicle for [^99m^Tc]bromocriptine, a medication commonly utilised in Parkinson's disease treatment; with significant findings reported regarding the biodistribution and brain targeting efficiency of the tracer 34. The study utilised a micropipette to instil 5 µL of the drug formulation in each nostril, totalling 10 µL per administration. The biodistribution study revealed that the brain/blood concentration ratios were 0.47 for IN administration of [^99m^Tc]bromocriptine solution, 0.69 for IN administration of [^99m^Tc]bromocriptine-chitosan NPs, and 0.05 for the IV injection of the same NPs, measured at 0.5 h post-administration. The gamma scintigraphy results indicated that with IN administration, the bromocriptine-loaded chitosan NPs resulted in significantly higher DTE% of ~6 and a DTP% of ~84, increased bioavailability in brain tissue, and improved nose-to-brain delivery compared to solutions of ^99m^Tc-bromocriptine delivered via IN and IV injection. The quantitation of the tracer that reached the brain after IN administration showed that [^99m^Tc]bromocriptine-loaded chitosan NPs achieved a peak brain concentration of 0.15 % ID/g at 1 h post-administration, which declined to 0.03 % ID/g by 8 h, indicating gradual washout of the tracer from the brain. This enhancement in delivery was attributed to the mucoadhesive properties of the chitosan NPs, which extended residence time in the nasal cavity, resulting in improved permeation and sustained drug delivery to the brain. This mucoadhesive property allows the chitosan NPs to interact with the nasal mucosa, thereby decreasing mucociliary clearance and facilitating improved absorption through the olfactory and trigeminal pathways, bypassing the BBB. The observed elimination pattern suggests the tracer was likely cleared through the glymphatic system and CSF drainage pathways over time. These results collectively affirm the potential of chitosan NPs as an effective and non-invasive delivery system for brain-targeted therapies of Parkinson's disease.

The use of [^99m^Tc]-labelled carbamazepine, a drug used in the management of epilepsy, was investigated using ME formulation to compare IN administration vs IV injection in rats 35. In each nostril 10 µL was administered using a micropipette to ensure precise and effective delivery of the drug into the nasal cavity. When assessing for differences across the routes, the concentration of carbamazepine in the brain relative to the blood was consistently 2-3 times higher for up to 8 h following IN administration compared to IV injection. The half-life in the brain ranged between 2.76 and 3.55 h, indicating prolonged drug retention. Further analysis revealed that the carbamazepine mucoadhesive ME formulation achieved superior DTE% and DTP% when compared to other tested formulations, including carbamazepine ME and carbamazepine solution. The physicochemical properties, namely globule size, zeta potential, and inclusion of mucoadhesive agents, significantly influenced the distribution and effectiveness of the formulations. The study reported that the carbamazepine mucoadhesive ME had the highest DTE% and DTP%. Specifically, the mucoadhesive ME formulation exhibited a DTE% of ~241 and a DTP% of ~59, indicating that a substantial amount of the drug reaches the brain directly through IN administration. Notably, the mucoadhesive ME formulation exhibited a 2.20-fold increase in DTE% and a 6.62-fold increase in DTP% relative to the carbamazepine solution. Elimination of the tracer from the brain was gradual, with the elimination constant for the brain ranging from 0.19 to 0.25 and the half-life in the brain ranging from 2.76 to 3.55 h, suggesting a steady washout of the tracer over time. These findings underscore the benefits of the mucoadhesive ME formulation by significantly enhancing brain-targeting efficacy, primarily due to efficient direct transport from the nose-to-brain pathway. Gamma scintigraphy images of rats were acquired 0.5 h after IN instillation, and IV injection, revealing that uptake of radioactivity was substantially greater following IN administration of carbamazepine than IV injection. Within the IN-administration formulations, carbamazepine mucoadhesive ME formulation exhibited the highest levels of radioactivity compared to carbamazepine ME formulation and carbamazepine solution.

**Figure 3:**
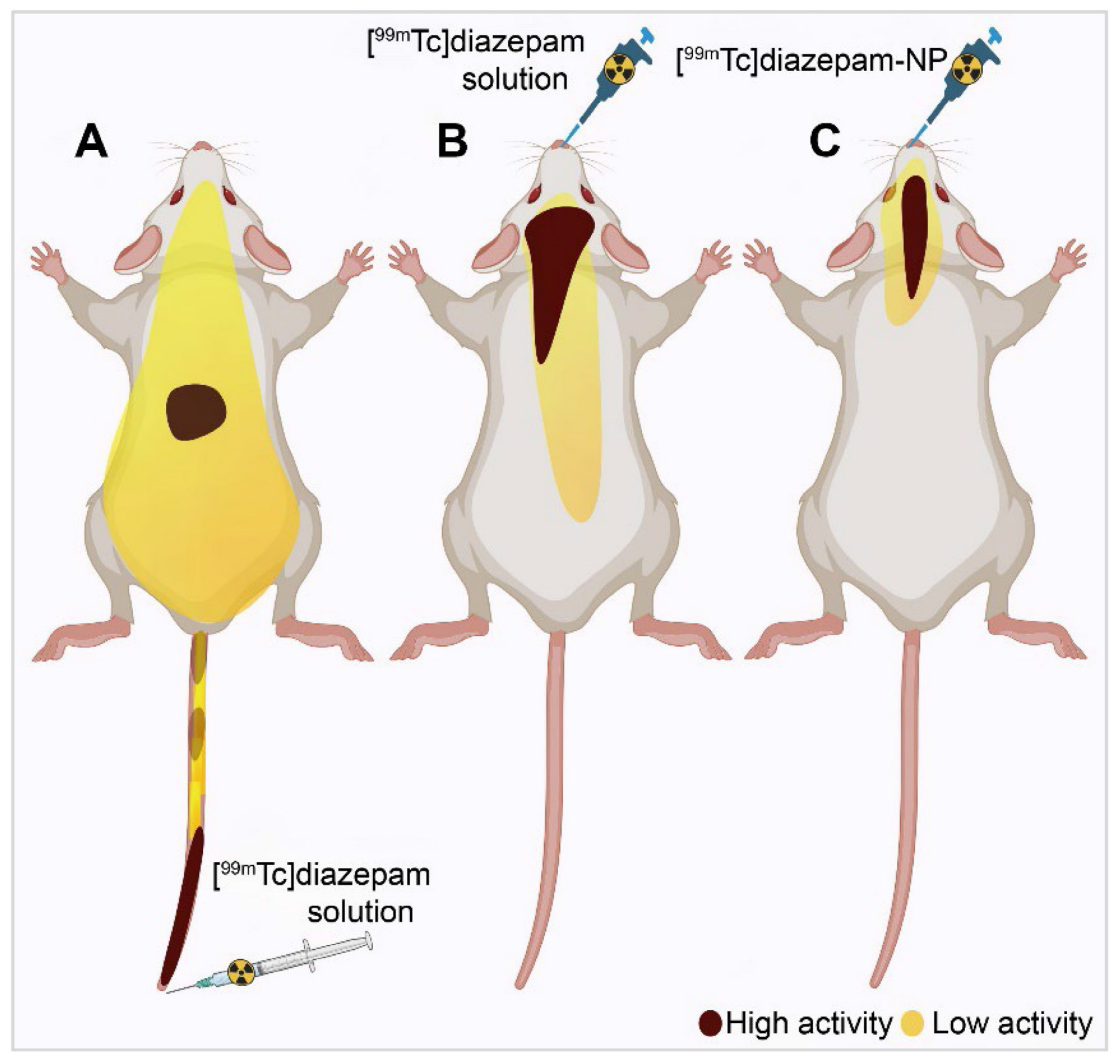
Gamma scintigraphy images of rats 30 min post-administration/injection. A: [^99m^Tc]diazepam solution post-IV injection. B: [^99m^Tc]diazepam solution post-IN administration. C: [^99m^Tc]diazepam-NP post-IN administration. Redrawn from 36. [created with BioRender.com].

Diazepam-loaded poly (lactic-co-glycolic acid; PLGA) NPs have been assessed in rats for efficiently delivering drugs directly to the brain intranasally. Each rat received 20 μL of the radiolabelled formulation, administered intranasally using a micropipette, with 10 μL of [^99m^Tc]diazepam solution and 10 μL of diazepam-NP administered in each nostril 36. The developed PLGA NPs (diazepam-NP) are nanoscale particles, and the spray droplets themselves are NPs. Gamma scintigraphy allowed for the visualisation and quantification of [^99m^Tc]diazepam-NP biodistribution, with a pronounced increase in radioactivity seen in the rat brain at 0.5 h post-IN administration, which demonstrated the superior uptake of [^99m^Tc]diazepam-NP via IN administration (e.g., 1.35 at 0.5 h), compared to [^99m^Tc]diazepam solution via both IV injection and IN administration (see Figure 3). Furthermore, biodistribution studies undertaken 8 h post-administration allowed tracking of drug persistence in the brain. The brain/blood ratio of the drug was highest for [^99m^Tc]diazepam-NP with IN administration across all the measured time points, underlining the potential of this route in maintaining a sustained drug presence in the brain. The NPs provided better DTE% and DTP%, with values of 258 and ~61, respectively. This important finding confirmed effective nose-to-brain transport of diazepam facilitated by a solution of PLGA-NPs, in rats. The active agents, in this case, diazepam, are encapsulated within these NPs, ensuring controlled release and targeted delivery to the brain.

In 2018, a series of notable studies investigated the applications of ^99m^Tc in INDD. The study by Mandlik developed and characterised zolmitriptan-loaded nanostructured polymeric carriers for targeted INDD 37. They administered 20 μL IN in mice using a micropipette. The SPECT/CT imaging results provided confirmation of the nanocarrier's enhanced brain uptake using IN administration. The biodistribution data consistently demonstrated the enhanced capability of [^99m^Tc]zolmitriptan-NP for targeted brain delivery. Specifically, the brain/blood ratio of IN administration of [^99m^Tc]zolmitriptan-NP 1 h post administration was found to be 5-fold higher than that using IV injection, and 3-fold higher than without the NP and using the IN-administration route. These significant fold increases underscore the potential value of nanostructured polymeric carriers in facilitating efficient nose-to-brain drug transport. Comparative analysis of C_max_ and area under the curve (AUC) in the brain for IN administration of [^99m^Tc]zolmitriptan-NP, IN administration of [^99m^Tc]zolmitriptan, and IV injection of [^99m^Tc]zolmitriptan-NP, demonstrated notably higher values for nose-to-brain targeting metrics, such as DTP% at ~6, DTE% at ~557, and direct nose-to-brain drug transport at 82%. The retention time of the tracer in the brain showed significant brain uptake for up to 8 h, with the highest concentration observed within the first-hour post-administration. The elimination of tracer from the brain was monitored over an 8 h period, showing a gradual decrease in brain concentration, implying the nanocarrier facilitated prolonged retention and gradual release of the drug in the brain tissue. The biodistribution of the imaging agents indicated that [^99m^Tc]zolmitriptan-NP had enhanced brain uptake due to its nanostructured polymeric carrier, suggesting efficient transport through the olfactory and trigeminal pathways. These findings underscore the potential value of nanostructured polymeric carriers in facilitating efficient nose-to-brain drug transport.

A second study focused on the use of levetiracetam, a selective synaptic vesicle glycoprotein 2A (SV2A) receptor antiepileptic, which was successfully radiolabelled with [^99m^Tc] for imaging the SV2A receptor 38. [^99m^Tc]levetiracetam was formulated into an ME with a small particle size (16.34 ± 5.58 nm) and favourable polydispersity index (0.382 ± 0.05). Comparative biodistribution studies assessed the DTE% of three formulations: IV injection of [^99m^Tc]levetiracetam solution, IN administration of both [^99m^Tc]levetiracetam solutions, and [^99m^Tc]levetiracetam-ME. Results indicated that the ME formulation exhibited significantly higher brain uptake and a superior brain/blood ratio at all measured time intervals, particularly at 5 min after administration, where the ratio was ~29, compared to ~9 and 0.0014 for the IN administration solution and IV injection solution, respectively. For the IN of [^99m^Tc]levetiracetam-ME, the brain uptake was ~4% ID/g at 5 min post-administration. The retention time of the [^99m^Tc]levetiracetam tracer in the brain was evaluated through biodistribution studies, showing significant brain uptake at all time intervals (5, 15, 30, and 60 min) with the IN administration of [^99m^Tc]levetiracetam-ME demonstrating the highest retention. The study utilised a simple IN administration technique to deliver the [^99m^Tc]levetiracetam-ME, typically involving the use of pipettes or nebulisers to promote accurate dosing. As the study categorised the imaging agents based on their formulation and administration routes. The IV injection of [^99m^Tc]levetiracetam solution exhibited low brain uptake due to its limited lipophilicity and inability to cross the BBB. The IN administration of [^99m^Tc]levetiracetam solution showed moderate brain uptake with lower efficiency compared to the MEs, likely due to limited absorption through the nasal mucosa. In contrast, the IN administration of [^99m^Tc]levetiracetam-ME demonstrated significantly higher brain uptake, attributed to the enhanced lipophilicity and favourable nanosize allowing direct transport through olfactory and trigeminal nerve pathways, bypassing the BBB. These findings suggest its potential as the first SPECT tracer for imaging SV2A receptors and highlight the advantage of using [^99m^Tc] due to its availability and suitable half-life, making it a more practical choice over other isotopes like [^11^C]levetiracetam for similar applications.

The third study developed PLGA NPs of baclofen, a neuropathic pain medication, demonstrating enhanced brain delivery and uptake of [^99m^Tc]baclofen-NP through IN administration 39. Gamma scintigraphy studies in rats demonstrated that maximum uptake was achieved using IN administration at 3 h post-administration, superseding both the IV injection and oral administration routes. Biodistribution studies measured the concentration of NPs in the brain and blood at various time points to 24 h. The maximum percentage of radioactivity (~4%) was observed at 3 h in the brains of rats administered [^99m^Tc]baclofen-PLGA-NPs intranasally, followed by ~3% in rats that were administered the same formulation intravenously, while oral administration showed minimal brain distribution. This distribution remained high for 24 h post-administration. IN administration resulted in significant brain uptake due to the PLGA NP polymer matrix, acting as a reservoir and enabling direct administration through the olfactory lobe, bypassing the BBB. The study also evaluated pharmacokinetic parameters, finding that the C_max_ (~4% ID/g) at 3 h for intranasally administered [^99m^Tc]baclofen-PLGA-NPs was higher in the brain than the C_max_ (~3% ID/g) at 3 h for IV injection. The AUC for the brain of rats administered [^99m^Tc]baclofen-PLGA-NPs intranasally was significantly higher 41% hours/g than for IV injection ~34% hours/g. Similarly, the C_max_ and AUC for blood were also higher for IN administration. The study also calculated DTE% and DTP%, with values of ~184 and ~46, respectively, for intranasally administered [^99m^Tc]baclofen-PLGA-NPs, indicating efficient targeting to the brain. Biodistribution studies showed that within 90 min, [^99m^Tc]baclofen-PLGA-NP levels spiked in the brain (~3% ID/g), suggesting an early onset of action. Blood samples revealed that the maximum radioactivity levels was at 90 min (~4% ID/g) for IV injection of [^99m^Tc]baclofen-PLGA-NPs, followed by ~3% ID/g for IV injection of [^99m^Tc]baclofen aqueous solution. IN administration led to maximum levels at 3 h for both [^99m^Tc]baclofen-PLGA-NPs (~2% ID/g) and [^99m^Tc]baclofen aqueous solution (~2% ID/g), as they were slowly absorbed into the bloodstream. In summary, the developed baclofen-loaded PLGA NPs exhibited enhanced brain delivery and prolonged retention compared to aqueous formulations. The *in vivo* gamma scintigraphy and biodistribution studies confirmed the suitability of PLGA NPs as carriers for baclofen to combat neuropathic pain, demonstrating the advantages of IN administration in bypassing the BBB and achieving efficient brain targeting.

In 2020, four studies were published that investigated the potential of ^99m^Tc radiolabelled compounds for advancing brain imaging and drug delivery for various neurological disorders. The first study was designed to formulate a radiolabelled version of olanzapine, an antipsychotic drug known for its limited permeability in the brain, while ensuring it was free from colloidal impurities 40. The research sought to assess its distribution within the body after being administered through IN administration and IV injection to determine its viability for brain imaging diagnostics. Post-administration of [^99m^Tc]olanzapine, imaging outcomes revealed significant brain uptake after both IN administration using a Hamilton syringe of 6.20 ID/g and IV injection of 5.50 ID/g, with optimal imaging obtained at 0.5 h post-IN administration, and 1 h post-IV injection 40. These high uptake values demonstrate the [^99m^Tc]olanzapine complex's capability to effectively concentrate and selectively localise within the brain. The [^99m^Tc]olanzapine complex achieved the highest brain/blood ratio of 4.70 ID/g at 0.25 h following IN administration. This ratio stayed > 1 for up to 1 h before gradually diminishing to ~1% ID/g at 8 h. Conversely, after IV injection, the brain/blood ratios slowly climbed to peak at a maximum of ~1% ID/g at 4 h. This ratio remained < 1, suggesting that post-IV injection, the [^99m^Tc]olanzapine complex is more prevalent in the blood than in the brain. Furthermore, the compound was rapidly cleared from most bodily organs, underscoring its potential for precise brain imaging. The kidneys were identified as the primary excretion route, as indicated by the high activity/uptake. The delivery mechanisms to the brain following IN administration were primarily through the olfactory and/or the trigeminal nerves, providing a direct route to the brain. Additionally, another portion was absorbed into the systemic circulation via transcellular diffusion through the nasal membrane, then crossing the BBB to reach the brain. The direct route contributed to the major fraction of the [^99m^Tc]olanzapine complex in the brain, while the indirect route provided a minor fraction. In a subsequent study, an NE encapsulating memantine, a drug used for Alzheimer's disease, was developed for IN administration in mice 41. This compound, a non-competitive NMDA (N-methyl-D-aspartate) receptor antagonist, has limited bioavailability due to first-pass metabolism, producing three main polar metabolites, each with minimal effects on NMDA receptors. The study compared the IN, IV, and oral administrations of [^99m^Tc]memantine-NE in rats, with gamma scintigraphy and biodistribution studies confirming superior brain uptake percentage radioactivity of ~4% ID/g at 1.5 h post-administration through IN administration using a micropipette, and also the highest concentration in the brain 41. Blood analysis revealed higher drug levels for the IN administration group at earlier time points. The study observed significant drug uptake in various brain regions including the olfactory bulb, cortex, and hippocampus, indicating successful delivery to target sites. The retention time of the tracer in the brain was observed up to 24 h post-administration. The memantine-loaded NE follows the olfactory and trigeminal pathways for direct nose-to-brain delivery, bypassing the BBB and resulting in higher drug concentrations in the brain. The washout process involves clearance through CSF flow, the glymphatic system, and efflux transporters at the BBB, with systemic absorption leading to metabolism and renal excretion. Transmission electron microscopy was used to confirm the localisation and distribution of the drug within the brain tissue. A third study focused on developing an NE formulation loaded with donepezil hydrochloride, also used in Alzheimer's disease treatment 42. Donepezil suffers from limited brain availability and peripheral side effects when administered orally. Rats receiving the [^99m^Tc]-radiolabelled donepezil NE formulation through the IN administration route using a micropipette exhibited the highest radioactivity in brain tissue at ~3% ID/g at 1.5 h post-administration, significantly surpassing the levels in rats given the aqueous formulation ~2% ID/g through IN administration. The study reported pharmacokinetic parameters, including the mean residence time of 12.75 h for the brain tissues of rats administered with the IN administration of [^99m^Tc]donepezil-NE, suggesting that the drug remains in the brain tissue for a considerable duration before being cleared. [^99m^Tc]donepezil-NE was absent in the brains of rats that received it orally. Minimal distribution of the agent was observed in the rat brain with IV injection, or the aqueous formulation via IN administration. The pharmacokinetic analysis demonstrated a gradual decrease in brain radioactivity over 24 h. Overall, the study suggests that the developed [^99m^Tc]-donepezil-NE formulation offers a promising approach for Alzheimer's disease treatment via IN delivery, achieving higher brain uptake and prolonged retention compared to conventional routes. The final study involved an NE formulation loaded with vitamin D3, designed for addressing cerebral ischemia 43. The administration of the ^99m^Tc-vitamin D3 NE was performed intranasally using a micropipette. Gamma scintigraphy confirmed a markedly higher deposition percentage of ~3% ID/g of the NE in the brain through IN administration versus ~1% ID/g only for IV injection of [^99m^Tc]vitamin D3 solution at all time points, peaking at 4 h. Notably, the IN administration route for the [^99m^Tc]vitamin D3 NE resulted in approximately 4-fold higher brain deposition, supported by radiometry assays. However, high radioactivity levels were detected in the liver, kidney, spleen, and heart following IV injection, potentially due to rapid absorption and biodistribution. A marked reduction in radioactivity accumulation was noted after 24 h in all samples, indicating the elimination of the radioactive complex from the body through natural metabolic pathways and substantive excretion. In conclusion, the vitamin D3-loaded NE formulated for IN delivery exhibited effective brain targeting, with significant brain uptake and reduced peripheral distribution. This approach demonstrated potential for treating cerebral ischemia by ensuring higher drug concentrations in the brain and minimising systemic exposure.

Most recently, two studies by Upadhaya *et al.* focused on developing an innovative INDD using radiolabelled micelles for diagnosing and treating CNS tumours, particularly gliomas 44, 45. The first study investigated radiolabelled folate-encapsulated micelles (folic acid tetraethylenepentamine conjugate (FA-TEPA) as a diagnostic aid 45. These micelles, designed for IN administration, target overexpressed folate receptors in CNS tumours. The folate conjugate, synthesised with a bifunctional chelating agent and radiolabelled with [^99m^Tc], showed high uptake in the brain (around 16% ID/g at 4 h). Studies in mice and rabbits demonstrated the micelles enhanced brain penetration and safety. The micellar carriers, owing to their nano size, mucoadhesive nature, and enhanced permeation, show significantly higher brain uptake compared to the radiolabelled folate solution, which was confirmed by biodistribution studies, which indicated that the brain uptake of the [^99m^Tc]FA-TEPA was significant over a period of 240 min, with enhanced uptake observed at all time points. SPECT imaging further validated the micelle's effective brain uptake. The stability of the formulation, mucoadhesivity, and biocompatibility make it a promising non-invasive diagnostic tool for brain tumours and potentially other folate-expressing cancers. Additionally, the study indicated higher retention in the brain for the micellar formulation compared to the solution. Histochemical analysis of nasal and brain tissues post-sacrifice after 28 days of dosing did not reveal any marked differences compared to control animals, indicating no significant toxicity or damage, supporting the safe use of the formulation for IN administration. The micellar formulation's mucoadhesive nature was confirmed by a mucous glycoprotein assay, which showed 95% mucoadhesion within 15 min, sufficient for efficient IN absorption given the nasal mucociliary clearance for an adult lies between 10 and 20 min. Overall, this study supports the potential of radiolabelled folate micelles as an effective diagnostic tool for CNS tumours and potentially other folate-expressing cancers, highlighting their significant brain uptake, safety, and non-invasive administration method. The second study explored nose-to-brain delivery of radiolabelled chemotherapeutic micelles, focusing on glioblastoma treatment 44. Methotrexate (MTX) was conjugated with a bifunctional chelating agent and labelled with [^99m^Tc]. The delivery device used to administer the tracer intranasally was a micropipette, and the volume of the drug formulation given via the IN route was 10 μL. The micelles demonstrated increased brain uptake and had a 3-fold enhanced anti-cancer activity compared to the IN solution. Organ biodistribution studies in mice revealed that the micelles preferentially accumulated in the brain, peaking at 120 min after IN administration with approximately 15% ID/g, compared to the IN solution, which showed approximately 3% ID/g. They also accumulated in the kidneys, lungs, blood and liver, with minimal activity in the gastrointestinal tract. SPECT imaging of rabbits confirmed the micelle's superior brain distribution. The study suggested combining radiotherapy with chemotherapy via this delivery system could significantly improve glioblastoma treatment outcomes. The elimination of tracer from the brain and other organs was observed through biodistribution studies, showing higher radioactivity in the kidneys, liver, and brain for the micellar formulation group, suggesting renal and hepatic excretion pathways. Histopathological analysis of the nasal epithelium, brain, and lungs indicated that the formulation was safe for nasal administration, with no significant differences between treated and control groups. Both studies emphasised the importance of delivery system characteristics, such as particle size, shape, and mucoadhesive properties, in achieving efficient and targeted INDD to the brain. The use of [^99m^Tc] in these systems enhances their diagnostic and therapeutic potential, providing a targeted, non-invasive, and effective approach to CNS tumour management.

#### 2.1.2. Rubidium-86 ([^86^Rb]), thallium-201 ([^201^TI]) and, manganese-54 ([^54^Mn])

Ionic balance in neurons is vital for brain function and is regulated by transport across the BBB, choroid plexus, and cell membranes of glial cells and neurons 46. The olfactory system, with receptor neurons in contact with both the nasal and cranial cavities, offers an alternative ion pathway for reaching the brain directly. These bipolar neurons, located in the olfactory epithelium beneath the cribriform plate of the ethmoid bone, have cilia extending toward the epithelium and axons toward the brain. Their axons pass through the cribriform plate to the olfactory bulb, where they synapse with mitral and tufted cells, and then extend to the primary olfactory cortex. This pathway allows proteins, viruses, and ions to enter the brain directly, bypassing blood circulation 47. Persson *et al.* showed that IN administration of radioactive cobalt-57 and zinc-65 led to their transport along the olfactory neurons to the olfactory bulb 48, 49. Similarly, Evans *et al.* observed that when cadmium-109 was applied intranasally, it primarily accumulated in the olfactory bulb on the application side, with a lesser extent in the opposite bulb 50. Further studies on pike, a type of fish known as Esox Lucius, with intranasally administered cadmium-109 indicated that cadmium was absorbed into the olfactory rosette, nerve, and the anterior part of the olfactory bulb. These findings suggested that cadmium is transported along the olfactory nerve, accumulating at the terminals of the olfactory axons, but it does not proceed along the transneuronal pathway 51. The transport was shown to be active, with a constant rate of approximately 2.38 mm/hr, and the cadmium strongly accumulated in the anterior olfactory bulb while other brain areas had minimal levels. This indicates a selective uptake and transport mechanism within the olfactory system.

A study aimed to investigate the olfactory transport route of Rb ions and Tl ions following IN administration 52. The delivery device used to administer the tracer intranasally was a Myjector, administering 15 µL of the drug formulation via the IN route. To elucidate the delivery and distribution within the olfactory system, radioactive [^86^Rb] chloride ^86^RbCl and [^201^TI] chloride ^201^TlCl solutions were utilised. The axonal transport of [^86^Rb] and [^201^TI] through the olfactory nerve pathway in mice utilising both IN administration and IV injection solutions were explored. Detailed analysis revealed the subsequent distribution of these radiotracers within various brain regions post-mortem. The retention time of the tracer in the brain showed significant accumulation in the olfactory bulb for up to 24 h, with progressive spreading to other brain regions within 12 h post-administration. The results indicated that within 6 h post-administration, there was a notably higher accumulation of these substances in the olfactory bulb on the ipsilateral side ([^86^Rb], 0.7% ID; [^201^Tl], 0.5% ID) compared to the contralateral side ([^86^Rb], 0.08% ID; [^201^Tl], 0.15% ID). This accumulation was observed to extend over time, with the transported [^86^Rb] and [^201^TI] progressively reaching other brain regions, such as the telencephalon and diencephalon, more prominently on the side of dose administration. Elimination of the tracer from the brain involved gradual decreases in concentration over time, with [^86^Rb] uptake rates showing a decrease after 6 h and further at 12 and 24 h post-administration. [^86^Rb] autoradiography after ipsilateral administration found high concentrations of [^86^Rb] were noted in the olfactory epithelium and olfactory bulb. In the coronal head sections of mice, there was a noticeable dense accumulation of [^86^Rb] on the right side, including areas around the hippocampus. In sagittal sections, there was a significant gathering of [^86^Rb] in the olfactory bulb 6 h post-administration, with subsequent spreading to further areas of the brain. Importantly, the [^86^Rb] was located within the brain tissue itself, not in the cranial cavities or ventricles. These findings suggest a pathway for [^86^Rb] transport from the nostril to deeper brain areas. [^201^TI] autoradiography found dense accumulation in specific areas of the olfactory bulb and cortex. These findings provide strong evidence of direct axonal transport of these substances from nose-to-brain via the olfactory pathway, potentially mirroring the transport behaviour of potassium in the olfactory system. Moreover, following IN administration, [^201^Tl] was observed to be absorbed by regions including the olfactory tract, olfactory cortex, thalamus, and hypothalamus. This study opens avenues for using such radiotracers in medical imaging, particularly for diagnosing conditions like anosmia and neurodegenerative diseases.

In a 2008 study, researchers examined the transport of [^201^TI] and [^54^Mn] ions in the olfactory nerve of mice 53. The study focused on understanding the IN administration of these ions and their subsequent transport to the olfactory bulb. The experiment utilised both control mice and a transected olfactory nerve fibre model, to whom a double tracer solution of ^201^TlCl and ^54^MnCl_2_ was applied via IN administration. The delivery device used was a micropipette, and the volume of the double tracer solution administered was 20 µL. Uptake of these tracers was measured in the olfactory bulb and other organs three hours post-administration. Uptake of these tracers was measured in the olfactory bulb and other organs 3 h post-administration. A significant reduction in the uptake of both [^201^TI] and [^54^Mn] in the olfactory bulb was found compared to the control group, indicating that the transection of olfactory nerve fibres impedes the transport of these ions. The biodistribution of [^201^Tl] and [^54^Mn] was dependent on their physicochemical properties as alkali and transition metal ions, respectively. Both tracers were transported to the olfactory bulb, but their uptake was significantly reduced in the transected nerve model, indicating their reliance on olfactory nerve fibres for transport. Furthermore, the autoradiographic analysis showed marked inhibition in the transport of [^201^Tl] and [^54^Mn] from the nasal epithelium to the olfactory bulb, highlighting the role of olfactory nerve fibres in this process. This study suggested the potential diagnostic applications for assessing olfactory nerve damage, thereby contributing valuable insights into both neuroscientific research and clinical practice.

In 2011, Shiga *et al.* investigated the transport of IN [^201^TI] to the brain using SPECT/MRI hybrid imaging technique 54. Healthy volunteers with normal olfactory thresholds were enrolled, and ^201^TlCl saline solution was applied via IN administration. The delivery device used for administering the tracer intranasally was a syringe, which instilled 0.3 mL of ^201^TlCl saline solution into the olfactory cleft in either the right or left nasal cavity. The uptake of ^201^TlCl was first confined to the olfactory epithelium area 30 min post-administration, then spread to the intracranial space in the anterior skull base through the cribriform lamina after 24 h. This indicated that the peak uptake of ^201^Tl entered the olfactory bulb in the anterior skull base through the cribriform lamina 24 h after nasal administration. In this study, ^201^Tl was initially concentrated in the olfactory epithelium and later transported to the olfactory bulb through the olfactory nerve pathway. This pattern underscores the olfactory nerve axonal transport as the primary IN delivery pathway for this agent. The findings also highlight the potential infectious routes for viruses like influenza, and SARS/COVID, to the CNS via the olfactory pathway, with recent evidence pointing towards the distinct possibility of viral infections affecting the brain through the olfactory nerve transport route 55, 56. The study discussed the potential for using the IN administration of ^201^TlCl followed by SPECT/MRI imaging in patients with upper respiratory tract infections and hyposmia to assess viral damage to the olfactory pathway. The study further contributed to the understanding of the infectious route of prion proteins to the CNS, suggesting the olfactory pathway as a plausible route. These results provide the first images of olfactory transport to the anterior skull base in humans and highlight the need for future studies to establish viral damage in patients with upper respiratory tract infections and to further explore the olfactory nerve's role in the transmission of pathogens to the CNS. This study underscores the importance of understanding nose-to-brain transport mechanisms for developing diagnostic and therapeutic strategies for neurological diseases.

A series of studies by Shiga and the team explored the use of [^201^Tl] in olfactory imaging to assess and understand various olfactory disorders 57. The delivery device used in the study was a syringe, which was used to administer a 0.3 mL saline solution of [^201^Tl] into the olfactory cleft of the nasal cavity. The uptake of [^201^Tl] was assessed 24 h after IN administration using SPECT scans. At this time, the tracer's presence in the olfactory bulb was observed, however, most of the [^201^Tl] had migrated to the nasopharyngeal region and was swallowed, resulting in minimal systemic effects due to the low CNS absorption of [^201^Tl]. The migration of [^201^Tl] to the olfactory bulb was significantly lower in patients with olfactory impairments caused by head trauma, upper respiratory infections, or chronic rhinosinusitis compared to healthy volunteers. Representative migration values included a 60-year-old healthy male volunteer who had a high migration level of 29%. In contrast, a 44-year-old female with head trauma showed a lower migration value of 4.2%, a 42-year-old female with an upper respiratory tract infection had a migration value of 4.5%, and a 67-year-old female with chronic rhinosinusitis had a migration value of 5%. This technique proved valuable in predicting recovery in patients with idiopathic olfactory disorders, where higher [^201^Tl] migration indicated a better prognosis. Patients with idiopathic olfactory dysfunction who showed high nasal migration of [^201^Tl] to the olfactory bulb recovered their sense of smell more quickly than those with low migration. The findings indicate that severe damage to the olfactory nerve, as detected by [^201^Tl]-based olfactory imaging, is associated with a poorer prognosis for patients experiencing loss of smell.

Their second study was to see if this imaging technique could predict recovery outcomes following treatment 58. The study involved 24 patients (7 women and 17 men, aged 23-73 years) with idiopathic olfactory dysfunction. These patients were retrospectively analysed based on their [^201^TI]-based olfactory imaging results prior to receiving conventional treatment with the Japanese herbal medicine tokishakuyakusan. The delivery mechanism involved the IN administration of 0.3 mL of [^201^Tl] into the olfactory cleft. The SPECT/CT imaging assessed the migration of [^201^Tl] to the olfactory bulb 24 h post-administration. Additionally, MRI was used in conjunction with SPECT/CT to accurately delineate the olfactory bulb and assess its volume. The key findings indicated that high [^201^Tl] migration to the olfactory bulb ≥ 4.6% was significantly correlated with a better prognosis and shorter recovery duration. Patients with high [^201^Tl] migration had a 67% recovery rate 1 year after treatment, compared to 0% in patients with low migration. The extent of [^201^Tl] migration served as a reliable prognostic marker for recovery in idiopathic olfactory dysfunction, suggesting that patients with intact olfactory nerve fibres could be identified using this imaging technique for targeted follow-up and treatment. Other potential prognostic factors, including gender, age, smoking habit, parosmia, and olfactory bulb volume, were not significantly correlated with prognosis in this study. The researchers discussed the need for a more rapid olfactory nerve tracer for clinical applications, as the current 24 h assessment period for [^201^Tl] migration requires patients to return to the hospital for imaging. They highlighted [^125^I] conjugated human recombinant insulin-like growth factor-1 (IGF-1) as a potential alternative. This tracer can be detected in the mouse cerebrum 30 min after IN administration and is prevented from increasing certain brain activities if the olfactory bulb is damaged, indicating its transport through the olfactory nerve 59. However, [^125^I] with its long half-life and low energy make it unsuitable for clinical SPECT imaging, suggesting the need for developing new isotope-conjugated tracers for IN administration.

Further research highlighted the variation in [^201^Tl] migration in patients with parosmia following upper respiratory tract infections, revealing higher nasal [^201^Tl] uptake in the olfactory cleft in those without hyposmia 60. However, this increased uptake did not translate to significant differences in [^201^Tl] migration to the olfactory bulb between groups with and without hyposmia. The olfactory bulb volume was notably larger in the parosmia-only group, although still below the normal range, suggesting specific impacts on olfactory structures under different conditions.

In a later study, the authors discovered that [^201^Tl] migration increased in scenarios where olfactory sensory neurons experienced reduced pre-synaptic inhibition from dopaminergic interneurons 61. These findings collectively demonstrate the effectiveness of [^201^Tl]-based olfactory imaging in evaluating olfactory nerve connectivity, providing prognostic insights for olfactory dysfunction, and understanding the neurobiological mechanisms underpinning olfactory processes that can affect the nose-to-brain pathway.

#### 2.1.3. Iodine radioisotopes ([^125^I], [^123^I], [^131^I])

The IN administration of [^125^I] labelled compounds presents a viable approach for non-invasive CNS imaging and targeted INDD. The IN application of targeting and trafficking mechanisms of cholera toxin (Ct) and its B subunit (CB) when administered intranasally, focusing on their interaction with the CNS, particularly the olfactory nerves/epithelium and olfactory bulbs 62. Utilising specific pathogen-free mice, the researchers tested radiolabelled Ct, CB, tetanus toxoid (TT), and ovalbumin (OVA), with a particular emphasis on Ct's role as a mucosal adjuvant labelled with [^125^I]. The delivery device used in the study for IN administration was a micropipette. The IN administration of these substances was analysed to determine their dispersal and retention across various tissues. [^125^I]-labelled CB was administered intranasally, and its presence was tracked in both lymphoid (nasopharyngeal-associated lymphoreticular tissue, cervical lymph nodes, mesenteric lymph nodes, spleen) and neuronal tissues (olfactory nerves/epithelium, olfactory bulbs, brain). The CB appeared in the peripheral blood within 15 min, accounting for approximately 50% ID/g. The excretion of [^125^I]CB from the blood and spleen followed similar patterns, returning to baseline levels within 48 h. There was a notable initial accumulation of [^125^I]CB in the nasopharyngeal-associated lymphoreticular tissue, although less than 0.02% ID/g of it remained in this tissue after six days. The radioactivity levels of [^125^I]CB in the cervical lymph nodes reached their highest between 1 and 2 h post-administration, showing a distinct profile from that observed in the blood, spleen, or nasopharyngeal-associated lymphoreticular tissue. The slower decline of [^125^I]CB in the cervical lymph nodes compared to the nasopharyngeal-associated lymphoreticular tissue suggests a typical antigen uptake route in the nasopharyngeal-associated lymphoreticular tissue followed by movement into the cervical lymph nodes. The olfactory nerves/epithelium and the olfactory bulbs exhibited a distinct pattern of CB accumulation unlike what was observed in lymphoid tissues. The radioactivity levels in the olfactory nerves/epithelium reached a peak at 6 h, then stabilised, and sustained over a period of 6 days. In contrast, the levels of [^125^I]CB in the olfactory bulbs peaked at 15 min and maintained a relatively steady state thereafter. Moreover, the olfactory nerves/epithelium and olfactory bulbs were the only tissues where this unique accumulation pattern of [^125^I]CB was noted. The radioactivity levels of [^125^I]CB in the brain followed a trajectory similar to those associated with CB in the blood, dropping to below 50 CB-specific counts per minute (cpm) by 24 h. When analysing the concentration of [^125^I]CB relative to the organ weight, the disparities in tissue association became even clearer. In the olfactory bulb, the levels of CB were about 34 times higher, and in the olfactory nerves/epithelium, they were approximately 620 times higher than those seen in the brain per milligram of tissue. This suggests that the pronounced accumulation of CB in these areas likely occurred through binding to monosialoganglioside (GM1), given that no similar accumulation was observed when TT or OVA were administered intranasally. Furthermore, the biodistribution of [^125^I]Ct holotoxin after IN administration, was somewhat similar yet slightly different in dynamics from that of [^125^I]CB. Initially, the [^125^I]Ct showed a delayed peak in blood levels but experienced a significant decrease within 12 to 24 h post-administration. Alternatively, most tissues including cervical lymph nodes, blood, spleen, and brain showed a later peak in [^125^I]Ct, which remained elevated longer than the levels of [^125^I]CB. An exception was the nasopharyngeal-associated lymphoreticular tissue, which displayed very similar clearance kinetics to the CB. Notably, the olfactory nerves/epithelium bound higher levels of the Ct compared to the CB at 1.5 h. The levels of [^125^I]Ct then decreased rapidly within the first 24 h and subsequently declined more slowly over the next six days, a similar observation also noted with the CB. The residence of both Ct and CB in the olfactory bulbs followed similar patterns in the olfactory bulbs throughout the six days tested. This consistent pattern in both forms suggests that neuronal binding is a distinctive feature of Ct. Additionally, to investigate whether Ct when used as a mucosal adjuvant can enhance the delivery of protein vaccines into neuronal tissues, non-labelled holotoxin was administered intranasally along with [^125^I]TT. After IN administration of [^125^I]TT, the distribution across various tissues was assessed and compared with that of [^125^I]TT administered on its own. A slower clearance of [^125^I]TT was noted in both lymphoid and CNS tissues. Elevated levels of [^125^I]TT were observed at 24 and 48 h, which then gradually decreased over a six-day period. Notably, there were significant increases in [^125^I]TT concentrations in the olfactory nerves/epithelium from 12 to 48 h post-administration when Ct was used, unlike when [^125^I]TT was administered alone.

In a study targeting Alzheimer's disease, researchers used basic fibroblast growth factor (bFGF) labelled with [^125^I] 63. Since amyloid-beta (Aβ) plaques are a hallmark of Alzheimer's disease and these deposits are rich in heparan sulfate proteoglycans, which can bind to bFGF and serum amyloid P components, the researchers explored a new method of ligand delivery to the brain. This method was tested to detect Aβ deposition in a transgenic mouse model that overexpressed the Aβ-protein precursor. The delivery device used was a tapered-end plastic tip, and each mouse received six drops (1.5 µL each) of [^125^I]-bFGF intranasally. This method utilises the olfactory pathway to bypass the BBB, allowing direct access to the brain through the cribriform plate due to the open intercellular clefts in olfactory epithelial cells. Significant brain uptake of [^125^I]-bFGF was observed 3 h after administration, with notable uptake in the olfactory bulb, frontal cortex, parietal cortex, hippocampus, and cerebellum. Transgenic mice exhibited 3-5 fold higher uptake in these regions compared to wild-type controls. The study compared bFGF and serum amyloid P components, and their distribution in the brain. Serum amyloid P components demonstrated stronger binding to amyloid plaques, especially in the cortex and microvessels, compared to bFGF, which showed intense staining in neurons around Aβ deposits in the frontal, parietal, and occipital regions. This higher intensity of serum amyloid P components staining indicates its higher affinity for amyloid plaques relative to bFGF. The study also noted significant aspiration into the trachea and stomach, suggesting that much of the intranasally administered bFGF entered the respiratory and digestive tracts. Interestingly, IV injection did not result in significant brain uptake, underscoring the importance of the IN route for targeting the CNS. High-resolution histochemical findings included light microscopy showing specific binding of intranasally administered serum amyloid P components and bFGF to amyloid plaques. Serum amyloid P components demonstrated more intense immunostaining than bFGF. Electron microscopy confirmed the presence of bFGF within amyloid plaques and associated neuronal structures in transgenic mice. In conclusion, this study presents a promising non-invasive method for labelling Aβ deposits using IN administration of bFGF and serum amyloid P components. The results support the potential use of these agents for future imaging studies in Alzheimer's disease, providing a foundation for developing diagnostic tools and evaluating therapeutic interventions, potentially adaptable for human use with IN administration of radiolabelled ligands and subsequent imaging using SPECT or PET techniques.

A study investigated the delivery of IGF-1, a 7.65 kDa protein neurotrophic factor, to the rat brain and spinal cord via IN administration, focusing on the pathways and mechanisms involved in its transport from the nasal passages to the CNS. The delivery device used was a micropipette, administering a total of 50 μL [^125^I]IGF-1 solution. [^125^I]IGF-1 shows rapid entry into the CNS (within 30 min) 64. The study identified two primary delivery pathways for IGF-1: the peripheral olfactory system, which connects the nasal passages with the olfactory bulbs and rostral brain regions, and the peripheral trigeminal system, which connects the nasal passages with the brainstem and spinal cord regions. Delivery mechanisms included extracellular transport through intercellular clefts and intracellular transport via adsorptive endocytosis into olfactory sensory neurons. Gamma imaging found that the uptake of [^125^I]IGF-1 was measured 30 min post-administration, with CNS levels peaking earlier than blood levels. Key uptake values in important CNS regions included the olfactory bulb (3.43 nM), anterior olfactory nucleus (1.65 nM), frontal pole (1.44 nM), motor cortex (1.33 nM), olfactory tubercle (1.08 nM), caudoputamen (0.32 nM), hippocampal formation (0.34 nM), diencephalon (0.46 nM), midbrain (0.39 nM), cerebellum (0.40 nM), pons (0.41 nM), medulla (0.87 nM), cervical spinal cord (2.39 nM), thoracic spinal cord (0.15 nM), and lumbosacral spinal cord (0.078 nM). The study demonstrated that IN administration resulted in significantly higher CNS concentrations compared to IV injection, indicating effective bypassing of the BBB. Retention of [^125^I]IGF-1 in the CNS was evident up to 24 h post-administration, with a gradual decline in concentrations over time. Elimination pathways involved significant targeting of the deep cervical lymph nodes, suggesting a role in lymphatic drainage from both nasal passages and CNS. No detectable radioactivity was found in cisternal CSF, indicating limited penetration into CSF from the bloodstream or nasal passages within the initial 30 min. Clearance patterns showed a gradient with CNS concentrations decreasing over time and distance from entry points, reflecting slow and prolonged absorption into the bloodstream and subsequent clearance. High-resolution phosphor imaging revealed a broad distribution of [^125^I]IGF-1 throughout the CNS, with the highest amounts near the olfactory and trigeminal regions. The biodistribution of [^125^I]IGF-1 in a high-specific activity solution also matched well with known IGF-1 binding sites in the adult rat CNS, indicating that the IGF-1 reached the brain and spinal cord intact. Immunohistochemical analysis showed enhanced phosphotyrosine immunoreactivity in specific CNS regions following IN IGF-1 administration, indicating biological activity and receptor activation. Overall, the study systematically evaluated the localisation of [^125^I]IGF-1 in various CNS regions, supported by high-resolution imaging and immunohistochemical confirmation, demonstrating the effectiveness of non-invasive IN delivery for therapeutic proteins to the CNS.

In a subsequent study, Thorne *et al.* investigated how interferon-β1b (IFN-β1b), a protein used to treat the relapsing-remitting form of multiple sclerosis, affects the nervous system of monkeys following IN administration using plastic tubing, the volume administered was between 0.9 to 1.0 mL 65. Five monkeys received an IN administration of [^125^I]IFN-β1b into the upper nasal regions. The delivery pathway for [^125^I]IFN-β1b primarily involved the olfactory and trigeminal nerves. The mechanisms underpinning this transport involved the rapid extracellular movement of the tracer along nerve components in the nasal epithelium to the olfactory bulb and brainstem. From these entry points, the tracer dispersed to other CNS regions via pulsatile flow within the perivascular spaces of cerebral blood vessels. The uptake time for [^125^I]IFN-β1b was relatively rapid. Blood levels of the tracer began to rise shortly after administration, reaching peak concentrations approximately 30 to 45 min post-administration. High-resolution imaging and gamma counting of micro-dissected tissue samples revealed that significant tracer concentrations were present in the brain and spinal cord within 53 to 59 min of administration. This rapid uptake underscores the efficiency of the IN-delivery method for transporting therapeutic proteins to the brain. Upon reaching the CNS, [^125^I]IFN-β1b showed distinct patterns of distribution. The highest uptake was observed in the olfactory bulbs (16,600 pM), and trigeminal nerves (16,300 pM), indicating these regions as primary entry points. Significant levels of the tracer were also found in the basal ganglia, including the caudate, putamen, globus pallidus, and substantia nigra. These findings suggest a preferential localisation of the tracer in areas associated with dopamine regulation, which may have clinical implications for understanding the side effects of IFN-β1b therapies. Intermediate levels of uptake were noted in the hippocampal formation, thalamus, cerebellum, and brainstem nuclei of several cranial nerves. Lower uptake levels were observed in CNS fibre pathways such as the cerebral white matter and corpus callosum. Quantification of the tracer that actually reached the brain revealed that approximately 0.0064% ID of [^125^I]IFN-β1b was present in the brain and spinal cord. Despite this seemingly low delivery efficiency, the concentrations achieved were sufficient to elicit biological effects, given the high potency of IFN-β1b. Elimination of the tracer from the brain primarily involved peripheral clearance mechanisms, with the kidneys and liver playing major roles. High radioactivity levels in the thyroid gland indicated significant peripheral deiodination and metabolism of the tracer, further corroborating the efficient systemic clearance observed. High-resolution histochemical findings from autoradiography provided detailed insights into the anatomical distribution of [^125^I]IFN-β1b. The imaging confirmed high signal intensities in the basal ganglia, substantia nigra, and cerebellum, aligning with gamma counting data. While direct histochemical analysis was not performed in this study, the imaging data strongly suggest a correlation with potential histochemical localisation, particularly in the basal ganglia. In conclusion, this study demonstrates the effective targeting and retention of [^125^I]IFN-β1b in the CNS following IN administration. The significant uptake in the basal ganglia suggests potential clinical relevance for IFN-β1b therapies, particularly concerning their side effects.

A study examining the feasibility and efficacy of delivering vascular endothelial growth factor (VEGF) labelled with [^125^I] following IN administration reveals extensive distribution within the CNS using a micropipette 66. Gamma counting and autoradiography were employed to quantify and visualise the distribution of [^125^I]-VEGF within the brain. The highest concentrations of the tracer were observed in the trigeminal nerve and optic nerve, followed by significant uptake in the olfactory bulbs, olfactory tubercle, striatum, medulla, frontal cortex, midbrain, pons, appendix cerebri, thalamus, hippocampus, and cerebellum. The autoradiographic analysis provided high-resolution images, confirming the widespread distribution of VEGF throughout the CNS. The uptake of [^125^I]-VEGF was rapid, with substantial delivery observed approximately 30 min after IN-administration. The retention time was not explicitly detailed, but significant levels of VEGF were present within 30 min timeframe. Quantitatively, the % ID/g was highest in the trigeminal nerve (1.57), followed by the olfactory bulb (0.64) and optic nerve (0.96). These findings underscore the efficiency of the IN route in targeting specific CNS regions. The study found no detectable radioactivity in the CSF following IN administration, suggesting that VEGF might be cleared via lymphatic pathways rather than through the CSF. This was further supported by the high concentrations of [^125^I]-VEGF found in the deep cervical lymph nodes, indicating ready access to nasal lymphatics and supporting a direct pathway from the nasal submucosa to brain interstitial fluid. Autoradiography corroborated high-resolution histochemical findings, which confirmed the presence and detailed distribution of [^125^I]-VEGF in various brain regions. This imaging provided a robust validation of the IN-delivery pathway, highlighting its potential to effectively deliver therapeutic agents to the CNS. The study's findings were further reinforced by comparisons with IV injection, which resulted in significantly lower CNS concentrations and higher peripheral tissue exposure, emphasising the superiority of the IN route.

Another study on wheat germ agglutinin conjugated NPs (WGA-NPs), labelled with [^125^I], and upon IN administration of 50 μL using a polyethylene tube attached to a micro syringe, demonstrates their fast transcellular absorption (5 min) to the olfactory bulb through the olfactory epithelium 67. Post entry into the lamina propria, a portion of the labelled compound moved from the olfactory nerve bundles to the olfactory bulb. Here, the trigeminal nerves played a role in distributing the compound to caudal brain regions, with notable radioactivity concentrations found of ~128 cpm/g tissue/g dose (5 min post-administration. WGA-NPs entered the brain via extracellular transport along the trigeminal nerves, which connect to the brainstem at the pons and extend to the cervical spinal cord and medulla. The study found rapid NP uptake within 5 min, with peak levels observed at 30 min. By 2 h post-administration, the fluorescence intensity decreased significantly, indicating clearance. The primary regions of uptake included the olfactory bulb, specifically the glomerular layer and external plexiform layer, as well as regions along the trigeminal nerve, such as the cervical spinal cord, medulla, and pons. However, the CSF pathway seemed to have a minimal role in transporting the compound to the CNS following IN administration, as very low radioactivity levels were detected in the CSF, comparable to those in the diencephalon and hippocampus, which were far below those in the olfactory bulb and trigeminal nerve. The uptake in the olfactory bulb reached a peak at 30 min post-administration (~11 cpm/g tissue/g dose) before decreasing, indicating rapid clearance from this region. Strong green fluorescence signals were observed in the olfactory epithelium and olfactory bulb, confirming successful uptake and transport. Immunohistochemistry staining with anti-nerve specific enolase and anti-ZO-1 antibody verified that the uptake was primarily transcellular through the olfactory epithelium. Comparative analysis of imaging agents used in the study included coumarin-6 (Cou-6) for visualising NPs in the nasal cavity and olfactory bulb by fluorescence microscopy, ^125^I-labelled WGA-NP for measuring radioactivity levels to track distribution, and DiR (1,1'-dioctadecyl-3,3,3',3'-tetramethylindotricarbocyanine iodide) loaded NPs for *ex vivo* imaging to visualise brain transfer. The study found that WGA-NP showed enhanced uptake in the olfactory epithelium and higher distribution in the olfactory bulb and associated brain regions compared to unmodified NPs, which followed similar pathways but were less efficient in brain delivery. In conclusion, IN administration of WGA-NP effectively delivers NPs to the brain via olfactory and trigeminal pathways. The study provided detailed insights into the mechanisms of uptake, regional distribution, and retention of NPs in the brain, supported by high-resolution imaging and histochemical analyses. These findings contribute valuable information for designing effective nanocarriers for INDD.

Haloperidol, a dopamine-blocking agent used in brain imaging studies, demonstrates varied brain uptake 68. The focus is on the effectiveness of different delivery methods, including IV, IN-solution, and IN ME. The objective was to determine the optimal method for delivering ^125^I-haloperidol to the brain, thereby facilitating enhanced brain imaging. The delivery of ^125^I-haloperidol was executed using a small syringe for IN administration of 20 µL. The study found that the uptake time and brain regions affected varied significantly across the three delivery methods. For the IV route, brain uptake was initially observed at 5 min (0.5%), peaking at 15 min (1.4%), before declining. The IN-solution method showed a brain uptake of 3.7% at 5 min, peaking at 4.3% at 15 min, and then gradually decreasing. The IN ME method demonstrated the highest efficacy, with brain uptake observed at 3.8% at 5 min, peaking at 5.7% at 15 min, and maintaining substantial levels throughout the observed period. The primary regions of uptake included the olfactory bulbs, frontal cortex, and other brain areas connected to the olfactory and trigeminal pathways. Retention time analysis revealed that the IV route had the least retention, with brain uptake significantly decreasing over 120 min. The IN-solution method showed better retention, with notable uptake still present at 120 min. However, the IN ME method exhibited the highest retention, maintaining significant brain uptake across all time intervals, indicating prolonged retention and efficient targeting. The tracer's elimination from the brain followed a systemic pathway, with the tracer gradually washing out into the bloodstream over time. The data showed a decrease in brain uptake, indicating the washout process, which was more prolonged in the IN ME method, providing extended imaging windows. Collectively, these studies highlight the potential of IN administration [^125^I] imaging as a method for site-specific targeting within the CNS, offering insights into the dynamics of compound distribution and clearance from various brain regions.

**Figure 4:**
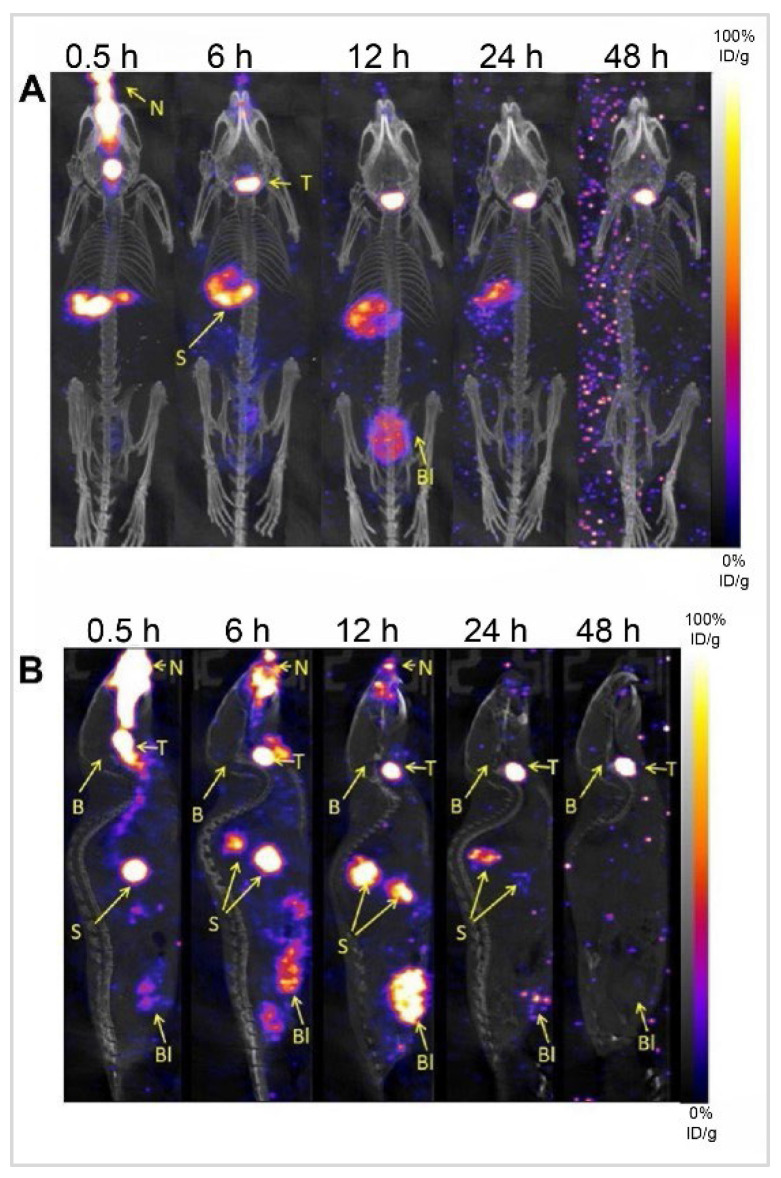
SPECT/CT fusion images of mice following IN administration of [^123^I]R8-YAβ(25-35)-PEI. A: displays the maximum intensity projection view. B: shows the sagittal view, marking 'B' for brain, 'Bl' for bladder, 'N' for nose, 'T' for thyroid and 'S' for stomach. Adapted with permission from 69, copyright 2022 Elsevier.

In a 2022 study by Wei-Lin Lo *et al*., the effectiveness of [^123^I]-radiolabelled anti-Aβ peptides for IN administration targeting the brain as a potential treatment for Alzheimer's disease was investigated 69. The study involved administering 5 µL of R8-YAβ(25-35)-PEI labelled with [^123^I] intranasally by dropper and then monitoring its distribution across the body with SPECT/CT imaging (see Figure 4). Peak uptake in the brain occurred at 0.5 h post-treatment, with a concentration of 0.73 %ID/g. The uptake decreased over time, showing values of 0.45% ID/g at 6 h, 0.28% ID/g at 12 h, 0.17% ID/g at 24 h, and 0.1% ID/g at 48 h. The highest uptake was observed in the olfactory bulb, indicating that the peptide initially enters the brain through this region before diffusing to other parts of the brain. Furthermore, the study delineated the peptide's neuro-targeting efficiency for nose-to-brain up to 1.78% ID/g tissue, with a calculated biological half-life of the peptide in the brain of approximately 10.2 h. Most of the [^123^I]R8-YAβ(25-35)-PEI solution was swallowed and passed through the oesophagus into the gastrointestinal tract, as observed by the gradual decrease in radioactivity in the brain and the increase in radioactivity in the bladder over time. Overall, the study demonstrates that IN administration of the [^123^I]R8-YAβ(25-35)-PEI peptide is an effective method for delivering peptides to the brain, with a notable brain uptake rate and retention time. This method holds promise for treating neurodegenerative diseases by providing a non-invasive route to bypass the BBB.

[^131^I] was used in a study by Motaleb *et al.* focusing on developing [^131^I]-trazodone hydrochloride as a new brain imaging radiopharmaceutical and establishing its efficacy 70. The study investigated the biodistribution and brain uptake of [^131^I]-trazodone using different administration routes, specifically IV, IN solution, and IN ME. The study showed that the brain uptake of [^131^I]-trazodone was significantly higher with the IN ME compared to the IV solution and IN solution at all sampled time points. Notably, the brain uptake for IN ME peaked at ~7% ID/g, while IN solution and IV solution had lower uptakes. The higher and faster uptake with IN ME suggests efficient delivery through the olfactory region. The highest brain uptake with the IN ME formulation, which was higher than that achieved with standard radiopharmaceuticals used for brain imaging such as [^99m^Tc]ECD and [^99m^Tc]HMPAO and higher than IN and IV solutions of [^131^I]trazodone. The elimination from the brain followed a decline over time but remained higher for IN ME throughout the observation period.

Fayez and co-workers developed a novel radiopharmaceutical, [^131^I]-rolapitant, formulated into spanlastic nanovesicles for brain imaging 71. The delivery device used was an IN-administration apparatus designed to deliver 15 μL of the solution through the nostrils. [^131^I]-rolapitant, acts as a selective high-affinity neurokinin-1 receptor antagonist, spanlastic nanovesicles significantly enhanced brain uptake and bioavailability compared to IV injection solutions. The spanlastic nanovesicles enhanced the delivery process by facilitating the penetration of the nasal epithelial cells and promoting uptake into brain regions. The surfactants, particularly Tween 80, used in the spanlastic nanovesicles formulation, acted as absorption enhancers, aiding in the transport across the BBB. These surfactants also reduced the clearance of NPs by the reticuloendothelial system and inhibited efflux systems like P-glycoprotein, further enhancing brain uptake. The study found that the maximum brain uptake was achieved at 2 h post-IN administration, with a notable value of ~7% ID/g. This high level of uptake was sustained for an extended period, with ~6% ID/g still present at 24 h post-administration. These findings highlight the efficiency of the IN-delivery system in ensuring prolonged drug retention in the brain. The study revealed significant brain uptake in normal mice, particularly when compared to IV injection. The distinctive distribution patterns showed that the IN administration of [^131^I]-rolapitant in spanlastic nanovesicles resulted in a 3-fold greater brain uptake than the IV solution. Furthermore, the brain/blood ratio, which reached a maximum of ~2 at 24 h post-administration, indicated a preferential accumulation of the radiolabelled drug in the brain, underscoring the potential of this delivery method for targeted brain imaging and therapy. The study also examined the elimination of tracer from the brain, revealing minimal clearance up to 24 h post-administration. This prolonged retention time is advantageous for imaging and therapeutic applications, as it allows for extended observation and effect of the radiolabelled drug in the brain. In conclusion, this approach highlights the potential of [^131^I]rolapitant spanlastic nanovesicles, as an efficient method for brain imaging and diagnosis of neurological disorders, particularly Alzheimer's disease and epilepsy.

Later work by Fayez *et al.* aimed to develop a selective SPECT radiotracer targeting the endothelin 1 receptor A in the brain by radiolabelling ferulic acid, an endothelin 1 receptor A antagonist, with [^131^I] 72. The [^131^I]-ferulic acid was formulated into nanosized polymeric micelles for IN administration to facilitate nose-to-brain delivery. The biodistribution of IN [^131^I]-ferulic acid polymeric micelles was compared to IN and IV [^131^I]-ferulic acid solutions to evaluate their efficiency in brain uptake. The mice biodistribution results demonstrated significantly higher brain uptake of [^131^I]-ferulic acid polymeric micelles at all time points compared to IV and IN [^131^I]-ferulic acid solutions. This enhanced brain uptake is attributed to the lipophilicity and nanoscale size of the polymeric micelles, which facilitate the direct transport of [^131^I]-ferulic acid to the brain via olfactory and trigeminal nerve pathways. In contrast, the IV [^131^I]-ferulic acid solution showed low brain uptake and high blood levels due to its poor lipophilicity and inability to cross the BBB. Similarly, the limited lipophilicity of the IN [^131^I]-ferulic acid solution resulted in poor nasal mucosal absorption, leading to low blood and brain uptake and high stomach uptake due to transfer through the nasopharynx to the stomach. The brain/blood ratio for IN ^131^I-ferulic acid polymeric micelles was consistently higher at all time points, especially at 30 min post-administration, indicating effective brain targeting. Specifically, the brain/blood ratio for IN [^131^I]-ferulic acid polymeric micelles was 1.09% ID/g at 30 min, compared to 0.18% ID/g for IN and 0.14% ID/g for IV [^131^I]-ferulic acid solutions. These findings suggest that IN [^131^I]-ferulic acid polymeric micelles could serve as a promising SPECT imaging tracer for the endothelin 1 receptor A in the brain. Furthermore, this study represents an initial step towards developing an endothelin 1 receptor A imaging agent, with further biological studies needed to validate these findings. Future research should explore the biodistribution of IN [^131^I]-ferulic acid polymeric micelles *in vivo* with induced head trauma to enhance endothelin 1 receptor A expression.

#### 2.1.4. Remarks on SPECT and gamma scintigraphy

SPECT and gamma scintigraphy have become pivotal in monitoring the efficiency and dynamics of INDD, especially with the high sensitivity of SPECT and the ability of gamma scintigraphy to precisely track drug transit and brain distribution. These modalities have significantly contributed to understanding the pharmacokinetics and biodistribution of various radiolabelled compounds following IN administration. A range of imaging agents for INDD have been investigated using SPECT, each with unique properties and half-lives catering to specific study requirements. ^99m^Tc is the predominant radionuclide used for SPECT, valued for its widespread availability, favourable photon energy for imaging, and short half-life. Vyas *et al.* (2005) assessed the nose-to-brain delivery of [^99m^Tc]-zolmitriptan in rats, exploring its efficacy in treating acute migraines. The study revealed that IN administration facilitated rapid brain uptake, surpassing other formulations and routes. The tracer's retention time in the brain was monitored for up to 8 h, with significant drug accumulation observed. Jogani *et al.* investigated [^99m^Tc]-labelled tacrine for Alzheimer's treatment, showing higher brain uptake and faster peak times compared to IV injection, suggesting enhanced bioavailability and reduced hepatotoxicity. Mustafa *et al.* (2012) assessed the IN administration of [^99m^Tc]-labelled ropinirole in rabbits, demonstrating superior brain localisation and rapid uptake through the olfactory pathway. Another study on chitosan NPs as a delivery vehicle for [^99m^Tc]-bromocriptine highlighted increased bioavailability in brain tissue and improved nose-to-brain delivery via intranasal administration. IN administration of [^99m^Tc]-carbamazepine showed higher brain uptake and prolonged retention compared to IV injection, demonstrating the benefits of mucoadhesive ME formulations. [^99m^Tc]-diazepam-loaded PLGA NPs demonstrated significant brain uptake and sustained presence, supporting the potential of this route for brain-targeted therapies. A study by Mandlik *et al.* on zolmitriptan-loaded nanostructured polymeric carriers showed enhanced brain uptake and retention, highlighting the effectiveness of nanocarriers for nose-to-brain transport. The elimination of [^99m^Tc]-labelled agents from the brain often involves systemic circulation and metabolic processes, with studies indicating a gradual washout of tracers from the brain over time. The clearance of these agents is critical in understanding the duration of their efficacy and potential side effects. Similar results were observed with other drugs like clonazepam, risperidone, and carbamazepine, indicating [^99m^Tc] ability to monitor nose-to-brain delivery efficiently.

[^86^Rb], [^201^TI], and [^54^Mn] have also been studied for their potential in brain imaging. A study on the olfactory transport route of [^86^Rb] and [^201^Tl] ions following IN administration demonstrated their significant accumulation in the olfactory bulb and subsequent spread to other brain regions. This indicated potential use in molecular imaging for conditions like anosmia and neurodegenerative diseases. Research on the transport of [^201^Tl] and [^54^Mn] ions in mice highlighted their reliance on olfactory nerve fibres for transport to the olfactory bulb, suggesting diagnostic applications for assessing olfactory nerve damage. Shiga *et al.* investigated the transport of [^201^Tl] to the brain using SPECT/MRI hybrid imaging, demonstrating peak uptake in the olfactory bulb and supporting its use in assessing olfactory disorders and potential infectious routes. Studies on [^201^Tl] migration in patients with olfactory impairments showed correlations between tracer migration, odour recognition thresholds, and olfactory bulb volume, providing insights into prognosis and recovery in idiopathic olfactory dysfunction. Tracers like [^86^Rb] and [^201^Tl] are gradually eliminated from the brain, with autoradiography revealing their transport behaviour and clearance patterns through olfactory pathways. These studies help delineate the pathways and mechanisms involved in the transport of these ions from the nasal cavity to the brain, providing a deeper understanding of their biodistribution and potential clinical applications.

Iodine-labelled compounds have shown promising results in non-invasive CNS imaging and targeted IN administration. Studies on [^125^I]-labelled Ct and its B subunit demonstrated distinct accumulation patterns in olfactory nerves/epithelium and olfactory bulbs. Research on [^125^I]-bFGF targeted Aβ plaques in a transgenic mouse model, highlighting the potential for labelling and imaging Aβ deposits. A study on [^125^I]-labelled IGF-1 demonstrated rapid entry into the CNS and significant concentrations in various brain regions, supporting its therapeutic potential for CNS disorders. [^125^I]-labelled VEGF showed extensive distribution within the CNS following IN administration, confirmed by gamma counting and autoradiography. [^123^I]-radiolabelled anti-Aβ peptides demonstrated effective brain targeting and retention, supporting their use for treating Alzheimer's disease. Iodine radioisotopes are cleared through lymphatic pathways and systemic circulation, with studies showing varied retention times and clearance patterns depending on the tracer and formulation. [^131^I]-labelled agents, such as [^131^I]-trazodone and [^131^I]-rolapitant, have demonstrated enhanced brain uptake and bioavailability with IN administration, suggesting effective targeting for brain imaging.

Gamma-emitting radiotracers offer unique insights into the nose-to-brain delivery mechanism, with variations in pharmacokinetics, uptake efficiency, and biodistribution. Together, the studies highlight the potential of IN administration as an alternative route for drug delivery to the brain, especially for therapeutic interventions in neurology and psychiatry. The ability of SPECT and gamma scintigraphy to quantify drug distribution precisely and to differentiate between drugs, metabolites, and other biological molecules has been crucial in these advancements. However, the field faces challenges such as optimising the balance between drug formulation, imaging resolution, and safety considerations, particularly concerning the toxicity of long-lived radionuclides. Continued research in this domain is essential to further harness what the imaging modalities can offer clinically and to develop more effective treatments for CNS disorders.

### 2.2. Positron emission tomography

PET stands at the forefront of molecular imaging techniques, offering unparalleled insights into metabolic activities at a cellular or molecular scale. This non-invasive imaging modality harnesses the power of radioisotopes to trace physiological processes in real-time tracking of radiolabelled drugs from nose-to-brain, providing invaluable data in both clinical and research settings. By using specific positron-emitting radionuclides attached to drugs, researchers can observe the direct transport pathways from the nasal cavity to the brain. The high sensitivity of PET allows for detailed visualisation of drug uptake and metabolism in brain tissues. PET is frequently combined with CT or MRI to provide a more comprehensive diagnostic tool that merges the strengths of each imaging modality. This combination allows for the simultaneous visualisation of both anatomical structures and biological functions within the body 73.

PET radioligands, ranging from small molecules to large biomolecules, serve as vehicles, transporting the radioactive label to the biological target sites. The radioactive labels are chosen based on their physical properties, primarily their half-lives (ranging from minutes to hours and to days), which directly influence the temporal imaging window and the resolution of the acquired images, as well as the required dosage 74.

The exploration of IN administration for brain imaging with PET introduces a strategic advantage by potentially circumventing the BBB. This pathway is particularly beneficial for the deployment of tracers that are substantially metabolised during first-pass hepatic metabolism, as well as those that might affect cerebral hemodynamic or vascular characteristics. Additionally, it allows for the administration of large molecular weight peptides, which are generally impeded by the BBB.

In this section, we survey the spectrum of positron emitting radioisotopes reported in the literature, each serving specific roles in PET imaging for IN administration. The commonly used [^18^F] (fluorine-18) stands out with its almost ideal imaging properties, providing a balanced half-life of 109.8 min, and offering high-resolution images with a relatively short positron range (i.e., distance travelled by a positron before annihilation with an electron). This makes it particularly suitable for high-precision studies over a span of several hours 75. On the shorter end of the half-life spectrum, [^11^C] (carbon-11), with a half-life of 20.4 min, necessitates rapid synthesis and imaging procedures but offers the advantage of studying rapid metabolic processes, as well as minimising radiation exposure. [^11^C] is often incorporated into biologically relevant molecules, allowing real-time tracking of molecular activity 76. Radiometals, such as [^64^Cu] (copper-64) with a 12.8 h half-life, introduce versatility to PET imaging, finding use in both diagnostic and therapeutic applications when chelated to various biomolecules 77. [^68^Ga] (Gallium-68)-based peptides are gaining recognition as a novel class of radiopharmaceuticals due to their rapid blood clearance and quick target localisation. The short physical half-life of [^68^Ga], which is ~68 min, allows for better dosimetry and the possibility of repeat imaging, making these agents highly suitable for clinical applications 78. [^89^Zr] (zirconium-89), a radiometal with a 78.4 h half-life, facilitates long-term tracking of biological events and has been shown to enhance diagnostic accuracy 79. Manganese isotopes ([^52^Mn], [^51^Mn], and [^54^Mn]) present unique imaging opportunities, ranging from short-term studies with [^51^Mn] (46.2 min) to prolonged tracking with [^52^Mn] (5.6 days) and [^54^Mn] (312.2 days) 80. Lastly, provides unique functionalities for specialised applications with a remarkably long half-life of approximately 12.3 years, [^3^H] emits low-energy beta particles and limited *in vivo* experiments 81.

The selection of isotopes is critical due to the variable positron ranges associated with different isotopes, which can significantly affect the spatial resolution of the images. Longer positron ranges can lead to image blurring, effectively reducing the resolution and degrading the quality of the diagnostic information. Moreover, *in vivo* studies using isotopes with long half-lives, such as ^54^Mn, require careful planning to manage the risks associated with prolonged radioactivity.

#### 2.2.1. Fluorine-18 ([^18^F])

[^18^F] has been used to monitor the *in vivo* absorption, distribution, metabolism, and excretion of drugs labelled with this isotope. Radiotracers include [^18^F]-fluorodeoxyglucose ([^18^F]FDG), [^18^F]-2-deoxy-2-fluorosorbitol ([^18^F]-FDS), [^18^F]-fallypride, [^18^F]-fluoride and [^18^F]-fluorothymidine ([^18^F]FLT). Standard uptake value (SUV) maps are often generated, or tracer time-activity curves (TACs) can be collected from dynamic PET scans to quantitatively assess the administered dose. From these measurements the spatial distribution or local concentration of the tracer can be derived for an image pixel (provided an appropriate experiment is utilised) or for any organ or anatomical structure situated within the imaging field-of-view of the imaging study.

[^18^F]FDG is a widely used PET radiopharmaceutical enabling mapping of glucose metabolism, including the CNS due to BBB permeability 82. Using this tracer, studies have explored INDD and focused on deposition and clearance. Shingaki *et al.* study employed [^18^F]FDG PET imaging to explore the pharmacokinetics of INDD absorption in rats, focusing on hydroxypropyl cellulose viscosity (0% to 3% w/v) and administered volumes (5 to 25 µL) 83. The nasopalatine duct was closed to prevent leakage into the oral cavity, mimicking the human nasal anatomy where the duct is degenerated. Key findings include the clearance of approximately 50% of [^18^F]FDG from the nasal cavity within 30 min, primarily through swallowing, which then allowed for its systemic circulation and brain deposition. Higher hydroxypropyl cellulose viscosity enhanced tracer uptake without altering nasal membrane permeability. Quantitation of drug reaching the brain was measured through the absorption rate constant, which varied between 0.0055 and 0.0177 per minute depending on the volume and viscosity of the dosing solution. The fraction absorbed at 30 min significantly increased with higher viscosity solutions, ranging from ~5% for saline, up to ~28% for 3% hydroxypropyl cellulose solutions. The study modelled pharmacokinetic parameters, showing that increased viscosity improved the absorption rate constant and reduced the mucociliary clearance rate constant, independent of dosing volume. PET imaging revealed time-dependent and region-specific changes in [^18^F]FDG permeability within the nasal cavity, correlating with the unique anatomical and physiological characteristics of its distinct regions: the nasal vestibule, atrium, respiratory area, olfactory region, and nasopharynx. This variability is influenced by blood flow, mucus secretion, ciliary beating, and the presence of various cell types, which affect drug absorption and permeability 84, 85. Permeability was notably higher in the anterior regions, underscoring the importance of regional differences in nasal mucosa for effective drug delivery. The study suggests that *in vivo* PET studies can offer more accurate pharmacokinetic parameters than *in vitro* studies, highlighting PET imaging's potential to enhance nasal dosage formulation development for systemic or CNS delivery, thereby improving INDD success rates by circumventing the first-pass effect. Additionally, highlights the capability of PET imaging in enhancing the understanding and development of nasal formulations for human use, highlighting the technique's ability to quantitatively analyse drug absorption and facilitate the prediction of pharmacokinetic parameters, thus providing a robust tool for future research and development 83.

**Figure 5:**
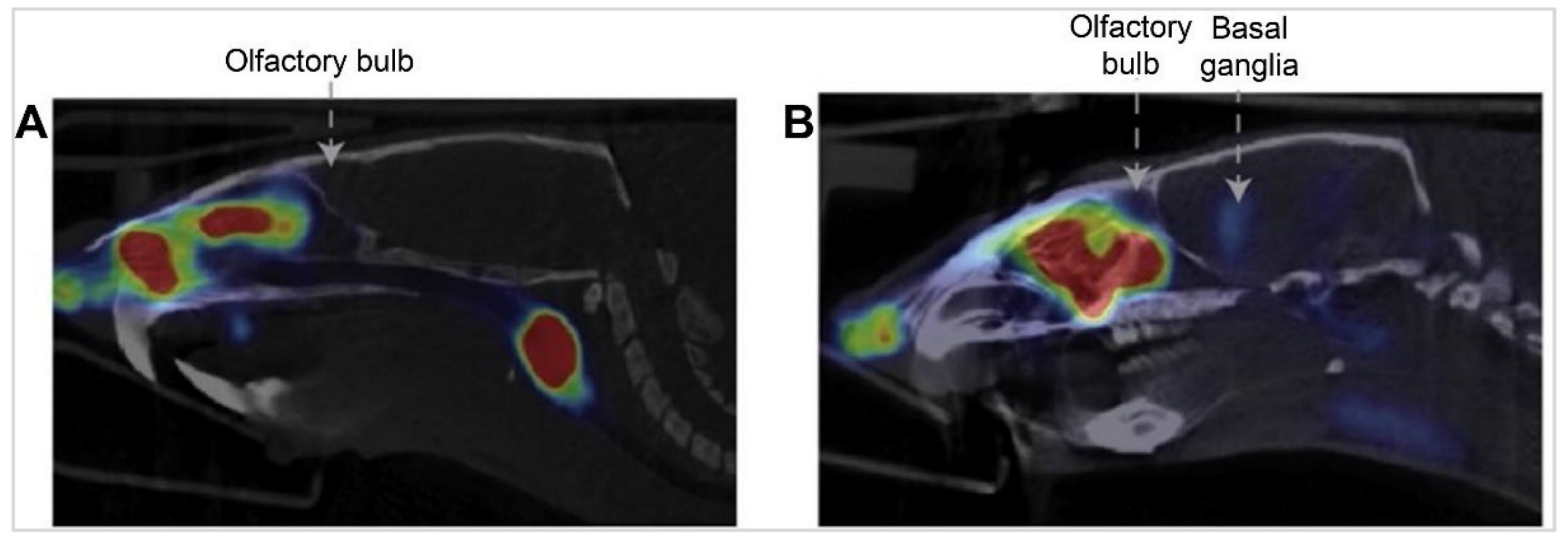
Presents lateral views of the brain following IN administration of (A) [^18^F]FDG and (B) [^18^F]fallypride. In both images, arrows point to the olfactory region. Additionally, the basal ganglia are indicated in the image (B) of [^18^F]fallypride. Adapted with permission from 86, copyright 2018 Elsevier.

Singh *et al.* performed a pharmacokinetics study for BBB penetrant tracers using [^18^F]FDG and [^18^F]fallypride, comparing IN and IV injection in rats 86. The delivery device used for IN administration was an insulin syringe. Each administration involved a total volume of 40 µL, with 20 µL per nostril. A significant difference between IV and IN administration in terms of brain uptake was absent, except for the olfactory bulb having a significantly higher uptake with IN administration (see Figure 5). For [^18^F]FDG, imaging was initiated approximately 33 min after IN administration, which was identified as the optimal time point for image resolution. For [^18^F]fallypride, imaging commenced around 10 min post-administration due to the necessary preparation and positioning time. The team reported that the absolute uptake of radiotracers in the study indicates that, for [^18^F]FDG, the concentration in the plasma was significantly lower when administered intranasally compared to IV injection. In contrast, [^18^F]fallypride, showed no significant difference in plasma concentration between the IN administration and IV injection routes, suggesting rapid absorption into the peripheral circulation for [^18^F]fallypride but not for [^18^F]FDG when IN administration. The retention time varied between the two tracers. For [^18^F]FDG, a significant proportion remained in the nasal cavity throughout the 45 min scan duration, with only about 30% reduction in radioactivity. Conversely, [^18^F]fallypride demonstrated an 80% reduction over a 60 min scan. This suggests that the overall brain uptake (excluding the olfactory bulb) from IN administration of [^18^F]fallypride is almost entirely supplied from peripheral circulation rather than directly from the nasal cavity. This observation is important, especially when weighed against the potential risks associated with IN administration, such as the high levels of cumulative skin radiation exposure and challenges related to non-specific signals. When tracers such as [^18^F]FDG and [^18^F]fallypride stay in the nasal cavity for an hour, potentially making this method risky due to the low amount of tracer reaching the brain and affecting image quality. Furthermore, this difference likely stems from [^18^F]FDG's tendency to become metabolically trapped within nasal cavity cells, leading to lower blood concentrations when IN-administered 87. On the other hand, [^18^F]fallypride does not get trapped in cells, allowing for quick absorption into the bloodstream and similar uptake rates between IN administration and IV injection. These findings indicate that the time a molecule spends within cells could significantly influence its nose-to-brain delivery. The authors suggested that further research is needed to explore how this mechanism affects the IN administration of other molecules, particularly those typically unable to cross the BBB 86.

[^18^F]FLT is known to have limited permeability/transport through the BBB, so brain delivery in rodents via nose-to-brain was studied 88. The study compared [^18^F]FDG and [^18^F]FLT for the purpose of identifying direct nose-to-brain pathways, and distribution after IN administration of 20-25 µL using a Hamilton syringe. A significant portion of the [^18^F]FLT dose was cleared through the oropharynx within a few minutes, and continued at a slower rate for 20 min 88. The residence time of [^18^F]FLT in the nasal cavity ranged from 15-30 min; however, a residual dose could still be measured in the nasal cavity 60 min after IN administration. [^18^F]FLT showed notably higher brain concentrations, particularly in the olfactory bulb, following IN administration compared to IV injection. This enhancement is immediate but diminishes within 30-45 min due to rapid clearance. [^18^F]FLT concentration increased in both the olfactory bulb and the brain over time until plateauing 25-30 min post-administration. At that time, [^18^F]FLT concentrations in the brain equalled almost one-third of that in the olfactory bulb. Conversely, [^18^F]FDG remained detectable for extended periods due to phosphorylation in the brain and nasal tissues, which hindered its clearance. The early clearance trend was comparable for both [^18^F]FDG and [^18^F]FLT, with half-lives of 2.53 and 3.36 min, respectively. However, during the latter, slower clearance phase, [^18^F]FLT cleared far more rapidly than [^18^F]FDG with half-lives 32.1 versus 85.2 min, respectively). Supplementary *ex vivo* tissue analyses confirmed the imaging results, supporting the observed distribution patterns of the tracers. Pharmacoimaging methods founded on mathematical models serve as a crucial tool in studying these processes; this can further be explored for IN administration studies through advancements in measurement precision and wider availability of suitable PET-labelled tracers, and enhancements in the development of delivery systems that promote targeting to distinct regions in the nasal cavity and prolong residence time.

[^18^F]FLT, a substrate of both equilibrative (ENT1,2) and concentrative (CNT1-3) nucleoside transporters, has also been used to investigate the role of nucleoside transporters in the nasal mucosa in INDD to the brain 89. Dynamic PET/CT scans were used to observe the distribution of [^18^F]FLT in rats up to 40 min, using both IN administration and IV injection. The effects of NBMPR (nitrobenzylmercaptopurine riboside), an ENT1 selective inhibitor, on [^18^F]FLT distribution was also explored. Results indicated that [^18^F]FLT concentrations were again higher in the olfactory bulb after IN administration compared to the rest of the brain. [^18^F]FLT having limited permeability across the BBB, resulted in minimal brain exposure post-IV injection 90. Retention studies showed rapid FLT clearance from the olfactory bulb within the first 20 min, followed by a slower clearance phase over the next 20 min. This pattern shows while initial uptake was fast, the sustained presence of FLT in the brain allowed for prolonged observation. Administering NBMPR intraperitoneally led to higher FLT exposure in both the olfactory bulb and the rest of the brain following IN administration and IV injection, corroborated by the *ex vivo* data. This indicates that NBMPR administration slowed [^18^F]FLT clearance from the brain. In contrast, co-administration of [^18^F]FLT and NBMPR decreased olfactory bulb exposure but increased brain exposure, suggesting ENT1 plays a role in the nose-to-brain transport pathways 89. In another study using the FLT with low beta energy particle, Carlisle *et al.* evaluated the effectiveness of delivering [^3^H]FLT to brain tissue via IN administration and IV injection routes in a rat model 91. They administered [^3^H]FLT to adult rats for both cases, then measured the concentrations of [^3^H]FLT in 16 different brain regions, as well as in the blood and non-target organs, at different timepoints post-administration. The concentrations of [^3^H]FLT in the olfactory bulb were consistently higher in the IN administration group than in the IV injection group. However, significant differences between the two groups were not found for all other brain regions. The brain/blood ratio values followed a similar pattern. Further analysis to reveal the efficiency of IN administration CNS radioisotope targeting indicated a more effective penetration into the olfactory bulb, spinal cord, and hippocampus with IN administration. Notably, non-target tissues such as the heart, lungs, adipose tissue, and skeletal muscle showed significantly higher concentrations of the radioisotope in both the 5- and 60-min trials for both IN administration and IV injection, compared to all brain regions. Additionally, high concentrations of the tracer in the heart and lungs highlight the importance of active/preferential targeting to the CNS. However, the use of [^3^H] presents significant limitations due to its low beta energy, impacting detection sensitivity, and its long half-life, raising safety concerns for *in vivo* studies 92.

An insightful study utilising PET/MRI technique investigated the role of the glymphatic system, crucial for brain waste clearance, using [^18^F]fluoride and [^18^F]FDS 93. The study revealed distinct clearance patterns for IN administration and IV injection. While [^18^F]fluoride showed high skull accumulation with both IN administration and IV injection, which was notably absent in [^18^F]FDS, effectively reducing spillover into brain tissue and enhancing the sensitivity for whole-brain analysis. Notably, [^18^F]fluoride after IN administration exhibited a 58% reduction in signal, contrasting with a 37% increase following IV injection. [^18^F]FDS demonstrated clear brain uptake and clearance with IN administration, showing a remarkable 110% change in PET signal after 105 min, whereas IV injection only showed a decline from baseline. These findings suggest that IN administration might be more effective for studies focusing on glymphatic efficiency, potentially guiding future research towards optimising tracer selection and administration routes to better understand and mitigate cognitive decline in aging and dementia.

An [^18^F]-labelled tracer, [^18^F]AlF-NODA-dLVT, has been developed to investigate the direct nose-to-brain transport of oxytocin, a hormone vital for childbirth and breastfeeding 94. This novel PET imaging agent, which incorporates a NODA chelator with the radioisotope [^18^F]aluminium and the oxytocin analogue dLVT, is designed to enhance both stability and selectivity. The tracer is specifically tailored for studying the direct nose-to-brain delivery of oxytocin-based therapies, enabling non-invasive monitoring of their uptake into the brain. Researchers observed that IN administration slightly increased tracer uptake in the brain parenchyma compared to IV injection, with uptake from IN administration at 0.11% ID/g versus 0.05% ID/g from IV injection. Although there was enhanced tracer uptake in the olfactory bulb following IN administration, indicative of potential nose-to-brain transport, the tracer did not significantly reach deeper brain regions within a 90 min PET imaging timeframe. Furthermore, the pattern of distribution post-IN administration did not prove to be substantially more effective than IV injection for delivering oxytocin-based tracers into the brain. The presence of radioactivity in the cerebellum suggests potential peptide degradation post-absorption, underscoring the need for improvements in the stability and selectivity of oxytocin agonists to boost clinical efficacy. While the findings indicate that IN administration might influence behavioural and neural responses, they also highlight the necessity for further enhancements in tracer stability and selectivity to improve clinical outcomes. The results, while promising, provide limited insight into the efficacy of [^18^F]-based radiotracers in exclusively mapping the nose-to-brain delivery pathway of oxytocin.

Restoring insulin, a peptide hormone produced by pancreatic β cells, levels in the CNS has gathered interest as a potential treatment for Alzheimer's disease. Patients with Alzheimer's disease exhibit elevated plasma insulin levels and reduced CSF insulin levels, with this pattern becoming more pronounced as their clinical impairment worsens 95. Nose-to-brain delivery of insulin has attracted attention as a non-invasive pathway to enhance CSF insulin levels without affecting peripheral insulin levels. Studies in rodents indicate that molecules delivered via nose-to-brain travel along the olfactory and trigeminal pathways to reach the CNS. A recent study by Smith *et al.* employed PET to monitor the uptake of [^18^F]-labelled insulin ([^18^F]-insulin) from nose-to-brain uptake in non-human primates 96. Three nose-to-brain delivery methods for [^18^F]-insulin were evaluated: aerosol delivery via tubing in rhesus macaques, aerosol delivery via a preplaced catheter in rhesus macaques, and solution delivery via a preplaced catheter in cynomolgus macaques. Post-administration, dynamic PET imaging for 120 min was performed to quantify the delivery efficiency of nose-to-brain. The area under the time-activity curve (TAC) was calculated for 46 brain regions of the cynomolgus macaque to assess regional [^18^F]-insulin levels. The C_max_ of [^18^F]-insulin in the whole brain was observed in the first PET frame for two out of three experiments. This indicates that intranasally delivered insulin reaches the brain within 13 min of administration. The imaging revealed that liquid instillation of [^18^F]-insulin via catheter was more effective than aerosol methods for both delivery to the subject (~40% ID vs. ~10% for aerosol via tubing, 0.17% for aerosol via catheter) and subsequent brain delivery (0.34% ID vs. 0.0002% for aerosol via tubing, 0.05% for aerosol via catheter). [^18^F]-insulin was quickly transferred across the cribriform plate to the limbic and frontotemporal regions, which are key areas for emotional and memory processing. Limbic and frontotemporal cortical regions are crucial therapeutic targets for Alzheimer's disease patients. An earlier study classified Alzheimer's disease patients mainly into "temporoparietal" or "limbic-predominant" hypometabolic patterns 97. The half-life of [^18^F]-insulin was longer in olfactory nerve projection sites with high insulin receptor density compared to the entire brain. The accumulation and clearance rates of [^18^F]-insulin varied by region. A one-phase decay model showed that the half-lives for the nasal cavity, cribriform plate, and whole brain were approximately 30-, 20-, and 31 min, respectively. The model fits well for the nasal cavity and whole brain but less accurately for the cribriform plate. Differences in [^18^F]-insulin kinetics were visualised using normalised SUV TACs for various brain regions. Some regions, like the frontal-orbital gyrus and anterior cingulate cortex, had sustained activity but faster clearance rates. In contrast, regions like the amygdala and hippocampus cleared the tracer more slowly, with longer half-lives. The nucleus accumbens showed a unique oscillating pattern. In conclusion, the catheter-based liquid delivery method, combined with PET imaging, effectively tracked the nose-to-brain [^18^F]-insulin and is believed to be broadly applicable for evaluating other therapeutic agents 96. This approach can rapidly be applied in human studies to advance the clinical evaluation of nose-to-brain insulin as a treatment for Alzheimer's disease. More recently, a study aimed to develop a non-invasive delivery system for overcoming the BBB using the nose-to-brain route 98. PEGylated-PLGA NPs coated with chitosan were designed to facilitate nasal absorption and efficient brain transfer of insulin. Characterisation and *in vivo* studies, including biodistribution and PET imaging, indicated higher fluorescence in the brain, suggesting efficient NP delivery. These findings highlight the potential of PEGylated and chitosan-coated NPs for targeted insulin delivery to the brain, providing a promising platform for treating Alzheimer's disease by restoring memory signalling. Together, these findings from NP-based and [^18^F]-insulin studies propose innovative nasal delivery systems for insulin, providing a solid foundation for further clinical evaluation and potential treatment of Alzheimer's disease.

#### 2.2.2. Carbon-11 ([^11^C])

Zolmitriptan is a selective serotonin receptor agonist that readily crosses the BBB and is known for its high efficacy in the acute management of migraines 99. Yates *et al.* utilised an IN-spray device to administer [^11^C]-labelled zolmitriptan in a 100 μL solution on healthy volunteers to assess the distribution and pharmacokinetics of the drug using PET imaging 100. Immediately after administration, close to 100% ID was detected in the nasopharynx. This concentration declined over time, with 50% remaining at 20 min and 35% at 80 min post-administration. These results indicated rapid absorption and distribution of the drug from the nasal cavity. Radioactivity first appeared in the upper abdomen during the first 20 to 40 min post-dose. Minimal radioactivity was detected in the lungs, with only 0.2% at 20 min and 0.3% at 80 min post-dose. The brain showed low but detectable levels of radioactivity, with SUV ranging from 0.006 to 0.070, suggesting some central penetration. The mean brain concentration of radioactivity for all volunteers was SUV 0.020, equivalent to 0.54 ng/g. There was a strong correlation between brain SUV and blood SUV, with brain values being approximately 1/5^th^ of blood values, indicating that a fraction of the drug reached the brain. The brain retained low but detectable levels of radioactivity throughout the 1.5 h scanning period. While the study confirms central penetration of zolmitriptan with detectable levels of radioactivity in the brain, it recommends caution as some of the detected radioactivity might be attributable to metabolites. A high degree of correlation (correlation coefficient = 0.95) was noted between cerebral and blood uptakes, which was inconsistent with previous findings on zolmitriptan crossing the BBB 101, 102; where the cerebral values were approximately 0.2-fold of the corresponding blood values. Despite minimal levels in cerebral tissues, quantifiable radioactivity was consistently detected, indicating the central albeit residual penetration of [^11^C]zolmitriptan.

A separate study sought to elucidate the uptake and brain distribution of [^11^C]zolmitriptan following IN administration by a nasal spray in healthy volunteers, finding rapid absorption via the nasal mucosa, with detectable levels in brain tissue as early as 5 min post-administration 103. PET imaging revealed a fast and dose-dependent absorption of [^11^C]zolmitriptan into the brain. Notable levels of [^11^C]zolmitriptan were observed across all examined brain areas. The initial peak in uptake occurred at the end of the 5 min infusion, followed by a gradual decrease in SUV over the next hour. The highest uptake was observed in large blood vessels and areas without a BBB, such as the choroid plexus, as well as in grey matter regions, with the inferior temporal cortex having the highest uptake. Following IN administration, the concentrations in the CNS progressively increased, peaking at approximately 2 nM by the 0.5 h mark and 3.5 nM after 1 h. The mean concentration in the CNS at 5 min reached 0.5 nM quickly, surpassing the in vitro thresholds necessary to start agonistic effects on 5-HT_1B/1D_ receptors, a specific subtype of serotonin receptors. The authors concluded uptake into the brain was predominantly via systemic circulation, where it then traversed the BBB. Over time, [^11^C]zolmitriptan permeated all brain regions, with its concentration in the brain reaching nearly 20% of that in the plasma after 60 min. Despite this progressive uptake, the total level of uptake remained relatively low, consistently around 12% of the total administered activity. Additionally, after reaching its peak concentration, [^11^C]zolmitriptan showed a gradual decrease in concentration over time, as it washed out of brain tissue. The study also indicated that zolmitriptan is eliminated through hepatic biotransformation to three major metabolites, including 183C91, a potent 5-HT_1B/1D_ agonist. At 60 min post-administration, 46% of the [^11^C]zolmitriptan in plasma was intact tracer plus the active metabolite 183C91. The gradual decrease in SUV over the last hour of the PET scan reflected the washout of the tracer from the brain. The study underscored the efficacy of IN administration of ^11^C-zolmitriptan in rapidly delivering the drug to the brain, facilitating prompt pharmacological action, which is essential for the effective management of acute migraine symptoms. These pharmacokinetic properties support the use of [^11^C]zolmitriptan in situations where rapid onset of action is critical and highlight its suitability as a treatment option in the acute phase of migraine attacks.

Comparing the pharmacokinetics and applications of zolmitriptan labelled with different imaging agents highlights distinct differences and similarities across the studies. Experiments involving [^99m^Tc]-zolmitriptan formulations, such as MEs and mucoadhesive MEs demonstrated rapid and efficient brain uptake with higher DTE% and DTP% compared to other formulations 24. Particularly, the mucoadhesive MEs had superior brain retention and rapid onset of action, with a DTE% of 533 and a DTP% of 81, indicating effective nose-to-brain delivery. In contrast, the [^11^C]-labelled zolmitriptan administered to healthy volunteers had lower but detectable brain penetration, with mean brain concentrations peaking at 3.5 nM and brain uptake reaching nearly 20% of plasma levels 100, 103. In these human studies brain uptake was primarily through systemic circulation through the crossing of the BBB. The [^99m^Tc]-zolmitriptan nanocarriers also involved enhanced brain uptake and prolonged retention, with a brain/blood ratio 5-fold higher than IV injection, highlighting the potential of nanostructured carriers in facilitating efficient nose-to-brain transport 37. While both tracers confirmed zolmitriptan's potential for nose-to-brain delivery, the [^99m^Tc] formulations generally exhibited higher and prolonged brain uptake compared to the [^11^C] formulations, which had a stronger correlation with the systemic circulation. These differences emphasise the impact of the imaging agent, formulation, and administration method on the observed pharmacokinetic outcomes and the efficacy of zolmitriptan for targeted brain delivery.

**Figure 6:**
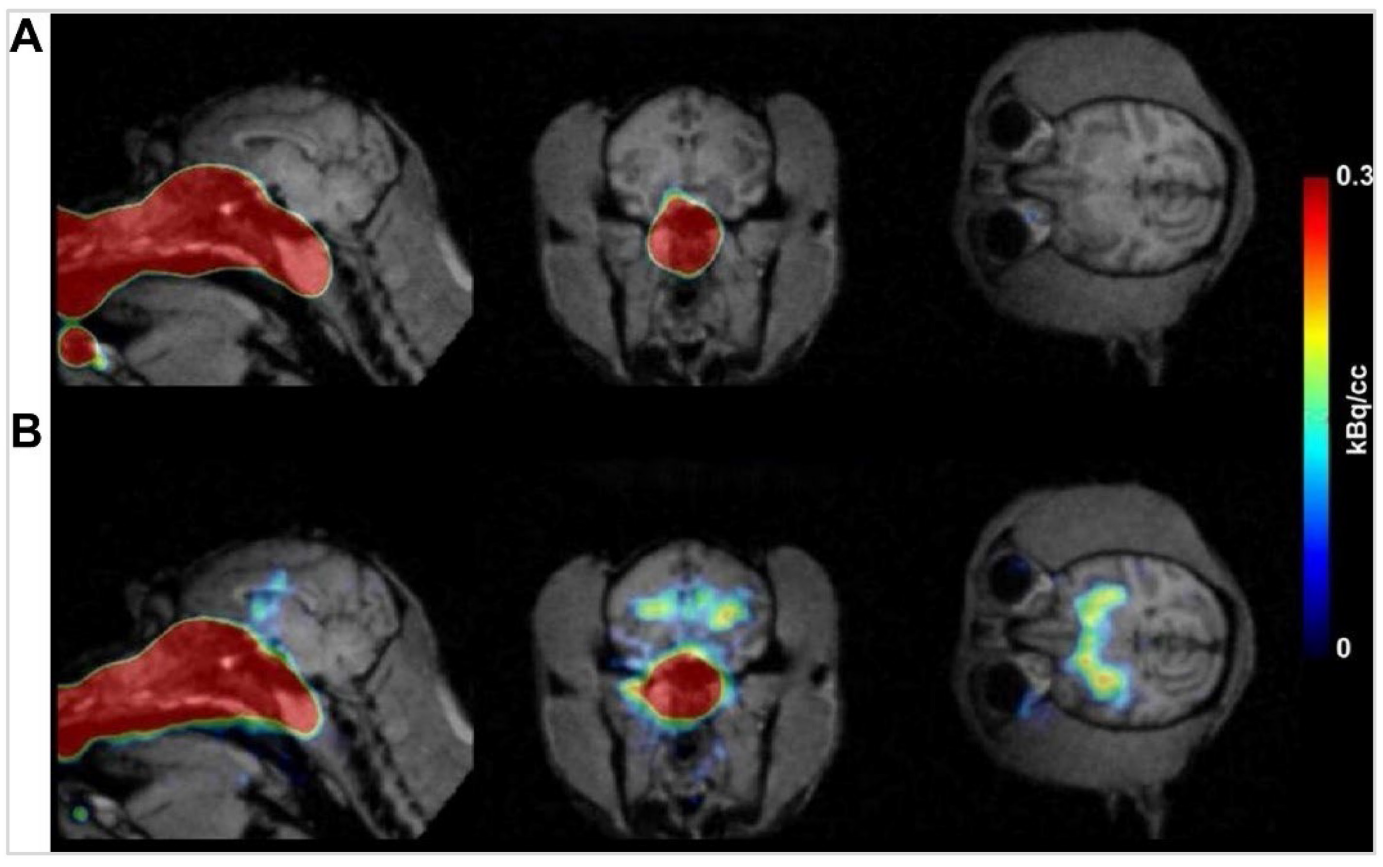
Brain PET/MRI images for rhesus macaque post IN administration (A) [^11^C]CH_3_-Orexin A tracer and (B) [^11^C]raclopride. (A) Images showing no detectable brain uptake in unaffected regions. (B) Images show uptake concentrated in the striatum, highlighting dopamine D2/D3 receptor binding. Adapted with permission from 104, copyright 2018 American Chemical Society.

[^11^C] was also used to radiolabel Orexin-A, a natural neuropeptide and potential antinarcoleptic medication that targets the Orexin receptor 1, to investigate its delivery mechanism into the brain upon IN administration in non-human primates and rodents using a nasal spray 104. The study employed a syringe-catheter device and the Impel POD administration device for rodent models, while an IN administration device developed by Impel NeuroPharma was used for non-human primates. The researchers employed [^11^C]CH_3_-Orexin A solution, a novel radiolabelled form of Orexin A, to track the peptide's brain distribution via PET imaging. The imaging revealed that following IN administration of [^11^C]CH_3_-Orexin A indicated the uptake was only detected in the olfactory bulb, parts of the frontal cortex, and also distally in the brain stem. These measured signal changes were attributed to partial volume effects. No uptake was reported in any other regions in the brain 90 min post-administration. The team demonstrated that such findings indicate a potential lack of nose-to-brain delivery pathway for [^11^C]CH_3_-Orexin A (see Figure 6). On the other hand, IN administration of ^11^C-raclopride solution, is a well-known radiotracer used in PET imaging to study dopamine D2/D3 receptors, which are abundant in the striatum of the brain, showed notable radioactive signal increase in various brain regions. A small but detectable amount of ^11^C-raclopride was observed in the striatum, a region with high D2 and D3 dopamine receptor binding. Blood analysis post-administration of [^11^C]raclopride suggested that its brain uptake could be attributed to its presence in the blood, aligning with the patterns seen in IV injection. However, the possibility of direct nose-to-brain transport of [^11^C]raclopride via IN administration remains open to debate, highlighting differences in the brain accessibility of small molecules versus peptides like Orexin A.

To further investigate the limited brain uptake of [^11^C]CH_3_-Orexin A that was observed in non-human primates, a similar study was performed in rodents, employing both PET imaging and *ex vivo* analysis with [^11^C]CH_3_-Orexin A, and [^125^I]Orexin A. After IN administration of [^11^C]CH_3_-Orexin A, PET imaging in rodents mirrored the results in the rhesus macaque, showing minimal brain uptake, particularly in areas not affected by partial volume effects. Subsequent *ex vivo* analysis of rodent brain sections also revealed very low radioactivity levels (signal-to-noise ratio < 3). This was attributed to both the negligible uptake of [^11^C]CH_3_-Orexin A in the brain and the rapid decay of the ^11^C isotope. The authors used [^125^I]Orexin A to address the issue of low radioactivity observed in *ex vivo* brain tissue after IN administration of [^11^C]CH_3_-Orexin A, further *ex vivo* brain distribution studies were conducted using [^125^I]Orexin A. The results showed similar patterns of distribution were observed for both IN administration and IV injection. However, IN administration resulted in a unilateral increase of radioactivity in the olfactory bulb ipsilateral to the administration site, averaged between 0.01 to 0.03% ID/cc, with the highest concentration reaching up to 0.07% ID/cc, suggesting a potential for enhanced local uptake. In contrast, IV injection showed uniform uptake across both olfactory bulbs. Overall, minimal brain uptake of Orexin A through IN administration using [^11^C]CH_3_-Orexin A was found, while ^125^I-Orexin A showcased a possibility for localised enhancement in uptake. Despite the minimal brain uptake of Orexin A through IN administration using [^11^C]CH_3_-Orexin A, the study proposed an alternative mechanism for the observed therapeutic effects of IN administration of Orexin A. This mechanism involves the activation of orexin receptor 1 in the olfactory sensory neurons, which could influence sleep-wake cycles through downstream signalling. The researchers advocate for future studies to explore this hypothesis by examining both brain uptake and therapeutic efficacy following IN administration and direct brain injections, particularly in animal models with reduced expression of Orexin A receptors in olfactory sensory neurons.

#### 2.2.3. Zirconium-89 ([^89^Zr])

In a study by Yu *et al.* (2016) on tumour tropism via PET/CT imaging, the migration of human neural stem cells (hNSCs) labelled with ^89^Zr in rats was measured after IN administration 105. ^89^Zr was used due to the long half-life that allows for extended observation periods, which is crucial for detailed monitoring of NSCs migration and distribution post-administration. This BBB circumventing approach demonstrates promising therapeutic potential, especially when used in conjunction with radiation and oncolytic virotherapy. Utilising a unique mesoporous NP-based radiolabelling technique with ^89^Zr, the study achieved high sensitivity in PET imaging, enabling precise tracing of small numbers of IN administration delivered stem cells. Comparative analysis revealed distinct primary migration routes for NSCs and mesenchymal stem cells (MSCs). While MSCs predominantly migrated toward glioma xenografts through the brain's caudal regions, NSCs tended to navigate through the rostral regions, with a significant presence in the olfactory bulbs. This differential migration pattern is hypothesised to be influenced by the chemotaxic cytokine receptor profiles of the respective stem cell types, responding variably to the diverse cues of the tissue microenvironment.

[^89^Zr] was used as the radionuclide to investigate the sensitivity of PET/CT imaging *in vitro* and to track the migration of hNSCs into the brain after IN administration in rats 106. The study proposed extracellular transport through the nasal cavity and potential intracellular transport mechanisms. The nasally administered hNSCs were foremost distributed in the nasal cavity, with limited transport to the brain. PET/CT scans were performed at various time points post-administration: 10 min, 35 min, 1 h, 4 h, 24 h, 72 h, and 168 h. The *in vitro* sensitivity of PET/CT imaging for detecting as few as 1000 [^89^Zr]-labelled cells was established. Upon IN administration, *in vivo* PET/CT imaging of [^89^Zr]hNSCs demonstrated high uptake in the nasal cavity and olfactory bulb within the initial 4 h period. Minor uptake was observed in the pituitary and pons, while the frontal lobe, parietal lobe, striatum, hippocampus, and ventral tegmental area showed relatively low signals, primarily below the detection limit. Retention times showed that radioactive uptake values in the nasal cavity and olfactory bulb gradually decreased over time, with no significant signals detected after 4 h post-administration. To complement PET findings, the study employed BLI using a human norepinephrine transporter-luciferase plasmid NSCs for both *in vitro* and *in vivo* experiments. The *in vitro* sensitivity was tested by seeding different numbers of human norepinephrine transporter-luciferase plasmid NSCs in 24-well plates and performing fluorescence scanning. For *in vivo* experiments, different amounts of human norepinephrine transporter-luciferase plasmid NSCs were administered intrastriatally and nasally to rats and C57BL/6 mice. BLI captured fluorescence signals post-administration, confirming the localisation and distribution patterns observed in FLI. The *ex vivo* analysis performed at 168 h post-administration confirmed the absence of radioactive signals, indicating that the radioactive substance did not substantially enter the brain, or was below the detection limit of the instrument. This underscores the predominant distribution of [^89^Zr]hNSCs in the nasal cavity, and their limited detectability in the brain following IN administration in rats. In conclusion, the IN administration of hNSCs in this study revealed limited migration to the brain, with the majority of cells remaining in the nasal cavity and olfactory bulb. PET, FLI, and BLI imaging demonstrated low sensitivity for detecting hNSCs in the brain, while droplet digital polymerase chain reaction and immunohistochemistry validated the biodistribution and presence of hNSCs in different tissues. Droplet digital polymerase chain reaction demonstrated higher sensitivity and accuracy compared to BLI and PET/CT, allowing for the detection of as few as 100-200 cells in tissue samples. This method provided a robust pharmacokinetic profile for tracking hNSCs, complementing the imaging findings and supporting the limited brain uptake observed through PET/CT.

In 2021, Veronesi *et al.* investigated the feasibility of using multimodal imaging for IN administration of polymeric micellar NPs radiolabelled with [^89^Zr] in rats using a nasal tubing delivery system 107. The uptake for [^89^Zr]PLA (polylactic acid)-polyethylene glycol (PEG)-DSPE (Distearoylphosphatidylethanolamine) with diameter size of 100 nm in the olfactory bulb, brainstem, and forebrain was significantly higher upon IN administration compared to IV injection at both 1 and 2 h post-administration. The mechanism of NP delivery via the IN route involves transcellular and intercellular pathways, allowing access to the perineural and perivascular fluid channels surrounding the olfactory and trigeminal nerves. The rapid detection of NPs in the brain within 1 h suggests that bulk convection flow through these perineural and perivascular routes significantly contributes to the observed delivery efficiency. This increase in brain activity after IN administration aligns with earlier findings, such as those reported by Kozlovskaya *et al.* in 2014 108. Notably, higher levels of activity were observed in the olfactory region and brainstem than in the forebrain post-IN administration, suggesting that the NPs entered the brain through the olfactory and trigeminal pathways, as these areas are more proximal entry points compared to the more distal forebrain. *Ex vivo* gamma imaging confirmed that activity in the olfactory bulb following IN delivery was 35.1- and 28.6-fold higher than IV injection at 1 and 2 h, respectively. Brainstem activity was 28.9- and 29.6-fold higher, and forebrain activity was 11.2- and 7.8-fold higher for IN delivery compared to IV injection at the same time points. Here, NPs might have been cleared through CSF spaces, with potential contributions from the blood pool. This aspect remains an area for further investigation to fully understand the pharmacokinetics of intranasally delivered NPs. Although PET results showed increased brain activity following IN administration compared to IV injection, it was difficult to distinguish between the activity levels at the 1 and 2 h post-administration due to PET's lower sensitivity compared to gamma counting. The inability to completely isolate the brain from the nasal cavity and oropharynx *in vivo* studies suggests that some high activity observed in the olfactory bulb and brainstem might partially result from activity in adjacent tissues. This might also explain the lower values seen in the olfactory bulb relative to the brainstem on PET, though gamma counting showed higher values in the olfactory bulb. Furthermore, the study employed autoradiography for *ex vivo* validation of PET/CT imaging findings, which demonstrated localised activity in dissected brain sections. The authors acknowledged the necessity of future studies employing transmission electron microscopy to confirm NP entry into brain tissue and provide histological evidence supporting the imaging results. This observation emphasises the potential of IN administration as a direct and effective method for delivering therapeutic agents to the brain.

#### 2.2.4. Gallium-68 ([^68^Ga])

A PET imaging probe capable of monitoring insulin *in vivo* would significantly benefit clinical interventions targeting insulin resistance. A study by Gollapelli and the team aimed presented the *in vitro* serum stability and autoradiography results of [^68^Ga]-NOTA labelled with insulin in rodent and non-human primate models of Alzheimer's disease pathology 109. The [^68^Ga]-NOTA-insulin remarkably maintained over 97% stability in human serum for up to 5 h post-production. Autoradiography results found a significantly lower brain uptake of the radiotracer in transgenic mice compared to age-matched wild-type mice. Additionally, vervets with low CSF Aβ_42_ levels, indicative of Alzheimer's disease, exhibited reduced uptake in the hippocampus and hypothalamus compared to control vervets with high CSF Aβ_42_ levels. Notably, there was no significant difference in uptake observed in the frontal cortex and cerebellum. Self-blockade experiments further demonstrated reduced uptake compared to baseline, confirming the specificity of the radiotracer. Overall, the preliminary autoradiography studies in both rodent and non-human primate models of Alzheimer's disease points to lower uptake of [^68^Ga]-NOTA-insulin in Alzheimer's disease brains compared to controls. Furthermore, radiotracer uptake in the hippocampus and hypothalamus was inversely correlated with Aβ density in vervet brain tissues, as determined by immunohistochemical analysis. The team's ongoing experiments focus on *in vivo* IN administration and IV injection PET imaging of [^68^Ga]-NOTA-insulin in non-human primate models of Alzheimer's disease, the determination of metabolic plasma-blood parameters, and the correlation of radiotracer uptake with Aβ_42_ and tau levels. Such studies aim to provide additional insights into the potential of [^68^Ga]-NOTA-insulin as a diagnostic tool for Alzheimer's disease and its underlying mechanisms of insulin resistance.

#### 2.2.5. Manganese ([^51^Mn], [^52^Mn], [^54^Mn])

[^51^Mn] and [^52^Mn] radioisotopes have been studied as experimental tools preclinically, in both rats and macaque monkeys 110. Mn radioactive markers provide analogous data to Mn^2+^ in Mn-enhanced MRI, even at reduced concentrations, by identifying accumulation in different tissues after systemic delivery. This represents approximately a 2000-fold reduction (~100 mM for Mn^2+^ in MRI vs. ~0.05 mM for PET). Tjalve and colleagues used a Hamilton syringe to deliver isotopes 111. For both [^54^Mn] and cadmium-109, a volume of 10 µL of isotope solution was administered intranasally into the right nostril of the rats. The delivery pathway for both Mn and cadmium was through the IN route, specifically targeting the olfactory mucosa. The primary mechanism for Mn delivery to the brain involved its transport via primary olfactory neurons to the olfactory bulbs. From there, Mn migrated through secondary and tertiary olfactory pathways, eventually reaching various brain regions and the spinal cord. In contrast, cadmium was taken up into the anterior parts of the olfactory bulbs but could not pass the synapses to secondary neurons. This limitation in crossing synapses restricted cadmium's distribution within the brain. Mn showed a rapid uptake, with significant accumulation in the olfactory bulbs observed within one-day post-administration. Over the subsequent days and weeks, Mn spread to additional brain regions, including the basal forebrain, hypothalamus, thalamus, habenular complex, hippocampus, and cerebral cortex, and even reached the spinal cord. The retention time for Mn was substantial, with detectable levels persisting in these regions for up to 12 weeks. Cadmium, on the other hand, demonstrated a more restricted uptake. It was primarily confined to the olfactory bulbs at all observed intervals, with minimal spread to other brain regions. These findings indicate that cadmium's retention time in the brain was mainly limited to the olfactory bulbs, with little to no distribution beyond this area. The elimination of Mn from the brain occurred gradually, with a noticeable decrease in concentration over time. Despite this decline, Mn levels in the brain remained higher than in the liver and kidneys at later time points, a hallmark of a slow washout process. Cadmium's elimination pattern was different; it was primarily retained in the olfactory bulbs with minimal spread to other brain regions, i.e., limited washout from the brain. Mn exhibited broad distribution throughout the brain, especially in cell-rich areas such as the basal forebrain and hypothalamus. The autoradiograms showed Mn's progressive movement from the olfactory bulbs to other brain regions, confirming the bypassing of the BBB via olfactory pathways. Cadmium's autoradiography confirmed its high retention in the olfactory bulbs with limited distribution beyond this region, illustrating its inability to pass the synapses to secondary olfactory neurons. The olfactory system appeared to serve as a route for Mn to reach the brain by uptake by olfactory neurons and its subsequent transfer through secondary and tertiary olfactory pathways to other regions. The limitation of using [^54^Mn] is the need for brain dissection due to its prolonged presence, which can complicate non-invasive longitudinal studies and raise safety concerns regarding radiation exposure over extended periods 110.

Mn-enhanced MRI in rodents indicates that it takes around 24 h for Mn to distribute throughout the brain after an IV injection 112, 113. As a result, [^51^Mn], with its short half-life, is unsuitable for studying Mn uptake in the brain. This limitation was evident in experiments with both rats and monkeys, where virtually no tracer activity was observed in the initial two hours 110. In contrast, [^52^Mn], with its longer half-life, is effective for identifying brain structures. It can be employed to image brain anatomy and functions, as well as to investigate pathophysiological conditions. When [^52^Mn]Mn^2+^ in rats, it was noted that the tracer not only accumulated in the body but was also detected in the brain and head regions, with significant tracer accumulation in the nasal turbinates, pituitary, olfactory bulb, hippocampus, and salivary glands. However, the low resolution of PET imaging combined with the small size of the rat made it difficult to precisely differentiate between brain areas.

Mn-enhanced MRI is used to track the olfactory pathways in the monkey brain post-IN administration enables mapping of neural pathways starting from the nostril and reaching as far as the amygdala over a period of days in ways comparable to Mn-enhanced MRI 110. Using [^52^Mn]Mn^2+^ solution as PET radiotracer. Initial PET scans taken on day 0 show the tracer observed in the nasal cavity without any detectable uptake in the brain. By day 1, it was observable in the olfactory bulb, olfactory tract, and olfactory nucleus. By day 3, the tracer reached the piriform cortex, and by day 4, it extended to the amygdala. The accumulation of the tracer intensified in the piriform cortex and amygdala by days 6 and 7, and by then, it was also detected in the frontal cortex. The tracer's migration aligns with the olfactory pathway, which includes comparisons to atlas slices. The route mapped includes progression through the olfactory bulb, olfactory tract, olfactory nucleus, piriform cortex, amygdala, and frontal cortex. During the same PET scanning session, whole-body PET imaging was conducted on each monkey to monitor the distribution of the radiotracer throughout the body. Immediately after the administration of [^52^Mn]Mn^2+^, the tracer was primarily found in the nasal area. However, most of the ingested Mn has been exerted from the body through the digestive tract and bladder, with only trace amounts remaining in the liver and kidneys. The elimination process continues, and by the seventh day, only minimal traces of Mn are detectable in the liver and kidneys. The study underscored the capability of high-sensitivity PET imaging to map the distribution of Mn radiotracers in both the brain and body, achieving results comparable to manganese-enhanced MRI studies. The co-registration of PET images with MRI provided enhanced resolution and anatomical localisation, facilitating a detailed understanding of tracer distribution and neuronal pathways. The findings are supported by systematic comparisons with MEMRI studies, demonstrating that even at lower concentrations, Mn uptake and transport mechanisms remain consistent.

#### 2.2.6. Remarks on PET

PET imaging has shown significant promise in the study of IN administration of neuroimaging agents. The use of various radioisotopes, particularly [^18^F] and [^11^C], has provided a deeper understanding of the nose-to-brain delivery pathway. Tracers such as [^18^F]FDG and [^18^F]FLT have been instrumental in assessing the direct transport and metabolic processing of imaging agents in the brain. For instance, [^18^F]FDG studies have revealed significant insights into the IN administration-mediated delivery, highlighting the rapid clearance from the nasal cavity and the contribution of systemic circulation in brain delivery, illustrating a mixed nose-to-brain and systemic pathway. Similarly, [^18^F]FLT, with its limited BBB permeability, offers a unique perspective on the nose-to-brain pathway, emphasising the immediate but transient enhancement in brain distribution following IN administration. In contrast, the use of [^11^C] labelled zolmitriptan and orexin-A has shed light on the distribution patterns and potential limitations of IN administration. For example, [^11^C]zolmitriptan studies found minimal yet detectable brain uptake, suggesting a predominant systemic route of brain delivery post-IN administration. The application of [^11^C]Orexin A resulted in limited uptake in the brain, indicating potential challenges in achieving effective nose-to-brain delivery for certain molecules. The use of [^11^C] in these studies highlighted both advantages and limitations. While [^11^C] provides high-resolution PET imaging and minimal long-term radiation exposure due to its short half-life, these same properties pose logistical challenges and limit its effectiveness for capturing slower biological processes or assessing deeper brain penetration. These findings indicate that while [^11^C] is valuable for certain scenarios, alternative isotopes might be necessary for a more comprehensive assessment of INDD mechanisms, particularly for targeting deeper brain structures. Additionally, the application of radiometals like [^89^Zr], [^68^Ga] and [^64^Cu] has expanded the scope of PET imaging in IN administration studies. The tracking of ^89^Zr-labeled hNSCs, [^68^Ga]-NOTA labelled insulin, and [^64^Cu]AuNCs (data provided in this section 2.5.2) has provided valuable data on the migration and biodistribution patterns of these agents, further contributing to our understanding of IN administration's potential in targeted brain therapies. By using [^89^Zr], [^68^Ga] and [^64^Cu], researchers tap into the unique properties of these isotopes for effective, long-term tracking of cells *in vivo*. Despite these advancements, challenges such as the potential toxicity of long-lived radioisotopes and the spatial resolution limitations associated with certain radioisotopes remain. These factors necessitate careful consideration in the design and interpretation of PET imaging studies. Furthermore, the field is evolving, and more research is needed to optimise tracer development, refine imaging methodologies such as advanced PET imaging methodologies, including the development of specific and selective radiotracers, hybrid imaging techniques like PET/CT and PET/MRI for combined functional and anatomical insights, and dynamic imaging with quantitative analysis tools. Furthermore, to fully understand the long-term effects and safety of IN administration in clinical settings, including exploiting more sophisticated nasal dosage forms. The insights gained from these PET studies are invaluable in paving the way for more effective treatments for CNS disorders, where traditional systemic administration is often hindered by the BBB.

### 2.3. Optical imaging

OI continues to be evolving in the field of neuroscience due to its unique ability to view the intricate workings of the brain. The technology holds immense potential for advancing our understanding of INDD systems. OI techniques, leveraging the emission and absorption of light, enable the visualisation of biological processes and structures in the brain with remarkable clarity and precision 114. OI in nose-to-brain applications delineates the pathways and mechanisms involved in the direct transport of substances from nose-to-brain. This non-invasive approach can particularly be useful for studying the dynamics of drug delivery and the distribution of therapeutic agents, in turn leading to valuable insights into the efficiency and effectiveness of IN administration treatments.

At the heart of nose-to-brain studies are optical imaging techniques. Bioluminescence (BLI) is a technique where light is produced as a consequence of a chemical reaction within an organism. Here, the energy needed to produce the light is drawn from the environment (i.e., such as oxygenated blood re-energising cells). This method is highly sensitive and is used for tracking the distribution and persistence of cells or specific genes 115. BLI can be instrumental in monitoring the movement and localisation of IN administration therapeutic agents, enabling researchers to track the progress and effectiveness of treatments in real-time. Fluorescence (FLI), on the other hand, involves the use of fluorescent probes or markers that emit light when excited by a specific wavelength (i.e., externally supplied sources of energy, such as lasers, to produce light emission). This technique provides a method for detailed visualisation of drug movement and interactions within brain tissues. FLI is particularly useful in nose-to-brain studies for mapping the pathways of drug delivery from nose-to-brain and assessing the distribution of therapeutic compounds.

#### 2.3.1. Fluorescence imaging (FLI)

Garzotto and Marchis explored the use of fluorescent quantum dots (QDs), specifically carboxylated CdSe-ZnS (cadmium selenide-zinc sulphide) QDs, as IN administration imaging agents in mice using a pipette, to understand their translocation from nose-to-brain 116. QDs a design of advanced fluorescent semiconductor nanocrystals frequently utilised in cellular and animal biology. The QDs used were Carboxyl ITK QDs 585 and 525, which are fluorescent semiconductor nanocrystals. The delivery pathway targeted the olfactory epithelium, exploring both neuronal and extracellular transport mechanisms. The study focused on the potential routes QDs might take from nose-to-brain, hypothesising the involvement of extracellular pathways such as perineuronal and perivascular spaces and CSF routes. To track the movement and distribution of QDs, confocal microscopy was used to analyse the olfactory epithelium at different time points post-administration. After administering acute IN administration treatments and employing confocal microscopy to observe the QD's journey within the olfactory epithelium. Initially, to determine whether QDs can penetrate and traverse the olfactory epithelium, they conducted a single IN rinse on adult mice and examined the distribution of QDs within the olfactory epithelium over varying durations from 1 to 24 h. One-hour post-rinse, a pronounced fluorescence was detected on the luminal surface of the olfactory epithelium, particularly around the cilia of the olfactory receptor neurons and the microvilli extending from the apical surface of the sustentacular cells. By 4 h, they noted an increased spread of fluorescent spots throughout the thickness of the olfactory epithelium. By 24 h, QDs had reached and accumulated in the olfactory epithelium's basal area. Both 4 and 24 h post-rinse, QDs were also seen in the adjacent lamina propria, with a gradual reduction in QD density over time. Fluorescence was frequently observed in polygonal cells surrounding blood vessels and occasionally in nerve bundles. The study suggested that QDs can traverse the olfactory epithelium and enter the underlying lamina propria. The movement of QDs through the olfactory epithelium might occur via a transcellular route involving neuronal and sustentacular cell bodies, or a pericellular route through extracellular spaces. To further explore the interaction and intracellular positioning of QDs across different environments and neuronal types, specific olfactory receptor neuron labelling was conducted *in vivo*, and neuronal enriched primary cultures from the olfactory bulb were used *in vitro*. *In vivo* experiments co-administered QDs and fluorescein dextran (Dex), a molecule known for its uptake by olfactory receptor neurons and transport to the olfactory bulb via the olfactory nerve through IN rinse. After 4 h post-treatment, fluorescein Dex was found extensively within olfactory receptor neurons, encompassing the entire soma, although confocal multi-stack analysis revealed no colocalisation with QDs. This finding not only demonstrates the potential of QDs in brain imaging applications, but also underscores the importance of further research into their long-term effects and clearance mechanisms, whilst addressing growing concerns about the safety of NPs in biomedical applications.

**Figure 7:**
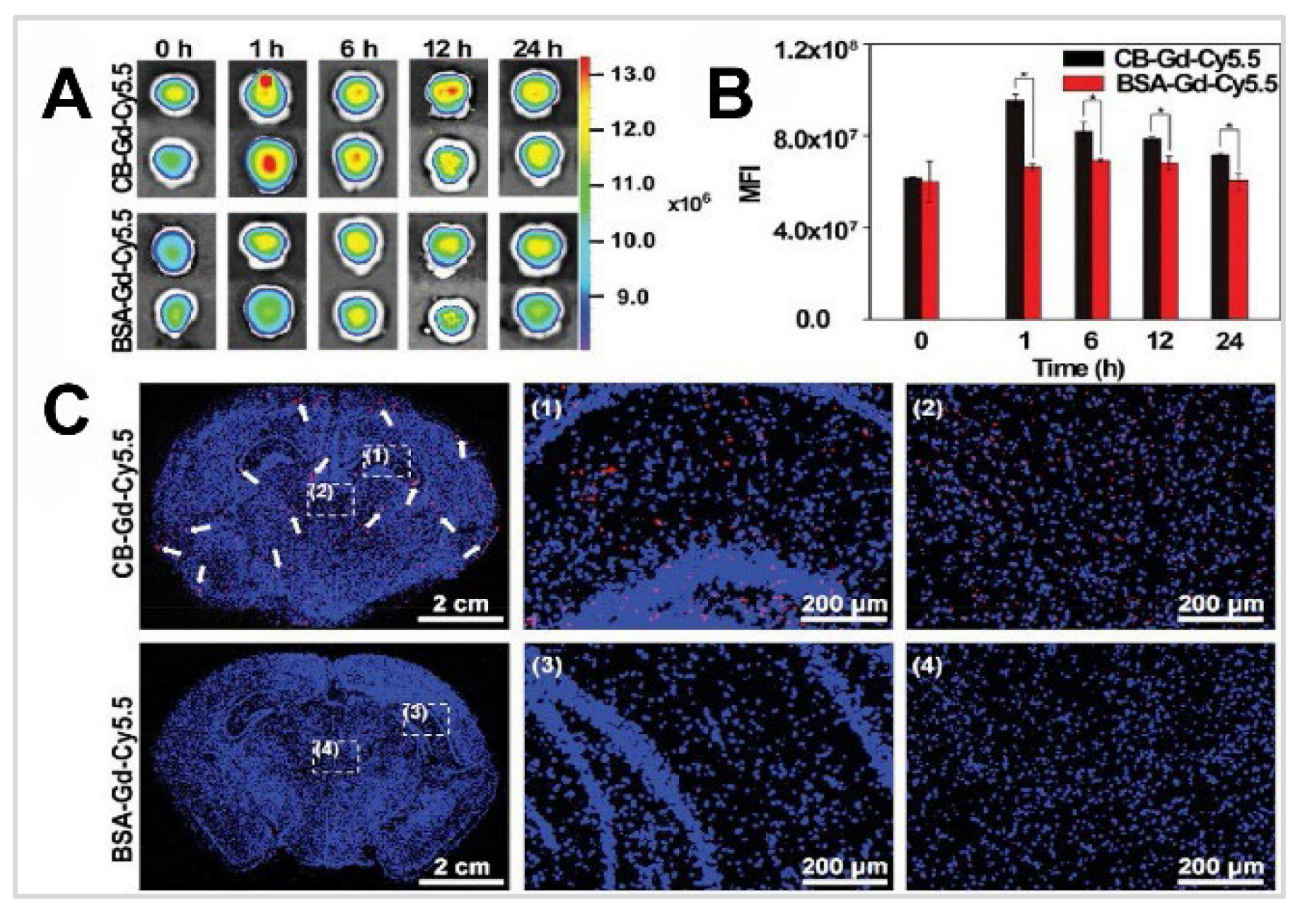
A) *Ex vivo* fluorescence images and B) Analysis of fluorescence intensity in mouse brains prior to and at 1, 6, 12, and 24 h following IN administration of CB-Gd-Cy5.5 or BSA-Gd-Cy5.5. C) Fluorescence microscopy of brain slices from mice 1 h post-IN administration of CB-Gd-Cy5.5 or BSA-Gd-Cy5.5, with white arrows highlighting areas of CB-Gd-Cy5.5 presence. 1-4 representative fluorescence images of brain sections from mice at 1 h post-IN administration CB-Gd-Cy5.5 or BSA-Gd-Cy5.5. Adapted with permission from 118, copyright 2019 Wiley.

A study investigated the distribution of Cyanine 5.5 (Cy5.5) when delivered either IN administration or IV injection 117. Using a small animal fluorescence live imaging system, significant differences were found in the distribution of Cy5.5 based on the delivery method. IN administration primarily resulted in fluorescent signals in the stomach but not in the liver. Conversely, IV injection led to strong signals in the liver but not in the stomach, with fluorescent signals detected in multiple brain regions. Furthermore, significant accumulation in the olfactory bulbs within 1 to 2 h after IN administration, indicating high concentrations of Cy5.5, whereas such concentrations were not observed in other brain regions. The findings highlight distinct differences in the pathways and distribution patterns of Cy5.5 between IN administration and IV injection, particularly emphasising the olfactory bulbs as a primary site for drug accumulation following IN administration. In a different study, a stable protein-based NPs system, CB-Gd-Cy5.5, using Cy5.5 as a model drug, was developed 118. The focus was on tracking the distribution and metabolism of CB-Gd-Cy5.5 in the brain following IN administration. Using a filled pipettor, each mouse receives a 10 µL volume of the solution. Findings showed widespread fluorescence signals throughout the brain within 1 h of administration, persisting up to 24 h (see Figure 7). This highlighted the system's efficiency in delivering drugs to the brain via the nose-to-brain pathway, outperforming the bovine serum albumin (BSA) BSA-Gd-Cy5.5 system. A key observation was the significant signal enhancement in the hippocampus shortly after administration, suggesting effective delivery of the CB-Gd-Cy5.5 NPs to this region. This was likely facilitated by the CB to the GM1 ligand on nerve cell membranes. Quantification of the tracer revealed that the fluorescence signal in the brain for the CB-Gd-Cy5.5 group was 22 times greater than in the liver, underscoring the efficiency of the delivery system. Over time, the fluorescence signal declined, suggesting gradual clearance of the NPs from the brain. Histochemical analysis, including Hematoxylin and eosin and Nissl staining, indicated that the CB-Gd-Cy5.5 NPs did not cause noticeable histopathological abnormalities or tissue damage in the brain or other major organs. The Nissl staining showed no significant neuronal loss or disappearance of nuclei, confirming the safe use of the NPs with brain tissue. Overall, the CB-Gd-Cy5.5 NPs demonstrated promising potential for brain targeting via IN administration, with effective delivery to the hippocampus, prolonged retention, and minimal off-target effects, as confirmed by advanced imaging techniques and histological analysis. This study provides a robust framework for the design of protein-based multifunctional drug delivery systems aimed at treating neurodegenerative diseases like Alzheimer's (MRI data will be explained in section 2.4.1).

A study employed a multimodal approach to analyse the spatiotemporal distribution of gold nanorods (AuNRs) following IN administration, utilising OI, gamma counting, and autoradiography as complementary techniques 119. The delivery device used for administration was a micropipette, and a total volume of 20 μL was administered intranasally. OI, based on the fluorescent properties of Cy5.5-labeled AuNRs, provided qualitative insights into the *ex vivo* organ distribution profiles, enabling the semi-quantitative tracking of AuNR particles in specific organs, especially the brain, as a rapid screening method. Gamma counting facilitated data on organ biodistribution profiles of ^111^In[DTPA (diethylenetriamine pentaacetate)-AuNR_PEG-NH2_] in mice and led to information on AuNR uptake over time. Autoradiography was applied to investigate the regional distribution of ^111^In[DTPA-AuNR_PEG-NH2_] in the brains, revealing a rapid uptake within 10 min post-administration, indicative of an efficient delivery mechanism. Initial uptake was predominantly in the olfactory bulbs, then over time, the AuNRs diffused into adjacent regions such as the olfactory tract, anterior olfactory nucleus, and piriform cortex, with evidence of deeper brain region involvement, including the brainstem, within an hour. OI confirmed successful translocation to the brain, with higher fluorescence signals in the frontal brain region. Gamma counting revealed that about 30% ID/g remained in the nasal passage, and radioactivity in the brain peaked at 10 min, measuring 0.036% ID/g, post-administration, gradually decreasing over time. Autoradiography supported these findings, showing region-specific brain accumulation, notably in the olfactory bulbs, elucidating the involvement of the olfactory and trigeminal pathways in the intranasal delivery of AuNRs. This comprehensive approach revealed the spatiotemporal dynamics of AuNRs in the brain, providing crucial insights for the development of IN AuNRs as potential INDD carriers for CNS applications. The retention of AuNRs in the brain showed a marked decrease over time. High levels were detected at 10 min, but by 3- and 7-days post-administration, the Au content had significantly diminished, interpreted as limited long-term retention. This temporal reduction was confirmed through various imaging modalities, including OI, inductively coupled plasma mass spectrometry, and nuclear imaging techniques. The study employed multi-modal imaging agents to characterise the distribution and clearance of AuNRs. The Cy5-functionalised AuNRs were tracked using OI, while the DTPA-functionalized AuNRs complexed with [^111^In] allowed for nuclear imaging. This approach highlighted the initial high uptake in the olfactory regions and subsequent broader distribution to other brain areas over time. Quantitative measurements via inductively coupled plasma mass spectrometry found the highest brain uptake at 10 min post-administration, with 39.71 μg Au/g of brain, decreasing to 0.59 μg Au/g by day 7. Gamma counting provided further quantitative biodistribution data, revealing an initial brain uptake of 0.04% ID/g of tissue. The AuNR's elimination from the brain predominantly occurred through mucociliary clearance, transitioning the particles to the gastrointestinal tract for excretion via faeces. This research emphasises the importance of using multi-modal imaging approaches combined with high-resolution histochemical analysis to validate the biodistribution and therapeutic potential of intranasally administered agents. The integration of such techniques offers a comprehensive understanding of the localisation and kinetics of drug delivery systems, enhancing their application in treating CNS diseases.

In Scranton *et al.*'s study, fluorescent tag CellTracker Green (CTG) BODIPY and radiolabelled tracers, [^125^I]EPO (erythropoietin) and [^125^I]calcitonin, were used to investigate the transport mechanisms within the rostral migratory stream for INDD to the brain 120. The researchers used a micro syringe to administer 6 µL of the tracer solution to mice's nasal cavities. The study aimed to understand how these substances are transported to various brain areas via the rostral migratory stream. This nerve, which connects the olfactory bulb to the periventricular regions, facilitates the passage of substances through a paracellular mechanism, allowing them to traverse the nasal mucosa and bypass the BBB. The uptake of the tracers was assessed at multiple time points, specifically 20-, 60-, and 120-min post-administration. The results showed significant amounts of tracers in the brain as early as 20 min after administration. Notably, regions such as the olfactory bulb, hippocampus, cortex, cerebellum, and choroid plexus exhibited high levels of tracer presence. The retention indicated that these levels remained significant within the 120 min observation window. Their observations suggested a paracellular transport mechanism, where substances move between cells, rather than within them, indicating that the physical structure of the rostral migratory stream is crucial for this process. When the rostral migratory stream was intact, efficient delivery to the brain was observed, with ~41% ID of [^125^I]-EPO and ~19% ID of [^125^I]calcitonin reaching the brain within 20 min after IN administration. Whereas, when the rostral migratory stream was surgically transacted, the normal path of these tracers to the brain was significantly hindered, with only about 3% ID of [^125^I]EPO and 4% ID of [^125^I]calcitonin, leading to their accumulation in peripheral organs. This finding not only highlights the essential role of the structure of the rostral migratory stream in facilitating direct CNS delivery, but also suggests longer-term changes, such as reactive astrogliosis from surgical disruption, could affect transport. CTG fluorescence was observed extensively in brain regions without a detectable presence in peripheral tissues. Liquid scintillation analysis of [^125^I]EPO and [^125^I]calcitonin confirmed high CNS concentrations in intact rostral migratory stream mice, supporting the efficiency of this delivery route. The study highlights the rostral migratory stream in INDD as a pathway to the brain. However, it does require further research, especially regarding its application in human medicine. Adding to the context of these findings, published results from autoradiographs have indicated that IN administration radioligands distribute throughout the brain without being confined to or concentrated in the olfactory areas innervated by the olfactory nerve 121. Moreover, the tracers used in Scranton *et al.*'s study did not show a preference for the projection areas of the olfactory nerve. These results propose that the rostral migratory stream, rather than the surrounding olfactory nerve, may serve as the main transport route, or alternatively, that substances transported by the olfactory nerve might bypass the olfactory region of the brain. This suggests the involvement of additional pathways, with the intact trigeminal nerve offering a potential uptake path into the CNS.

Subsequently in 2011, the researchers investigated the potential of MSCs, labelled with enhanced green fluorescent protein (EGFP) and made available via IN administration using a pipette, to migrate to and survive in the mouse brain having striatal lesions 122. The MSCs, harvested from genetically modified mice expressing EGFP, served as a fluorescent imaging agent to track their transport. Despite employing sophisticated imaging methods like fluorescence microscopy, the MSC-EGFPs were not detectable in the brain from 3 h to 2 months post-administration. This observation raised questions about the viability and migration capability of MSCs. Surprisingly, a significant autofluorescent signal, mimicking the GFP emission, was observed in both the olfactory bulb and striatum of mice, regardless of whether they received MSC-EGFP treatment or were part of the control group. This autofluorescence complicated the interpretation of results, emphasising the need for meticulous controls in experiments using GFP as a tracking method. In contrast, a control experiment where MSC-EGFPs were directly implanted into the striatum of mice showed robust GFP expression at 1- and 7-days post-implantation, confirming the cell's survival and GFP expression in a different delivery context. Under the conditions of this study, the findings suggest that MSC-EGFPs failed to survive or migrate within the brain, highlighting the complexities of cell-based therapies and the critical role of appropriate control experiments. Furthermore, high-resolution histochemical analysis did not confirm the presence of MSC-EGFPs in the brain. Electron microscopy depicted cell-like structures filled with inclusion bodies and lipofuscin, indicative of tissue degradation, rather than GFP-specific signals. In summary, the IN delivery of MSC-EGFPs did not result in the migration or survival of these cells in the brain. The autofluorescence observed in the olfactory bulb and striatum, which could be mistaken for a GFP signal, underscores the necessity of including rigorous controls when using GFP as a marker. This study highlights the challenges of confirming the localisation of intranasally delivered cells or drugs using high-resolution imaging and histochemical techniques. The findings emphasise the need for multiple validation methods to accurately differentiate true signals from artifacts, ensuring the reliability of the results.

In 2012, two studies explored the IN administration of insulin as a novel approach to treat CNS disorders. The first study focused on the use of an ME method, where fluorescein isothiocyanate-labelled insulin (FITC-insulin) was administered to rats in a water-in-oil formulation 123. The insulin was administered intranasally with a volume of 5 µl per nostril using a pipette tip positioned at the nostril opening. The study found significant fluorescence levels in the brain as early as 24 h after a single IN administration of the ME formulation. This rapid uptake indicates the effectiveness of the olfactory route in delivering insulin directly to the brain. When comparing different delivery methods, the ME formulation resulted in a 2-fold higher fluorescence signal in brain sections, particularly in the olfactory bulb and frontal lobes, compared to the aqueous solution. This suggests a higher brain uptake with the ME system. Retention times varied depending on the frequency of administration. For a single dose, significant brain uptake was observed 24 h post-administration. For twice-daily doses, the ME formulation continued to show enhanced brain uptake. However, when administered thrice daily, there was no significant increase in brain uptake, possibly due to enzymatic degradation within the olfactory bulb, which limited further uptake. Quantification of the insulin reaching the brain showed that a single IN dose of the ME resulted in an average fluorescence level of ~86 pdu, while the aqueous solution achieved ~51 pdu. With 2 doses administered 12 h apart, the ME showed ~139 pdu compared to 49 pdu for the aqueous solution. After 3 doses, the ME resulted in ~66 pdu, whereas the aqueous solution had ~36 pdu. This quantification highlights the superior brain uptake efficiency of the ME formulation. The elimination of the tracer involved enzymatic degradation within the olfactory bulb, particularly with repeated doses. This degradation may limit further brain uptake due to increased protein concentration. Fluorescence microscopy provided detailed imaging of the fluorescently labelled insulin distribution in brain sections. This method allowed for precise quantification and localisation of insulin within brain tissues, supporting the study's findings. Overall, the study demonstrates that IN delivery of insulin via an ME system is significantly more effective in targeting the brain than aqueous solutions or IV injection. This method has potential therapeutic applications for neurological disorders, such as Alzheimer's disease, by effectively bypassing the BBB and directly targeting the brain. The second study focused on specific pathways utilised in IN administration insulin delivery to the CNS 124. The study utilised a pipette to intranasally administer insulin, delivering a total volume of 24 µL of tracer solution. This mouse study used both fluorescent and electron microscopy 30 min post-administration to track the insulin's journey. The primary delivery pathway identified was the olfactory nerve pathway. Insulin administered intranasally traversed this pathway, which involves extracellular delivery in perineuronal spaces surrounding olfactory nerve axons extending from the olfactory mucosa to the anterior regions of the olfactory bulbs. Confocal microscopy confirmed a continuous stream of fluorescently tagged insulin moving from the neuroepithelium of the olfactory mucosa, through the cribriform plate of the ethmoid bone, and into the olfactory bulbs. This rapid delivery route aligns with previous findings suggesting that extracellular transport provides quicker access to the olfactory bulbs compared to intracellular transport within the axons, which takes several hours. The study demonstrated significant uptake of insulin within 30 min of administration. Fluorescent and electron microscopy showed that insulin reached the anterior regions of the olfactory bulbs. Higher concentrations were observed in the olfactory nerve layer compared to the glomerular layer, with the fluorescent label preferentially localising within the periglomerular cells of the glomerular layer. This distinct distribution pattern highlights the effectiveness of the olfactory nerve pathway in targeting the olfactory bulbs quickly after IN administration. Retention of insulin within the olfactory nerve bundles and layers was evident shortly after administration, suggesting active uptake and possible endocytosis by cells in these regions. Electron microscopy revealed intracellular nanogold-labelled insulin within the olfactory nerve layer and glomerular layer. Insulin was localised in the cytoplasm, along the nuclear membrane, and associated with the heterochromatin of the nucleus within neurons. This suggests that the insulin was not only delivered but also actively transported and potentially functional within these cells. The study utilised two types of imaging agents: Alexa Fluor 647 labelled insulin for macroscopic and confocal imaging and nanogold labelled insulin for electron microscopy. Fluorescent imaging showed a continuous extracellular distribution along the olfactory nerve bundles, while nanogold imaging revealed intracellular uptake within olfactory bulb neurons, indicating active transport and potential functional activity of the delivered insulin. This dual approach provided a comprehensive view of insulin distribution and uptake, confirming the olfactory nerve pathway as an effective route for CNS targeting via intranasal administration. Together, these studies present a viable and effective method for delivering therapeutic agents like insulin directly from nose-to-brain.

The comparative analysis of the studies on insulin labelled with different imaging agents reveals crucial insights into their pharmacokinetics, regional brain uptake, and therapeutic implications. The [^18^F]-labelled insulin, studied by Smith *et al.*, demonstrated rapid and efficient brain delivery via a catheter-based liquid method, making it highly effective for targeting limbic and frontotemporal regions critical for Alzheimer's disease treatment 2. The [^68^Ga]-NOTA-labelled insulin, as presented by Gollapelli *et al.*, exhibited high stability and provided valuable diagnostic insights into Alzheimer's disease, particularly regarding insulin resistance mechanisms 109. The FITC-labelled insulin, administered through a water-in-oil ME formulation, showed superior brain uptake efficiency, though enzymatic degradation with repeated doses posed a limitation 123. Lastly, the Alexa Fluor 647 and nanogold-labelled insulin demonstrated effective CNS targeting via the olfactory nerve pathway, with significant intracellular uptake and potential functional activity within neurons 124. Collectively, these studies emphasise the importance of selecting appropriate tracers and delivery methods tailored to specific therapeutic or diagnostic goals, ultimately advancing the potential of nasal delivery systems for treating and diagnosing Alzheimer's disease.

In a study for fast transport within the cerebral perivascular conducted by Lochhead *et al.*, they used Texas Red-labelled Dex's (TR-Dex) of 3 and 10 kDa as fluorescent imaging agents to investigate the mechanisms and distribution of tracer transport in the brain following IN administration in rats 125. With delivery carried out using a micropipette, upon IN administration, the tracers utilised the olfactory and trigeminal nerve pathways, rapidly reaching the brain and distributing via the perivascular spaces of cerebral blood vessels. The study used *ex vivo* fluorescence imaging and confocal microscopy to demonstrate that within 20 min, TR-Dex3 was prominently distributed within the perivascular spaces of surface arteries and internal cerebral vessels. For TR-Dex10, a similar distribution was observed within 30 min, but only when an absorption enhancer, matrix metalloproteinase-9, was used, indicating a size-dependent barrier at the nasal epithelium. The uptake regions included the olfactory bulbs, trigeminal nerves, and various brain regions such as the cortex and hippocampus. The study highlighted that significant levels of tracers were observed in the perivascular spaces surrounding leptomeningeal vessels on the brain's ventral and lateral surfaces. The retention time was primarily noted within the first 20-30 min post-administration, with fluorescence signals indicating widespread brain distribution. Quantification of the tracers revealed that IN administration resulted in significantly higher brain levels compared to intraarterial administration. TR-Dex3 showed a 24.2-fold increase in perivascular fluorescence intensity after IN delivery compared to intraarterial administration. While the study did not delve into the detailed mechanisms of tracer elimination from the brain, it suggested that substances might drain via the CSF and lymphatic pathways. Confocal microscopy confirmed the imaging results, showing detailed localisation of TR-Dex3 and TR-Dex10 in brain sections. The tracers were predominantly associated with the perivascular spaces of blood vessels and surrounding brain parenchyma, supporting the observed rapid and widespread distribution post-IN administration. The study systematically compared the two types of imaging agents, TR-Dex3 and TR-Dex10, while highlighting their distinctive distributions within the brain. TR-Dex3 showed efficient brain uptake and distribution without the need for absorption enhancers, whereas TR-Dex10 required matrix metalloproteinase-9 pretreatment to achieve similar results. This comparison emphasizes the importance of molecular size in determining the permeability and distribution efficiency of intranasally administered agents. This research revealed the potential of INDD as a promising approach for treating diseases with cerebrovascular pathology.

In a study by Stanojević-Vitaliano, the potential of clathrin triskelia, a natural coat protein, NPs as a nanoplatform for delivering antibodies and imaging agents to the CNS was explored 126. The study involved the use of a 10 μL Hamilton syringe for IN administration. FITC has tagged clathrin triskelia and tested its ability to noninvasively penetrate the rat brain. Fluorescently labelled NPs were observed in the brain as early as 30 min after IN administration, indicating rapid uptake. The dopamine 3 receptor antibodies-triskelia successfully targeted dopamine 3 receptor brain regions such as the olfactory tubercle, islands of Calleja, ventral pallidum, nucleus accumbens, striatum, frontal/parietal cortex, hippocampus, medial mammillary bodies, anteroventral thalamic nucleus, substantia nigra/ventral tegmental area, and cerebellum. The retention time varied, but nanoprobe distribution in brain regions was evident 3 h post-administration, suggesting sustained presence in the target regions. Immunohistochemistry confirmed the localisation of dopamine 3 receptor antibodies in targeted brain regions. Positive signals from anti-dopamine 3 receptor antibodies were detected in regions such as the nucleus accumbens, islands of Calleja, hippocampus, and substantia nigra. The study also used fluorescent dopamine 3 receptor antibody-Triskelia, where fluorescent signals detected in dopamine 3 receptor brain regions confirmed specific targeting, showing punctate deposits within discrete cellular structures. Rhodamine-triskelia without targeting antibodies was found in all brain regions, including the prefrontal cortex, indicating nonspecific distribution. These findings highlight the potential of clathrin-based NPs in transporting large molecules, like antibodies, into the CNS, offering new avenues for diagnosing and treating CNS disorders.

In a cargoes but not vehicles study, Ahmad *et al.* tracked the NEs and their cargoes for nose-to-brain delivery using a micropipette 127. The mechanisms facilitating this delivery included the use of mucoadhesive polymers and the careful selection of particle size. Due to their mucoadhesive properties, chitosan-coated NEs were used to increase retention time in the nasal cavity. Additionally, thermosensitive *in situ* gels made from Poloxamer 407 and Poloxamer 188 were employed to further prolong the residence time of NEs in the nasal mucosa. NEs with particle sizes around 100 nm demonstrated superior retention and penetration through the mucosal layer compared to larger particles, as *in vivo* imaging revealed their presence in the mucosa and along the trigeminal nerves within 1 h post-administration. The mechanism involved passage through the olfactory and trigeminal nerves, smaller NEs (80-200 nm) were retained in the nasal mucosa for up to 16 h, whereas larger particles (500-900 nm) were cleared more quickly. The chitosan-coated NEs had enhanced retention compared to their non-coated counterparts. In terms of brain uptake, only weak signals from the environment-responsive probes (P2 and P4) were detected in the olfactory bulb and brain, indicating that only minute quantities of integral NEs reached these areas. Fluorescent signals captured at various time points (up to 16 h post-administration) indicated a gradual, size-dependent translocation. The cargoes within the NEs, represented by DiR and Cou-6 signals, were found in significant amounts along the nose-to-brain pathway and in the brain, suggesting that the cargoes were effectively released and transported. Very minimal amounts of NEs were detected in the blood or vital organs, suggestive of negligible systemic absorption. High-resolution histochemical findings from confocal laser scanning microscopy provided detailed images showing intense signals of the cargoes (Cou-6) in various brain regions. This supported the conclusion that the cargoes, rather than the intact NEs, were the primary agents reaching the brain. *Ex vivo* imaging further validated these findings, showing weak P2 signals (representing NEs) in the olfactory bulb and brain, but strong signals for the cargoes. In summary, the study demonstrates that while integral NEs have limited direct transport to the brain, the cargoes they carry can effectively permeate brain tissues via the nose-to-brain pathways. The histochemical analysis confirmed the imaging data, showing the localisation and distribution of the drugs within the brain. These findings suggest that the primary role of NEs in nose-to-brain delivery is to facilitate the transport of their cargoes, rather than the carriers themselves. This provides important insights into the mechanisms of INDD and highlights the potential of NEs for enhancing brain drug delivery. In another study, DiR-labelled PLGA NPs were employed to investigate the distribution of edaravone, a drug for amyotrophic lateral sclerosis, following IN administration and IV injection in mice 128. The delivery device used for IN administration was a micropipette, with 10 µL of the NP suspension instilled into each nostril. The uptake of edaravone was rapid, with comparable amounts (~353 ng/g) detected in the brain at 0.5 h post-administration for both IN and IV routes. The NP formulation provided prolonged retention, with significant amounts still present at 12 h (~213 ng/g) and 24 h (~60 ng/g) post-IN administration. This sustained presence indicates the effectiveness of the IN route in maintaining therapeutic levels of the drug in the brain over time. The study identified specific brain regions with high uptake, including the olfactory bulbs, hippocampus, and cerebral cortex, which are crucial areas affected by neurodegenerative diseases like amyotrophic lateral sclerosis. In addition to the brain, IN administration resulted in uptake in other organs such as the lungs, spleen, liver, kidneys, stomach, and intestines, although to a lesser extent compared to IV injection, indicating a more localised and targeted delivery to the brain. Quantitatively, the IN route achieved ~0.8% ID/g, significantly higher than the IV route ~0.26% ID/g at 0.5 h. This higher efficiency underscores the potential of the IN pathway in delivering therapeutic doses directly to the brain with minimal peripheral distribution. Elimination and clearance of the drug varied between the two routes. IN administration showed limited systemic exposure and negligible liver uptake, suggesting efficient targeting and reduced risk of systemic side effects. In contrast, the IV route exhibited higher systemic distribution with significant initial liver uptake, which decreased over time. The study primarily relied on FLI and ultra-performance liquid chromatography-tandem mass spectrometry quantification to track the biodistribution and retention of edaravone. The imaging and quantification methods used provided robust evidence of the drug's presence and distribution in the brain.

A study by Xu *et al.* on the IN administration of icariin for treating depression developed an icariin nanogel loaded with a self-assembled thermosensitive hydrogel system, using rhodamine B-labelled nanogels as the imaging agent for tracking 129. This formulation targeted the brain with rapid uptake observed within 30 min post-administration, leveraging the IN route for direct and efficient drug delivery to the brain. Retention of icariin in the brain was notably prolonged. The fluorescence signals from the rhodamine B-labelled nanogels were sustained in the brain for up to 24 h, demonstrating the sustained release properties of the icariin-nanogel system. This prolonged retention is indicative of the drug's potential for long-lasting therapeutic effects following a single administration. The elimination of the tracer involved systemic distribution to other organs, primarily the liver and kidneys, which were identified through subsequent fluorescence signals. That is, after initial uptake in the brain, the drug was gradually distributed systemically and eliminated through metabolic and excretory pathways. Hematoxylin and eosin staining of the hippocampal tissues in the rat models showed significant morphological improvements in neuronal cells within the CA1 and CA3 subregions of the hippocampus in the icariin nanogel treated group compared to the chronic unpredictable mild stress group. These histopathological results highlighted the potential neuroprotective effects of icariin-nanogel, suggesting its efficacy in reversing stress-induced neuronal damage. The use of rhodamine B-icariin nanogels as imaging agents facilitated a clear understanding of the drug's distribution, showcasing its quick localisation to the brain, sustained retention, and subsequent systemic distribution and elimination. The comparative analysis of imaging agents demonstrates the efficiency of the icariin-NGSTH system in delivering and retaining therapeutic agents in the brain.

A later study focused on glioblastoma treatment using a disulfiram-loaded ion-sensitive NE *in situ* gel (DSF-INEG) delivered IN with copper ions 130. The study utilised a microlitre syringe to intranasally administer the DSF-INEG into one nostril (left or right) of the rats. The administered volume was approximately 100 µL. The study's imaging findings confirmed this efficient transport, with fluorescence imaging revealing significantly higher fluorescent intensity in the brain compared to other organs at various time points (1, 3, and 6 h post-administration). The primary regions showing uptake included the olfactory bulb and broader brain tissue, indicating effective transport through the olfactory and trigeminal nerve pathways. Cy5.5, a fluorescent dye, was used as a surrogate marker to assess the brain targeting capability. Cy5.5 is a lipophilic dye that matches the poorly soluble nature of disulfiram, making it a suitable choice for tracking the NE's distribution. The strong brain-targeting capability of Cy5.5-INEG via nose-to-brain transport was evidenced by significantly higher fluorescent intensity in the brain than in other organs. This finding indicated the formulation's effectiveness in delivering the drug to the brain. The retention time of the DSF-INEG formulation in the brain was notable for its sustained release properties. *In vitro* release profiles indicated that the formulation could maintain therapeutic levels in the brain over an extended period, with 50% of the drug released at 4 h and approximately 75% at 12 h. This sustained release is critical for prolonged therapeutic effects against glioblastoma. Quantification of the drug reaching the brain was inferred through comparative fluorescence imaging intensities. The fluorescence intensity of brain tissues in rats treated with Cy5.5-INEG was significantly higher than in control groups, resulting in effective brain targeting and quantifiable delivery. GFP imaging and Hematoxylin and eosin staining were used to assess tumour growth and tissue health. Enhanced tumour growth inhibition was observed in the DSF-INEG/Cu treated rats, evidenced by weaker fluorescence signals in brain tumours and less tissue deterioration compared to control groups. Histopathological analysis revealed no significant differences or pathological changes in the major organs of treated rats, indicating high safety and effective targeting of the brain tumour with minimal off-target effects.

**Figure 8:**
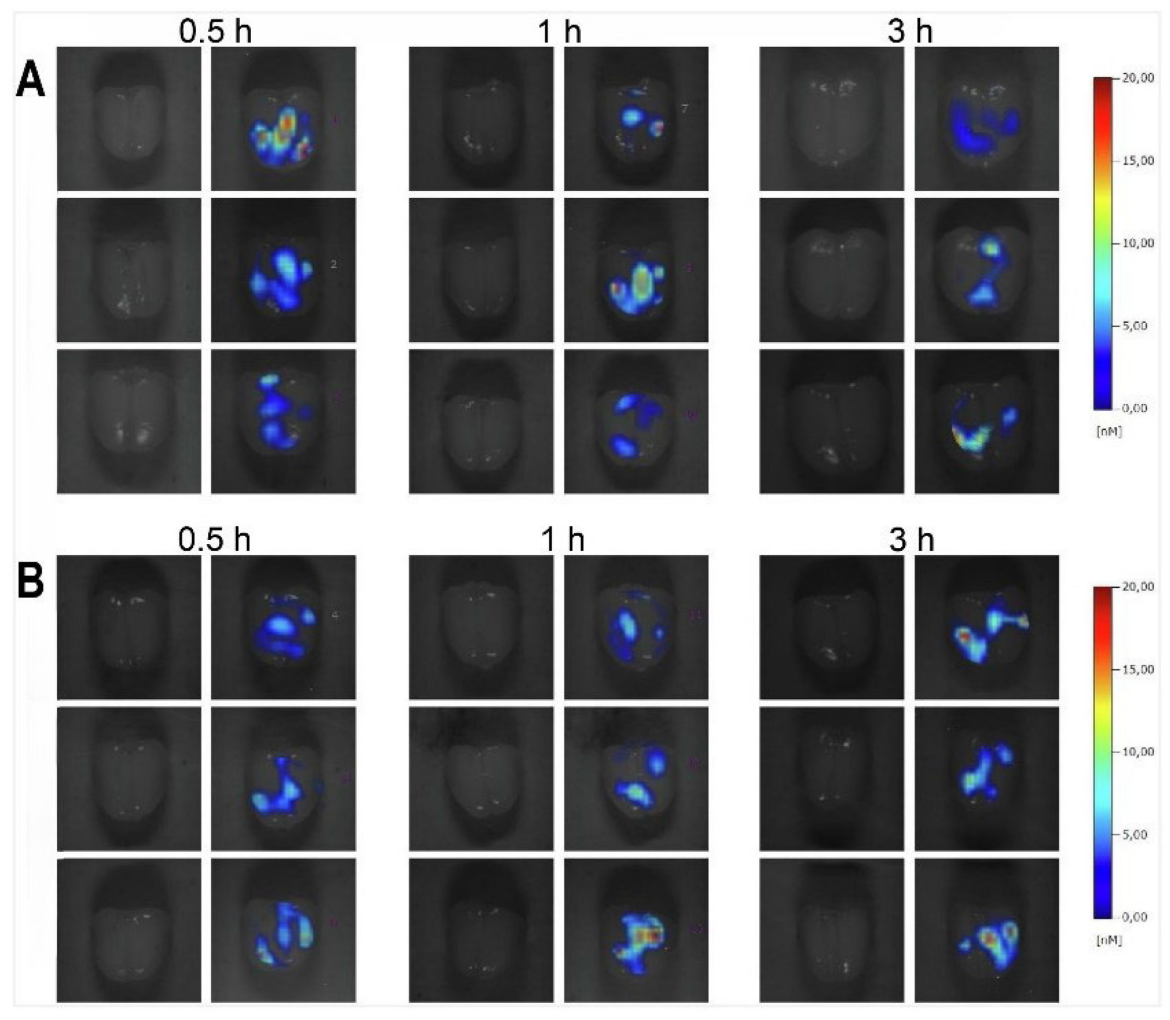
Illustrating the *ex vivo* brain fluorescence tomography. It depicts the brain images at different time intervals (0.5 h, 1 h, and 3 h) following the IN administration of IR780-labelled CHC-loaded NPs (panel A) and IR780-loaded conjugated NPs (panel B), along with a separate column representing negative controls. "Adapted with permission from 131 copyright 2021 ELSEVIER".

Ferreira and team developed an nose-to-brain co-delivery system for glioblastoma treatment, employing Poly (D,L-lactic-co-glycolic acid)/chitosan NPs 131. The delivery device used in the study was a micropipette, with 40 μL of NP solution divided equally between both nostrils. These NPs were uniquely loaded with alpha-cyano-4-hydroxycinnamic acid (CHC) and conjugated with the monoclonal antibody cetuximab. The system's effectiveness in targeting the brain was evaluated using IR780, a near-infrared fluorescent dye, as the imaging agent. FLI was used to observe the brain uptake and distribution of these NPs. CHC-NP showed intense brain fluorescence signals as early as 30 min post-administration, indicating rapid uptake. In contrast, conjugated NP maintained stronger fluorescence signals over a 3 h period, suggesting prolonged retention and effectiveness in brain uptake. The strong fluorescence signals suggest effective transport and retention of the drug in the brain. This qualitative evidence was crucial for validating the nose-to-brain delivery method. This uptake difference between the NPs indicates that conjugated NP has a better retention profile compared to CHC-loaded NP, making it more suitable for prolonged therapeutic effects. *Ex vivo* brain FLI was conducted at multiple time points following IN administration, 0.5, 1, and 3 h (see Figure 8). Early fluorescence signals at 0.5 h indicated quick NP accumulation in the brain, with an increased intensity observed at 1 h, reflecting a higher concentration of the IR780-loaded, drug-conjugated NPs. By 3 h, the imaging either demonstrated a sustained NP presence in the brain or the onset of clearance from brain tissue.

In 2023, Ye *et al.* investigated an approach for enhancing drug delivery to the brain using transcranial magnetic stimulation and fluorescent magnetic NPs 132. The study utilised a syringe pump for IN administration. The study utilised a transcranial magnetic stimulation device to generate time-varying magnetic fields, distinct from static magnets, to attract fluorescent magnetic NPs to brain tissues. Since transcranial magnetic stimulation typically generates high-intensity fields for clinical neurostimulation, the study employed a lower intensity, less than 50% of the resting motor threshold, to move fluorescent magnetic NPs without inducing nerve stimulation. This approach was shown to significantly increase the fluorescence intensity of fluorescent magnetic NPs in the brain, indicating successful delivery. The effectiveness of fluorescent magnetic NP delivery was observed to improve with increasing magnetic field strength, with 1.5- to 1.8-fold increases in brain fluorescence at 25% and 50% resting motor threshold, respectively, compared to the control group 132. Further, the study evaluated the impact of infusion rate on delivery efficiency. Slower injection rates resulted in greater fluorescence intensity in the brain, suggesting that rapid flow rates might limit fluorescent magnetic NP exposure to the magnetic field. Additionally, the size of the NPs played a crucial role in their brain uptake. Smaller NPs (less than 100 nm) were more efficiently delivered to the brain, aligning with the diameter of olfactory axons, which facilitate passive transport to the brain. Interestingly, even larger fluorescent magnetic NPs (up to 300 nm) showed increased brain delivery under the influence of the transcranial magnetic stimulation-induced magnetic force. The study also noted that the biodistribution of fluorescent magnetic NPs was predominantly in the liver and kidneys, with no significant differences observed in other major organs between the transcranial magnetic stimulation and control groups. Hematoxylin and eosin staining were performed on the brain and other major organs. The results showed no gross or microscopic anomalies, inflammation, or fibrosis in the tissues examined. Despite high levels of NPs in the liver and kidney, no significant lesions were observed, indicating the safety of the experimental protocol.

A study by Han used the fluorescent polystyrene NPs, specifically PS-YG, which are 100 nm-sized NPs tagged with a fluorescent dye 133. They investigated the IN administration uptake, accumulation, and neurotoxic effects in the mouse brain using a micropipette to precisely deliver 24 µL. Using fluorescence microscopy, they visualised the distribution and presence of these fluorescent polystyrene NPs within brain tissues, notably in and around neurons. The study found that, shortly after administration, PS-YG accumulated in the brain with significant uptake evident within just 3 h, leading to an acute neurotoxic response characterised by increased levels of neurotoxic and inflammatory markers. The uptake regions include the olfactory bulb, cerebral cortex, cerebral nuclei, hippocampus, thalamus, hypothalamus, midbrain, cerebellum, and hindbrain. The retention of PS-NPs within the brain was evident, as fluorescence signals were still present 24 h after administration. Elimination of the PS-NPs from the brain primarily occurred through exocytosis. The study found that inhibiting histone deacetylase 6 significantly enhanced the exocytosis of PS-NPs from neurons, thereby reducing their intracellular accumulation and associated neurotoxicity. This was evident from the decreased fluorescence signals in neurons treated with the histone deacetylase 6 inhibitor, ACY-1215 (ricolinostat). Conversely, blocking exocytosis with an inhibitor like endosidin2 prevented the clearance of PS-NPs, leading to sustained neurotoxic stress. Immunofluorescence showed co-localisation of PS-YG with the neuronal marker β3-tubulin, confirming the accumulation of NPs within neurons. Increased levels of glial fibrillary acidic protein, a marker of reactive astrocytes, were observed in the hypothalamus and olfactory bulb, indicating astrocyte reactivation and neuroinflammation. Additionally, oxidative stress markers such as heme oxygenase 1 and apoptotic markers like cleaved caspase-3 were elevated in PS-NP exposed neurons, highlighting the acute neurotoxic response. This approach provided critical insights into the dynamics of PS-NP penetration, their interaction with neuronal cells, and the brain's response to and clearance of these NPs, highlighting both immediate and longer-term effects.

A study by Hu *et al*. employed near-infrared and MRI to evaluate the efficacy of an IN-delivery system using MIL-100, a metal-organic carbonised framework (CF) combined with domperidone using a nebulizer 134. MRI provided insights into the brain entry and distribution of the frameworks. Post-administration, the frameworks accumulated in the olfactory bulbs and other brain regions, evidenced by extensive hypointense regions in the MRI images. These hypointense regions rendered the olfactory bulbs nearly imperceptible, indicating significant uptake. The use of an external magnetic field further enhanced brain permeability, aiding the targeted delivery to specific brain areas. This MRI data correlated with behavioural experiments showing significant improvements in depression-like, anxiety-like, and cognitive behaviours in mice treated with the frameworks with domperidone. The increased dopamine receptor density in the prefrontal cortex and hippocampus, observed through immunofluorescence staining, supported these behavioural improvements. *In vivo* fluorescence imaging and MRI found frameworks with FITC exhibited superior brain permeability compared to FITC alone, indicating effective delivery via IN administration. Magnetic assistance by the external field significantly enhanced brain permeability, with the frameworks primarily accumulating in the olfactory bulbs shortly after administration. Subsequent distribution to other brain regions, including the prefrontal cortex and hippocampus, occurred within the first 24 h, as evidenced by fluorescence intensity and MRI hypointense patterns. This rapid uptake and targeted distribution highlight the efficacy of the IN administration system combined with magnetic guidance for achieving therapeutic effects in the brain. Elimination of the tracer from the brain involved its transfer to metabolic organs, followed by renal excretion. The study observed increased iron content in the lungs and kidneys after 10 days of treatment, suggesting partial entry into the lungs post-nasal administration and eventual clearance via the kidneys. This elimination pathway ensures that the nanospheres do not remain in the brain indefinitely, reducing the risk of potential side effects. Together, the complementary findings from near-infrared and MRI modalities demonstrate the effectiveness of the frameworks with domperidone in delivering therapeutic agents from nose-to-brain. The controlled release triggered by near-infrared irradiation and the enhanced brain permeability facilitated by magnetic fields were crucial for achieving significant improvements in mental health conditions in mice models. The rapid uptake and targeted distribution of the frameworks in the brain regions, including the olfactory bulbs, prefrontal cortex, and hippocampus, within the first 24 h, stress the potential of this delivery system for treating neurological disorders.

A study on the IN administration of chitosan hydrogels labelled with a fluorescent tag (doxorubicin) aimed to develop and evaluate an IN-chitosan hydrogel for the delivery of morphine to achieve fast and prolonged analgesic effects using a spray device 135. The IN administration of the chitosan hydrogel demonstrated a rapid onset of action, with significant analgesic effects observed as early as 5 min post-administration. The maximum analgesic effect, reaching 93% of the maximum possible effect, was achieved at this time point. This rapid uptake is attributed to the direct nose-to-brain migration pathway facilitated by the chitosan hydrogel, which effectively bypasses the systemic circulation and the first-pass metabolism in the liver. Fluorescent imaging studies further elucidated the regions of the brain involved in the uptake of the chitosan hydrogel. The primary absorption occurred in the olfactory bulbs, as indicated by a significantly higher fluorescent signal intensity of 74% in this region compared to the control groups, which showed only 15% intensity. This suggests that the drug was efficiently transported to the olfactory bulbs. The washout profile of the morphine-loaded chitosan hydrogel showed a prolonged duration of action. Following the initial peak effect at 5 min, the analgesic effect gradually decreased but remained significant for up to 2 h, maintaining 27% of the maximum possible effect. Interestingly, a delayed recovery of the analgesic effect was noted 4 h post-administration, increasing to 70% of the maximum possible effect and further rising to 80% at 6 h post-administration. This extended presence and delayed clearance indicate that the chitosan hydrogel formulation allows for sustained release and retention of the drug in the brain, thereby providing prolonged therapeutic effects.

A study focused on the development and application of multifunctional NPs designed to target mitochondria and respond to reactive oxygen species for treating ischemic stroke in rats 136. Two tracers were used for imaging, Cou-6 and Rhodamine B. Cou-6 was used as a model drug for fluorescent imaging, which enables researchers to track the distribution and behaviour of the NPs *in vivo*. The Rhodamine B labelled NPs serve to trace the distribution and release profiles of the therapeutic agents encapsulated within the NPs. Upon IN administration, the multifunctional NPs utilise the nose-to-brain pathway, effectively bypassing the BBB and facilitating rapid delivery to the brain's ischemic regions. The NPs accumulate in the brain rapidly, with detectable fluorescence signals appearing within 1 h post-administration, peaking at 2 h, and remaining visible for up to 24 h. The mitochondria NPs were specifically targeted to the ischemic penumbra, showing higher fluorescence intensity in the left ischemic hemisphere compared to the non-ischemic hemisphere. This targeted delivery was enhanced by the SS-31 peptide, which aids in directing the NPs to mitochondria within the ischemic brain regions. The study also utilised an *in situ* gel for the IN administration of the mitochondrial NPs. This thermosensitive gel prevented nasal runoffs and prolonged the residence time of the NPs in the nasal cavity, thereby improving bioavailability. The IN administration route capitalised on the olfactory and trigeminal nerves to transport the NPs directly to the brain. Over time, the tracer indicated a gradual decline in fluorescence intensity after 2 h. Yet, the signal remained detectable at 24 h post-administration, suggesting a relatively slow clearance rate and prolonged therapeutic effect. The therapeutic efficacy of multifunctional NPs was demonstrated through their ability to alleviate oxidative stress, reduce inflammation, repair mitochondrial function, and decrease apoptosis in the ischemic penumbra. *Ex vivo* fluorescence imaging confirmed the enhanced targeting and distribution of multifunctional NPs in the brain, particularly in the ischemic regions, compared to control groups. This study highlights the potential of multifunctional NPs to provide targeted, effective treatment for ischemic stroke, leveraging advanced imaging techniques to track their distribution and therapeutic impact.

A study explored innovative techniques and agents to enhance the delivery of *N*-acetyl-L-cysteine (NAC) from nose-to-brain using a micropipette 137. It primarily utilised *in vivo* imaging to track NAC's distribution and delivery in rats. The researchers used hyaluronic acid silk fibroin hydrogels, known for their mechanical strength, as the primary biomaterial. To increase their effectiveness, dopamine polydopamine was incorporated into these hydrogels, creating multifunctional hydrogels with high adhesive forces and photothermal response effects. NAC, the primary drug used in the study, was labelled with FITC for fluorescence tracking. The study found that NAC delivery to the brain was significantly enhanced using multifunctional hydrogels combined with near-infrared irradiation. The study revealed that the uptake of NAC was notable within 2 h post-administration, with significant localisation observed in the olfactory bulb and hippocampus regions of the brain. The use of hyaluronic acid silk fibroin NAC hydrogels ensured sustained delivery and retention of NAC in the brain tissues, maintaining a presence for at least 6 hours. The fluorescence intensity measured in the *in vivo* imaging study found FITC-NAC in brain tissues to be approximately 5.4- and 9.0-fold greater following administration with hyaluronic acid silk fibroin FITC-NAC and hyaluronic acid silk fibroin FITC-labelled NAC-near-infrared hydrogels, respectively, compared to FITC-NAC solutions. This rapid and enhanced delivery demonstrated the effectiveness of multifunctional hydrogels and the photothermal response effect in facilitating efficient drug uptake and transport to the brain. The hyaluronic acid silk fibroin NAC hydrogels allowed for controlled release, achieving 57% cumulative release within 6 h. The release rate was further enhanced to 65% with the incorporation of periodic near-infrared irradiation, confirming the photothermal response effect's efficacy. The primary uptake region for NAC was the nasal cavity, from which it was transported to specific brain regions, including the olfactory bulb and hippocampus. FLI in the nasal cavities for FITC-NAC solutions indicated fast mucociliary clearance. In contrast, multifunctional FITC-NAC hydrogels, with or without near-infrared irradiation, demonstrated increased retention in the nasal cavity, resisting mucociliary clearance. This was attributed to the high adhesive forces of the multifunctional hydrogels, which enhanced the retention time and prevented rapid clearance from the nasal mucosa.

A study by Shen *et al.* explored the efficacy of IN administration of MSC-derived small extracellular vesicles for CNS therapy and delivered 20 µL using a micropipette 138. This approach aimed to bypass the BBB and deliver therapeutic agents directly to the brain. The extracellular vesicles were labelled with Cy3 and Cy7 fluorescent dyes and glucose-coated AuNPs. Cy3 was used for fluorescence microscopy and confocal imaging. Cy7 was used for near-infrared fluorescence imaging. MSC-derived small extracellular vesicles were administered intranasally. The distribution of these vesicles was tracked using near-infrared FLI, which revealed rapid and widespread distribution across various brain regions, with significant fluorescence detected as early as 0.5 h post-administration and persisting for up to 6 h post-administration, although the signal diminished by 24 h. Higher radiant efficiency was noted in the olfactory bulb, cortex, striatum, hippocampus, pons, and cerebellum, indicating effective brain delivery. Cellular uptake studies conducted using fluorescence microscopy and confocal imaging demonstrated that multiple brain cell types, including microglia, astrocytes, and neurons, incorporated MSC-derived small extracellular vesicles. Quantitative analysis revealed significant uptake in subcortical regions, with a higher percentage of neurons and microglia incorporating MSC-derived small extracellular vesicles in the hippocampus, pons, and cerebellum compared to astrocytes. This suggests vesicles are effectively distributed and taken up by various brain cells. The perivascular pathway was identified as a crucial mechanism for the distribution of MSC-derived small extracellular vesicles. Confocal imaging showed the presence of MSC-derived small extracellular vesicles within the perivascular space, surrounded by AQP4-bearing astrocytic end feet and PDGFR-β positive pericytes. Transmission electron microscopy images validated the localisation of AuNP-MSC-derived small extracellular vesicles within the perivascular space, indicating their movement along this pathway. As some MSC-derived small extracellular vesicles entered peripheral circulation, as evidenced by the increased fluorescence signals in the liver and kidney, the majority remained localised within the brain. This localisation reduces peripheral distribution and clearance compared to IV injection, enhancing the central delivery of therapeutic agents. These findings highlight the promise of intranasal MSC-derived small extracellular vesicles for treating neurological disorders by leveraging the perivascular transport mechanism to achieve effective central delivery.

The study on rivastigmine nasal spray for Alzheimer's disease treatment demonstrated important findings using FLI to assess nasal spray formulation, olfactory deposition, and brain delivery via VP7 nasal spray devices 139. Rivastigmine, a classic cholinesterase inhibitor, was used in the formulations. *In vivo* imaging tracking with Rhodamine B incorporated into the nasal spray formulations provided visual confirmation of the drug transport process. Fluorescence signals were observed in both the nasal cavity and brain within 15 min post-administration, peaking around 60 min, indicating rapid uptake. *Ex vivo* fluorescence imaging further detailed the drug distribution in the brain, showing strong fluorescence signals in the olfactory bulbs, trigeminus, and multiple brain regions, including the corpus callosum, interbrain, caudate putamen, hippocampus, thalamus, and cerebellum. Additionally, desorption electrospray ionisation mass spectrometry was employed to semi-quantitatively analyse rivastigmine distribution, confirming high-intensity signals in these regions as early as 5 min and providing precise spatial mapping post administration and gradually decreasing from 15 to 300 min. These findings indicated that the drug was successfully transported via both olfactory and trigeminal neuronal pathways. The washout mechanisms were also examined, with ciliary clearance in the nasal cavity removing deposited drugs within approximately 15 min, and brain clearance occurring through the glymphatic system and CSF circulation within a few hours. These imaging results evidence the importance of optimising formulation viscosity and deposition to enhance brain delivery efficacy.

Zhang *et al.* performed a study focused on the IN administration of phenytoin (PHT) loaded layered double hydroxide NPs (BSA-LDHs-PHT) for treating epileptic seizures, aiming to enhance drug delivery efficiency to the brain while reducing peripheral toxicity 140. The delivery device used for this purpose was a pipette, administering a total volume of 50 µL per mouse. The BSA-LDHs-PHT bypassed the BBB by utilising the olfactory and trigeminal nerve pathways, enabling direct brain access from the nasal cavity. These NPs provided colloidal stability, controlled drug release, and enhanced brain targeting due to their positive charge and anion exchange properties. *In vivo* FLI tracked the distribution of BSA-LDHs-Cy5.5 in mice, revealing significantly higher brain/peripheral ratios, which was more than double the uptake for BSA-Cy5.5, thus indicating superior brain targeting. Fluorescence intensity measurements post-administration showed uptake values of 5.25 × 10^8^ at 15 min, maintaining high levels for up to 24 h. The highest brain concentration of phenytoin was 404.3 ng/mL at 4 h, remaining high for up to 8 h. Additionally, lower fluorescence intensities in peripheral organs (liver and kidneys) suggested reduced peripheral distribution and potential toxicity. The authors focused on overall brain uptake and specific regions, comparing peripheral distribution. The clearance of the tracer indicated prolonged retention in the brain, with high phenytoin concentrations maintained for 8 h post-administration. *In vivo* biodistribution assays, conducted at multiple time points (15 min to 24 h), confirmed sustained brain targeting and reduced peripheral distribution. This comprehensive study suggests that IN administration of BSA-LDHs-PHT is a promising strategy for improving the therapeutic effect of phenytoin in treating epileptic seizures with enhanced brain targeting and reduced peripheral toxicity.

A study by Chung and coworkers utilised both Cy5- and FITC-labelled small interfering RNA (siRNA) as an imaging agent to track the delivery, localisation, and clearance of siRNA complexes within the brain 141. The IN administration was performed using a pressurised olfactory device from Impel Neuropharma. These siRNA complexes consisted of anti-apoptotic siRNA targeting the Bax gene (Bcl-2-associated X protein), which were complexed with Fas-signaling blocking peptides (FBP) coupled with 9R a nona-arginine peptide, forming the FBP9R/siRNA complexes. Cy5-siRNA was primarily used to monitor the distribution and retention of the siRNA complexes in the brain. Following IN administration, the Cy5-siRNA complexes were observed to localise specifically to the ischemic region within 12 h, indicating rapid targeting. Significant retention of these complexes was noted up to 48 h post-administration, demonstrating effective targeting and prolonged presence in the ischemic area. FITC-siRNA was utilised to track siRNA uptake and intracellular localisation within cells. FITC labelling allowed for the precise visualisation of siRNA within cells using confocal microscopy and flow cytometry. This approach enabled the researchers to evaluate the efficiency of siRNA delivery by the FBP9R complexes to Fas-expressing cells. Confocal microscopy showed colocalisation of siRNA within endosomal vesicles, confirming efficient intracellular trafficking and endosomal escape. The FLI studies showed that the Cy5-siRNA complexes were predominantly taken up in the infarcted (ischemic) brain region, with minimal to no presence in non-ischemic regions. This specificity confirms the targeted delivery capability of the FBP9R/siRNA complexes. 9R with control peptides, however, demonstrated a rapid clearance profile, with a significant reduction in the imaging signal at 24 h post-administration. In contrast, FBP9R/siRNA complexes maintained a strong presence in the ischemic region for up to 48 h, suggesting slower clearance and better retention in targeted areas. Experimental findings from confocal microscopy and flow cytometry supported these observations. Confocal microscopy visualised the Cy5-siRNA in brain tissue sections, confirming targeted delivery and retention in the ischemic region, with specific localisation noted at 12 h post-administration. Flow cytometry quantified the presence of Cy5-siRNA in single-cell suspensions from brain regions, revealing a high percentage of Cy5-positive cells in the ischemic region, approximately 72% at 12 h, 42% at 24 h, and 40% at 48 h post-IN administration. Overall, findings highlight the potential of the IN-delivery system for achieving sustained therapeutic effects in targeted brain regions, with efficient delivery and prolonged retention of therapeutic agents. FITC and Cy5 labelling were crucial in providing detailed insights into the uptake mechanisms, regional localisation, and clearance profiles of the siRNA complexes.

A study by Tscherrig investigated the therapeutic potential of micro RNAs (miRNAs) carried by small extracellular vesicles derived from Wharton's jelly MSCs in a preclinical rat model of premature white matter injury 142. The primary focus was on the biodistribution, uptake mechanisms, targeted brain regions, and clearance of these small extracellular vesicles. Small extracellular vesicles were administered intranasally to ensure direct delivery to the brain. Fluorescent labelling with DiR dye enabled tracking of the small extracellular vesicles at 1 h, 6 h, and 24 h post-administration. The imaging results showed that small extracellular vesicles rapidly reached the brain, with significant accumulation observed as early as 1 h post-administration. The signals remained detectable for at least 24 h, indicating prolonged retention in the brain. Notably, higher uptake was observed in injured animals compared to controls, suggesting that injury might facilitate vesicle uptake due to increased permeability of the BBB. The study specifically targeted the corpus callosum and the external capsule regions of the brain. In the corpus callosum, significant reductions in microglial activation markers were observed following the administration of naïve small extracellular vesicles. This reduction highlights the anti-inflammatory effects of the small extracellular vesicles. Later, while strong signals persisted in the brain, there was notable clearance through the gastrointestinal tract, evidenced by strong fluorescent signals 24 h post-administration. This finding aligns with the known mucociliary clearance mechanisms from the nasal cavity. Moreover, no significant signals were detected in the liver, spleen, or lungs, indicating minimal systemic clearance through these organs. In summary, this is compelling evidence that intranasally administered Wharton's jelly MSCs small extracellular vesicles effectively target the brain, reduce inflammation and promote oligodendrocyte maturation in a model of premature white matter injury. The findings highlight the potential of miRNA-based therapies for treating neonatal brain injuries, with detailed imaging studies confirming the targeted delivery, retention, and clearance pathways of the small extracellular vesicles.

#### 2.3.2. Multimodal imaging

BLI and FLI imaging were crucial in tracking and evaluating the effectiveness of intranasal (IN) administration of polyfunctional gold-iron oxide NPs (polyGIONs) loaded with therapeutic miRNAs for treating glioblastoma 143. BLI, using FLUC-EGFP-labelled glioblastoma cells, enabled real-time, non-invasive monitoring of tumour growth and response to polyGION treatment. FLI, aided by Cy5 dye tagging of the miRNAs within the polyGIONs, provided detailed insights into the *in vivo* distribution and trafficking of these NPs. This approach revealed that T7-targeted polyGIONs effectively reached and accumulated in the brain, especially in the prefrontal cortex and other vital regions, highlighting their enhanced cellular uptake within glioblastoma sites. The uptake of polyGIONs was observed to initiate post-administration rapidly, with significant NPs accumulation in the brain within 24-48 h. The study indicated that the NPs remained detectable in the brain tissues at least up to 72 h post-administration, demonstrating a substantial retention period which is crucial for the therapeutic efficacy of the delivered miRNAs. This efficient delivery was facilitated by the mucoadhesive properties of the NPs, aiding their transit from the nasal cavity to the brain through perivascular spaces and along olfactory and trigeminal nerve pathways, thereby bypassing the BBB. Retention of the polyGIONs in the brain was observed for several days, indicating their stable presence at the target site. MRI played a vital role in visualising and tracking the NPs within the brain. The magnetic properties of the iron oxide component in the polyGIONs allowed for detailed observation of their spatial distribution, confirming their successful BBB bypass and direct delivery to brain tissue. The dynamic process of the NPs trafficking from the nasal cavity to targeted brain regions was mapped comprehensively, providing a complete view of the delivery mechanism, complemented by BLI and FLI imaging. In addition to real-time imaging, biodistribution analysis indicated significant retention of miRNAs in the brain, particularly in the prefrontal cortex and regions accessed via the olfactory bulbs and trigeminal nerves. This targeted accumulation demonstrated the potential of T7-targeted polyGIONs in enhancing the therapeutic effects of the chemotherapy drug temozolomide. The study highlighted the improved therapeutic outcomes with T7-targeted polyGIONs, which resulted in increased survival rates in glioblastoma-bearing mice compared to control groups receiving non-targeted NPs or temozolomide alone. The IN-administration device used in this study was specifically designed to facilitate the efficient administration of the nanoformulation. This device enabled the precise delivery of the polyGIONs into the nasal cavity, where their mucoadhesive chitosan coating allowed for prolonged residence time and enhanced uptake through the nasal mucosa. Overall, the use of multimodal imaging techniques, namely BLI, FLI and MRI, provided critical insights into the biodistribution, uptake mechanisms, and therapeutic efficacy of polyGIONs in glioblastoma treatment. This non-invasive, targeted delivery approach shows promise in improving the treatment of glioblastoma by effectively bypassing the BBB and enhancing the delivery and effectiveness of therapeutic agents like temozolomide.

#### 2.3.3. Remarks on OI

OI is revolutionising our understanding of INDD, particularly in the context of nose-to-brain pathways. For example, research using fluorescent QDs, such as carboxylated CdSe-ZnS QDs, in mice demonstrated their translocation across the olfactory epithelium, predominantly through extracellular routes, offering insights into brain imaging applications. Studies with Cy5.5 and CB systems revealed differential drug distribution patterns and efficiency in the brain based on the administration route, with IN administration showing significant potential for targeted brain delivery. Notably, research on AuNRs employed a combination of OI, gamma counting, and autoradiography, illustrating the comprehensive tracking of these particles' spatiotemporal dynamics in the brain. In studies investigating the rostral migratory stream and neurotoxicity of polystyrene NPs, OI highlighted crucial aspects of drug transport mechanisms and potential neurotoxic responses. Further, OI has been instrumental in evaluating the effectiveness of various therapeutic agents, such as insulin and icariin, and diagnostic agents like clathrin triskelia and fluorescent magnetic NPs, showcasing its versatility in enhancing our understanding of therapeutic agent delivery and distribution within the brain via IN administration. These advancements in OI highlight its potential in both therapeutic and diagnostic applications, offering essential insights into the mechanisms and efficiency of IN administration treatments, albeit with ongoing research needed to address safety and efficacy concerns.

OI's ability to provide real-time visualisation of the distribution and persistence of therapeutic agents and its non-invasive nature makes it an invaluable tool in neuroscience research, particularly in INDD studies. Its applications range from tracking drug movement to assessing the effectiveness of different formulations, creating an understanding of the mechanisms and efficiency of IN administration treatments. However, the field necessitates further research into the long-term effects and clearance mechanisms of various agents used in biomedical applications, addressing growing concerns about safety and efficacy.

### 2.4. Magnetic resonance imaging

MRI is capable of delivering high contrast between various soft tissues, facilitating the detection of a variety of diseases and the evaluation of therapeutic results 144. The imaging modality plays an important role in monitoring nose-to-brain drug delivery by providing real-time, non-invasive visualisation and tracking of the drug movement 145. This is usually achieved with the use of contrast agents incorporated into the drug delivery system 146.

MRI contrast agents normally involve metals, such as gadolinium (Gd), iron (Fe), and Mn. The presence of contrast agents with these metals is known to have a measurable impact on the relaxation times of tissue, albeit at sensitivities well below that afforded by PET and SPECT experiments. Normally, MRI-based studies evaluate contrast agent induced changes in T1 and T2 relaxation times. Commonly, Gd-based chelates, ultra-small superparamagnetic iron oxide (USPIO), micron-size paramagnetic iron oxide (MPIOs), superparamagnetic iron oxide NP (SPION), and Mn-based agents are used 147. Each contrast agent leads to a different type of soft tissue contrast enhancement, and they tend to be chosen specifically based on their characteristics.

#### 2.4.1. Gadolinium (Gd)

**Figure 9:**
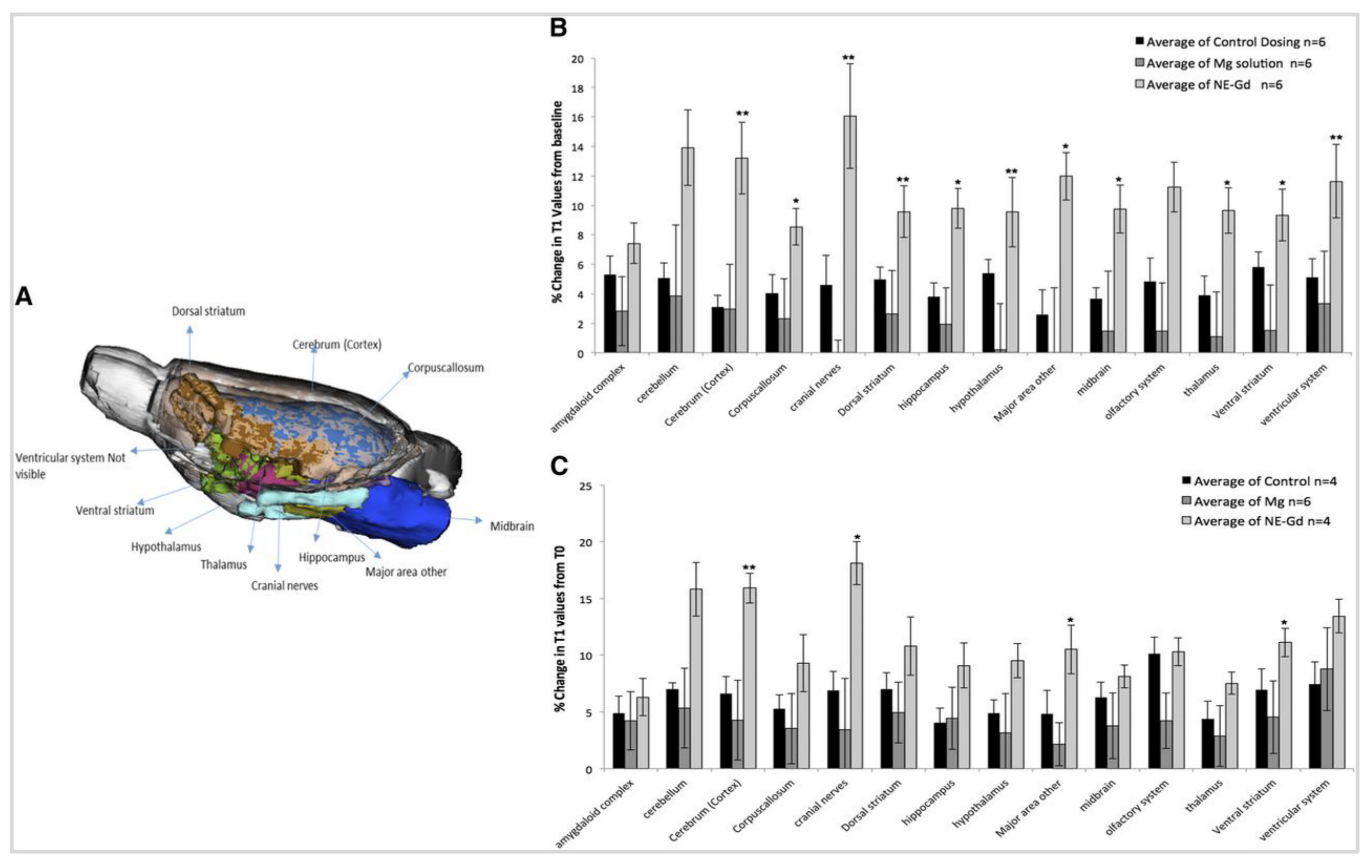
Demonstrate the Gd^3+^-NE distribution within the brain regions. An illustrative image highlights the spread of the Gd agent in various distinct areas of the rat brain map following IN administration (A). Gd^3+^-NE Distribution of in primary brain areas at 25-30 min post-administration, in contrast to Magnevist and the control (B). Distribution of Gd^3+-^NE in significant brain areas 55-60 min after administration, in comparison to both Magnevist and the control (C). Adapted with permission from 148 copyright 2019 American Society for Pharmacology and Experimental Therapeutics.

Numerous studies have explored nose-to-brain pathways using various imaging methods and agents. Upon IN administration using a micropipette, the Gd agent can access the brain in as short as 30 min post-administration 148. A study by Yadav *et al.* (2018) used MRI to evaluate and confirm the IN administration of positively charged cyclosporine A (CSA) NE (CSA-NE) to the CNS in rats. They compared the uptake of Gd-encapsulated NE (Gd-NE) and an aqueous solution of Gd-DTPA into the brain. MRI scans were acquired pre-administration, and 30, 60, and 90 min post-administration of the Gd agent to assess the change in T1 relaxation time in the rat brain (see Figure 9) 148. At 30 min, the decrease in T1 values were qualitatively notable in the main parts of the brain except for the amygdala and cerebellum. At 60 min, T1 changes became additionally evident in the cerebellum, cranial nerves, and ventral striatum. Also, their atlas-based comparison demonstrated a significant uptake of Gd-NE in 22 out of 172 brain regions. Moreover, the authors reported that Gd-NE had a longer residence time in the brain than Gd-DTPA. They concluded that the Gd contrast agent may be transported to the brain through both the trigeminal and olfactory pathways and diffuse from the olfactory bulb to other parts of the brain. The diffusion process may also be accelerated by the perivascular pump and arterial pulsation.

**Figure 10:**
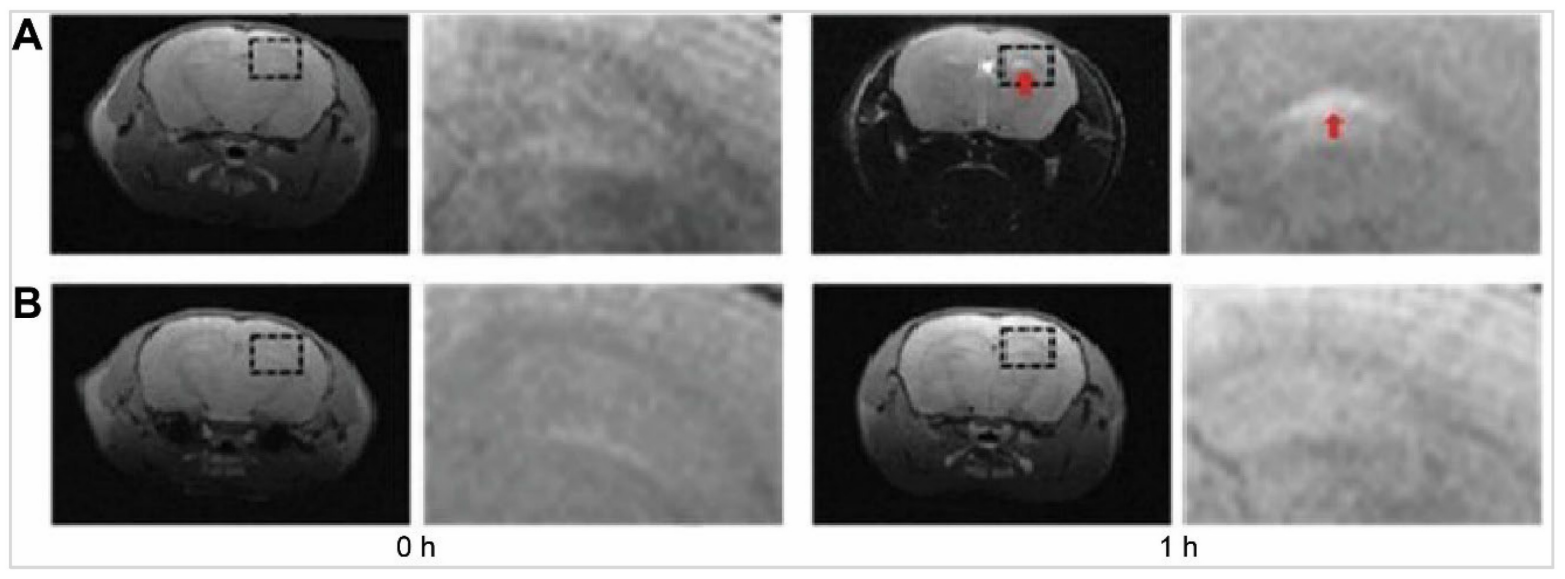
T1 MRI scans of mouse brains are displayed, taken at both 0 h and 1 h, before and after administration. The upper row (A) features images with CB-Gd-Cy5.5, while the lower row (B) presents those with BSA-Gd-Cy5.5. A red arrow highlights signal enhancement in the hippocampus. Adapted with permission from 118, copyright 2019 Wiley.

A Gd agent was used to evaluate IN administration of CB by micropipette in the mouse brain with the intended target being the hippocampus 118. Results were compared with those obtained using BSA. Scans were acquired before and 1 h post-administration of Gd-CB and Gd-BSA solutions. Signal enhancement using Gd-BSA was not reported; however, statistically significant MRI signal enhancement was found in the hippocampus using the Gd-CB agent (see Figure 10). This study confirms that Gd-based agents can be used to monitor potential pathways for INDD.

#### 2.4.2. Manganese (Mn^2+^)

Mn^2+^ is usually taken up by active neurons, which enables studies on brain function, neuron activity, and neuronal connections. Pautler *et al.* explored the potential of Mn^2+^ as a T1 contrast agent for MRI to trace neuronal pathways in live mice 149. The delivery devices used for administering Mn^2+^ were pipettes for the olfactory pathway and intravitreal injections for the visual pathway. MnCl_2_ solution was administered into the naris of the mice. The delivery pathways and mechanisms varied between the olfactory and visual systems. In the olfactory pathway, Mn^2+^ was topically applied to the naris, enabling its transport anterogradely from the olfactory receptor neurons through the olfactory bulb to the primary olfactory cortex. In the visual pathway, Mn^2+^ was injected into the vitreous humour of the eye and followed the optic nerve to the optic tract and superior colliculus, indicating trans-synaptic transport. This transport was evidenced by the enhancement of signals in specific brain regions associated with these pathways. In the olfactory pathway, signal enhancement was detectable 24 h post-administration, peaking around 29 h, and diminishing by 72 h. Enhanced regions included the olfactory bulb, primary olfactory cortex, and pituitary gland. In the visual pathway, enhanced contrast was visible within 2 h post-injection, peaking at 24 h, and returning to baseline by 72 h. Enhanced regions included the vitreous humour, optic nerve, and superior colliculus. Quantification of the Mn^2+^ tracer in the brain revealed that Mn^2+^ levels increased from 15 nmol/g tissue to 18 nmol/g tissue in the olfactory bulbs at peak signal enhancement. The most significant increase was observed in the microsomal fraction of subcellular structures, indicating effective uptake and transport of Mn^2+^ within the neurons. However, its use in humans is limited by its potential neurotoxicity.

In a different study a humidifier to aerosolise a concentrated MnCl_2_ solution, either alone or in combination with amyl acetate or male mouse urine, was used 150. This aerosolised solution was delivered to the mice in a controlled manner. The humidifier was turned on for 5 min and off for 5 min, repeated twice, and then kept off for 1.5 h before imaging. In another experiment, 4 µL of 39 µM MnCl_2_ in 1 ml H_2_O were pipetted into the mice's naris. Mn^2+^ was delivered intranasally, entering excitable cells through voltage-gated calcium channels. Once inside the cells, Mn^2+^ was transported anterogradely along neurons via a microtubule-dependent mechanism. This transportation led to the accumulation of Mn^2+^ in specific brain regions that were activated by the odour stimuli, such as the olfactory bulbs. The study demonstrated that Mn^2+^ could effectively trace neuronal pathways from the nasal epithelium to the olfactory bulb. Also, the study assessed the uptake time by imaging the mice 1.5 h post-exposure to the Mn^2+^ and odour stimuli. This timeframe allowed sufficient uptake and transport of Mn^2+^ to the brain regions of interest, enabling the researchers to capture the activated areas through MRI. Localised T1-weighted MRI enhancement was observed in both the olfactory epithelium and olfactory bulb. Specific regions of the olfactory bulb activated by the odours showed increased signal intensity. For the high pheromone content odour (male mouse urine), enhancement was seen in the accessory olfactory bulb, while amyl acetate caused enhancement in distinct regions of the olfactory bulb. The Mn^2+^ enhancement persisted long enough to allow for high-resolution MRI imaging, indicating that the tracer remained in the activated regions for a relatively extended period. This prolonged retention facilitated the detailed mapping of neuronal pathways. An occasional MRI enhancement in the ventricular regions was noted, suggesting systemic distribution and eventual clearance of Mn^2+^ through the brain's ventricular system and choroid plexus. This implies a possible route for tracer elimination from the brain.

Fa *et al.* investigated the use of IN MnCl_2_ administration for activity-induced Mn-dependent functional MRI of the rat visual cortex. MnCl_2_ was delivered intranasally using a pipette, with a total volume of 10 µl 151. The delivery pathway involved the transport of Mn^2+^ along the olfactory nerve, facilitated by olfactory and visual stimulation over a 20 h period, allowing Mn^2+^ to bypass the BBB. The transport mechanisms included neuronal uptake of Mn^2+^ through voltage-gated calcium channels, axonal transport, and diffusion in the extracellular space. Activation of neural pathways improved axonal transport, facilitating Mn^2+^ migration from the olfactory bulb to the visual cortex. This was confirmed by enhanced T1-weighted imaging in the visual cortex and deep brain structures, with specific enhancement noted in the intact olfactory bulb group. In contrast, the group with olfactory bulbectomy showed no cortical enhancement, highlighting the importance of the olfactory bulb in Mn transport. Quantification of the tracer reaching the brain was performed using inductively coupled plasma mass spectrometry. The results depicted elevated Mn levels in the visual cortex of the intact olfactory bulb group (7 µg/g) compared to the olfactory bulbectomy group (5 µg/g). This confirmed the significant transport of Mn from the olfactory bulb to the visual cortex in the presence of intact olfactory pathways. The elimination of Mn from the brain involved bioelimination processes, potentially including axonal transport, diffusion, and CSF shifts. The study suggested that Mn eliminated from the olfactory bulb could subsequently transfer to the visual cortex. This study confirmed that IN MnCl_2_ administration could effectively deliver Mn to higher brain regions, such as the visual cortex, enabling non-invasive functional imaging of brain activity.

Romashchenko *et al.* used MRI to explore the spatiotemporal dynamics and regulation of nose-to-brain transport of metal oxide NPs 152. Two types of NPs, Mn_3_O_4_ and Fe_3_O_4_, each with distinct imaging characteristics and distributions in the brain, were used. For Mn_3_O_4_-NPs, the delivery device was a micropipette, administering a volume of 10 µL intranasally. The delivery pathway involved the nasal cavity targeting the olfactory epithelium, where olfactory receptor neurons took up the NPs. These NPs localised within the cytosol, mitochondria, and vesicles of neurons, moving both intra- and extra-vascularly. The transport mechanisms were dependent on neuronal activity and microtubule integrity. The uptake of Mn_3_O_4_-NPs was rapid, with detection in the olfactory bulb shortly after administration. The NPs were trans-synaptically transmitted through at least four synapses across the olfactory tract, reaching regions such as the olfactory bulb and olfactory cortex. The retention time of Mn_3_O_4_-NPs in the brain varied, as influenced by neuronal activity and synaptic connections. Quantitative MRI analysis showed a substantial fraction of the administered dose reaching the brain, with accumulation rates correlated with neuronal activity. Eliminating Mn_3_O_4_-NPs from the brain was slower, primarily influenced by synaptic transmissions and neuronal activity, with gradual clearance observed. Similarly, Fe_3_O_4_-NPs were administered intranasally using a micropipette with the same volume of 10 µL. These NPs followed the same delivery pathway through the nasal cavity and olfactory epithelium, being engulfed by olfactory receptor neurons and transported axonally to the brain. The uptake of Fe_3_O_4_-NPs was rapid and detected in the olfactory bulb and other higher olfactory centres. However, the retention times for Fe_3_O_4_-NPs were shorter compared to Fe_3_O_4_-NPs, possibly due to different physicochemical properties affecting their transport and clearance. MRI data indicated substantial brain uptake of Fe_3_O_4_-NPs, though lower than Mn_3_O_4_-NPs. The elimination of Fe_3_O_4_-NPs from the brain was faster, with clearance mechanisms involving gradual removal from neuronal tissues. Moreover, aging and Parkinson's disease adversely affected their trans-synaptic movement. These findings open up new possibilities for diagnostic tools, functional neuroimaging, and targeted INDD.

The distribution of a mucoadhesive powder formulation containing MnCl_2_ was visualised in the nasal cavity of cynomolgus monkeys, particularly focusing on the olfactory region 153. The study explores the effectiveness of an INDD system targeting the olfactory region in cynomolgus monkeys. Three delivery devices were compared: the proprietary nose-to-brain system, an existing Abs-system, and a commercially available Liquid-system. The nose-to-brain system used a proprietary mucoadhesive powder formulation combined with a newly designed nasal device, while the Abs system utilised a Fit-lizer™ type A device, and the Liquid system employed a MAD Nasal™ intranasal mucosal atomisation device. The volumes administered varied across the systems, with the nose-to-brain system delivering 25 mg, 37.5 mg, 50 mg, and 75 mg of powder formulation, whereas the Abs-system and Liquid-system administered 25 mg of powder and 100 μL of liquid formulations, respectively. TR-Dex localised to the olfactory epithelium and reached the olfactory bulb through the cribriform foramina, while MnCl_2_ used in Mn-enhanced MRI helped visualise Mn^2+^ distribution primarily in the olfactory region and then to the olfactory bulb. The uptake time for the tracer was evaluated at intervals of 20 min, 3 h, 6 h, and 24 h post-IN administration. The nose-to-brain system showed significant Mn^2+^ signals in the olfactory region and the olfactory bulb, while TR-Dex was widely observed in the olfactory epithelial cell layer and lamina propria. The retention time for the nose-to-brain system demonstrated sustained Mn^2+^ signal intensity in the olfactory region for up to 24 h post-administration. The Abs-system also showed Mn^2+^ signal retention in the olfactory region, though the respiratory region saw a significant reduction in signal intensity after 24 h. In contrast, the Liquid-system showed no significant Mn^2+^ signal in the olfactory region, indicating rapid clearance of the formulation from the nasal cavity. Quantification of the drug/tracer reaching the brain revealed that the nose-to-brain system significantly increased domperidone uptake in D2R-expressing brain regions. The percentage receptor occupancy maps showed that the nose-to-brain system had the highest domperidone uptake compared to the Abs-system and Liquid-system. The elimination of the tracer from the brain was gradual, with Mn^2+^ signal intensity decreasing over 24 h. Histological analysis found the carrier to eventually be cleared from the olfactory mucosa within 6 h post-administration. High-resolution histochemical findings showed that TR-Dex delivered via the nose-to-brain system was extensively distributed in the olfactory epithelial cell layer and lamina propria, with immunostaining revealing its colocalisation with olfactory sensory neuron bundles and fibronectin-positive extracellular matrix. Comparative analysis of imaging agents showed that [^18^F]fallypride PET imaging demonstrated competitive inhibition in D2R regions, with the nose-to-brain system achieving significantly higher receptor occupancy and a dose-dependent increase in percentage receptor occupancy with higher domperidone dosages. Mn-enhanced MRI imaging confirmed higher distribution and retention in the olfactory region for the nose-to-brain system compared to the other systems. FLI of TR-Dex indicated extensive distribution in the olfactory epithelial cell layer and lamina propria when delivered by the nose-to-brain system. In conclusion, the nose-to-brain system showed superior efficiency in delivering formulations to the olfactory region, enhancing brain uptake, and maintaining retention in targeted brain regions compared to other nasal delivery systems. This study promotes the potential of the nose-to-brain system in achieving effective INDD, supported by high-resolution histochemical and imaging analyses. Here, fluorescence imaging was used to track TR-Dex, to validate the selective distribution and cellular uptake in the olfactory epithelium. These findings point towards a transcellular route via sustentacular cells and paracellular diffusion across the intercellular space as key mechanisms of drug transport in the olfactory region.

#### 2.4.3. Paramagnetic iron oxide

USPIO, SPIO and MPIO are contrast agents that can also be used to monitor INDD to the brain, kidney, spleen, and liver using MRI 154, 155. When conjugated with astrocyte-derived extracellular vesicles (ADEVs), USPIOs have a unique advantage as they do not alter the cellular physiology or morphology. Intranasally perfused USPIO-ADEVs bypass the BBB through the olfactory and trigeminal pathways, enabling fast and efficient delivery of the product to the CNS 154. This work investigated the sensitivity and detectability of USPIO-astrocytes and USPIO-ADEVs in a gel matrix. The researchers detected USPIO-ADEVs in the brain as early as one day post-perfusion over a four-day period. Furthermore, the quantity of USPIO-ADEVs increased with each subsequent administration. Additionally, the ADEVs were observed in other internal organs, including the kidneys, liver, and spleen, indicating a broad biodistribution. Transmission electron microscopy confirmed the presence of USPIO NPs within the astrocytes and the extracellular vesicles.

**Figure 11:**
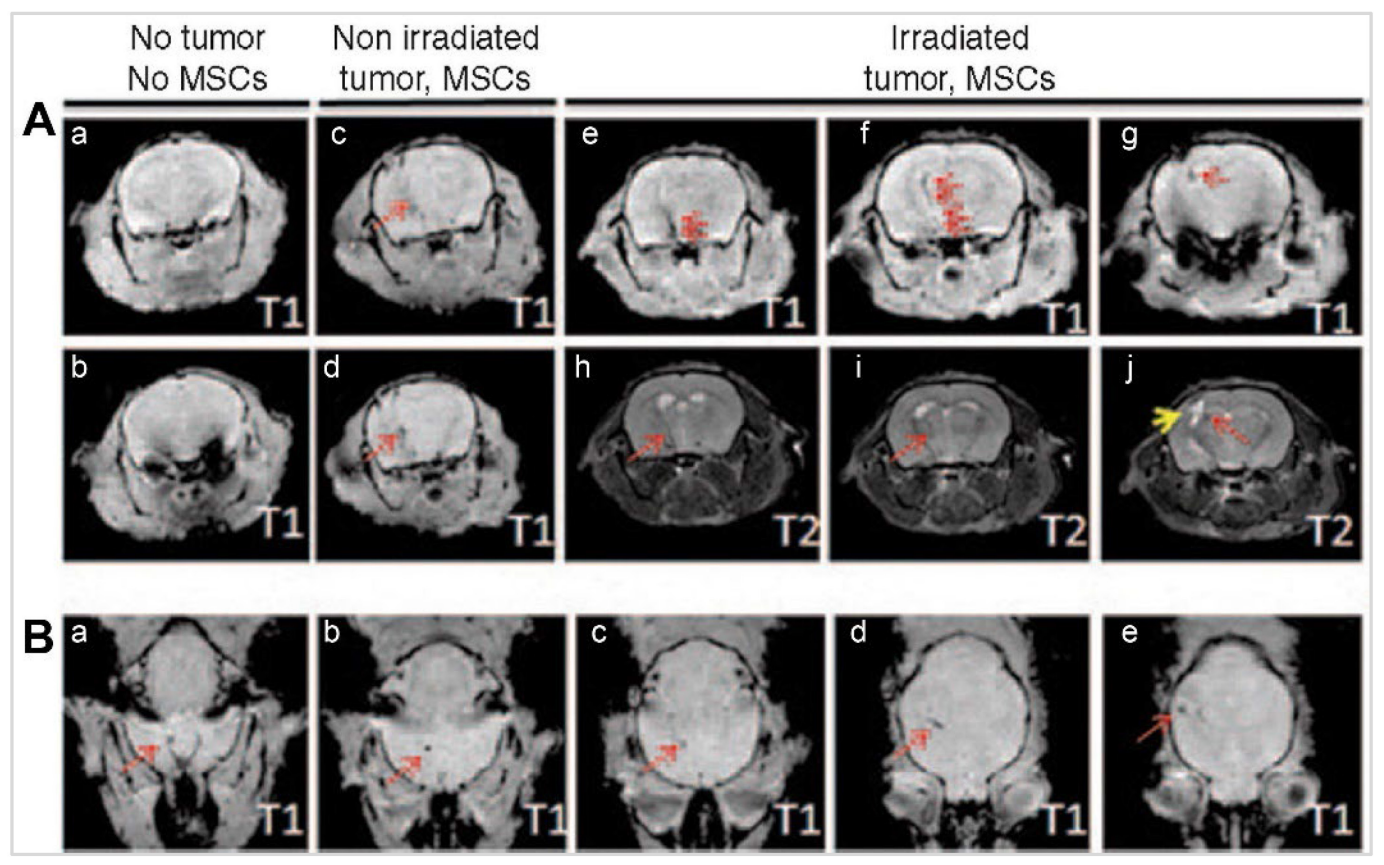
MRI images of MPIOs-MSCs migration in mice post-IN administration. Panel (A) presents coronal MRI images where images a and b serve as control showing absence of MSCs and tumour, thus no detectable signal. Images c and d show the migration pathway of MSCs in a nonirradiated, tumour-bearing mouse, while e through g display the migration in an irradiated mouse, all through T1-weighted imaging. T2-weighted images h, i, and j from an irradiated mouse highlight both the migrating MSCs, indicated by red arrows, and the tumour, pinpointed by a yellow arrow in j. Panel (B) illustrates axial T1-weighted brain images of an irradiated, tumour-bearing mouse from a to e, with red arrows denoting the direction of MSC movement. These images were captured on the second day following IN administration of MPIOs-MSCs, providing insight into the MSCs' behaviour and their potential therapeutic trajectory towards the tumour site. Adapted with permission from 156, copyright 2014 Elsevier.

The study utilised IN delivery to administer MSCs labelled with ^111^In-oxine and MPIOs to mice. The MSCs were observed to migrate through the cribriform plate into the brain tissue, driven by chemotactic signals from the tumour, particularly the CXCL12/CXCR4 axis, which was upregulated in irradiated tumour tissues 156. MPIOs-MSCs via IN administration can be transported into the brain through both the olfactory and trigeminal pathways. This facilitated the rapid migration of MSCs towards glioma xenografts. Within hours of administration, the MSCs began migrating towards the tumour sites, with approximately 11% of implanted MSCs detected in the tumour-bearing hemisphere as soon as 2 h post-inoculation. The presence of these cells in the brain was sustained, though significantly decreased by day 11. The MSCs primarily migrated towards the glioma xenografts. MRI and histological analyses demonstrated the presence of labelled MSCs in the basal region of the brain, moving sequentially towards the tumour site. The cells were detected in the olfactory bulbs and frontal lobes, indicating initial entry points and subsequent migration pathways. This migration was enhanced in irradiated tumours, with significant accumulation observed in these regions compared to non-irradiated controls. Balyasnikova *et al.* qualitatively tracked MPIOs-MSCs in a mouse model of glioblastoma. Initially in this research, they used several techniques to validate the viability of IN administration of MSCs to the brain in animals with tumours. Initially, they confirmed that MSCs can migrate within the brain towards tumours, consistent with previous studies 157-159. Using BLI to show that MSCs can move from the nasal cavity to the brain in mice. Notably, while the luciferase signal was immediately evident in both the nasal cavity and the brain post-IN administration, detecting MSCs in the nasal cavity at later times required longer exposure times, indicating a quick elimination of MSCs. This rapid disappearance was similarly noted in a Parkinson's disease rodent model 160. It appears that MSCs quickly travel from the nasal cavity to the brain, likely through direct pathways via the cribriform plate, involving the olfactory and trigeminal pathways. Further, the researcher observed that ^111^In-oxine-MSCs were detectable in the brains of mice. Radiation therapy in tumour-bearing animals seemed to increase MSC penetration into the brain. While MSC proliferation and clearance could vary between control and irradiated animals, these differences did not seem to influence the results observed 24 h after MSC delivery. Instead, it suggests enhanced brain penetration in irradiated animals. Importantly, they noticed a significant increase in the accumulation of ^111^In-MSCs in the cerebellum of irradiated animals, which is crucial considering the prevalence of cerebellar brain tumours in children. This emphasises the potential of IN administration of therapeutic stem cells in models of medulloblastoma and other similar tumours. The growing evidence that stem cells can penetrate from nose-to-brain highlights the importance of understanding this mechanism, which could potentially increase the number of stem cells reaching the brain. Lastly, the study introduced the use of MRI to track stem cells modified with MPIOs in the brain. Successfully detected signals from MPIOs-MSCs migrating towards the tumour in both control and irradiated animals following IN administration. This study is the first instance of using MRI to confirm the migration of MSCs delivered intranasally towards brain tumours 156. MRI images showed the migration path of MPIOs-MSCs toward brain tumours (Figure 11). The movement was monitored through sequential T1-weighted images for both non-irradiated and irradiated mice. The presence of tumours was identified on T2-weighted MRI images in irradiated mice. The MPIO signals, indicating the cells' location, were clearer in irradiated animals and could be seen moving towards the tumour from the basal region of the brain. Similar observations were made in axial MRI images, where the MPIO signals appeared as hypointensity spots. The authors observed an accumulation of MPIOs-MSCs in the brain and extending towards the tumour 48 h after IN administration. This was evidenced by signal reduction on T2-weighted images and hyperintensity on T1-weighted ones. These findings were further supported by the post-mortem analysis of brain tissue stained with Prussian blue, which confirmed the MSCs' presence at the tumour sites in both control and irradiated animals. This confirms the targeted movement of MSCs delivered intranasally towards tumours within the brain.

An SPIO used to monitor and track IN administration of NSCs through the olfactory epithelium towards inserted glioma xenografts 155. The delivery device was a micropipette. To optimise the delivery and migration of NSCs to the brain, the study employed methimazole to temporarily disrupt the olfactory epithelium. This pharmacological intervention delayed the clearance of NSCs from the nasal cavity, allowing for prolonged persistence and improved migration into the brain. BLI showed that NSCs were detectable in the nasal cavity within 30 min post-administration, with significant persistence observed at 6 and 24 h in methimazole-treated groups. MRI further confirmed enhanced accumulation of NSCs in tumour regions at 24, 48, and 120 h post-administration, demonstrating the effective penetration and retention facilitated by methimazole treatment. The team reported hypointensity and reduced T2 values, confirming the migration of SPIO to the brain. The most significant reduction in T2 values peaked at 120 h post-administration of the SPIO. These findings were validated against histology, confirming the presence of SPIO-NSCs within the tumour and its surroundings. The study identified the olfactory bulb and peri-tumoral brain regions as key uptake areas for the NSCs. Histological analyses using Prussian blue staining confirmed the presence of SPIO-NSCs in these regions, while confocal microscopy revealed increased GFP signals in the tumour areas, indicating successful migration and retention of NSCs in methimazole-treated animals. Additionally, hexon protein staining showed a significantly higher viral load in glioma tissues after NSCs-oncolytic virus treatment in methimazole-treated animals, highlighting the improved delivery of the therapeutic payload. Overall, the study demonstrated that methimazole pretreatment effectively enhances the IN delivery of NSCs to brain tumours by disrupting the olfactory epithelium, thereby delaying NSC clearance and facilitating their migration and retention in glioma tissues. This approach holds promise for improving the therapeutic efficacy of non-invasive treatments for malignant brain tumours, offering a potential platform for future clinical applications.

Chastkofsky *et al.* demonstrated that upon IN administration, these SPIOs transport through the trigeminal pathway to the xenografts in the brainstem 161. The study used a patient-derived xenograft model of diffuse intrinsic pontine glioma in nude mice. MRI was employed to trace and quantify SPIO-MSCs following IN delivery. The MSCs were administered intranasally and observed to migrate along the trigeminal nerve towards the diffuse intrinsic pontine glioma xenografts located in the brainstem. MRI imaging, which tracked the MSC's movement, confirmed this pathway. The MSCs were found to migrate to the tumour within 48 h post-IN, with the most concentrated hypointense signals detected in the tumour region at this time point, indicating that MSCs reach the tumour site within 2 days. At later time points (5 days post-IN), a more diffuse hypointense signal was observed in the brainstem. The MSC's presence around the tumour was further confirmed by Prussian Blue staining and immunofluorescence. Immunofluorescence with antibodies specific for CD105 (a marker for MSCs) and H3-K27M (a mutation-specific for the diffuse intrinsic pontine glioma xenograft) confirmed the presence of MSCs around the tumour 5 days post-IN. These findings supported the MRI results, confirming the localisation and migration of MSCs to the tumour site.

In a different study, SPIO was used to monitor IN administration of MSCs to asses stem cell therapies for brain injuries and diffuse intrinsic pontine glioma 162. The objective was to evaluate the feasibility and efficacy of this method in a traumatic brain injury mouse model using MRI. The MSCs-SPIO NPs tagged with FITC and delivered intranasally using a pipette. The total volume of 18 µL was administered. The SPIO NPs served as the imaging agents, enabling the non-invasive tracking of the labelled MSCs via MRI. T2*-weighted MRI was employed for its sensitivity in detecting the hypointense areas indicative of SPIO presence. This method allowed real-time visualisation and longitudinal tracking of the MSCs as they migrated toward the site of cortical injury. The study observed that SPIO-MSCs were detectable as strong hypointense areas on T2*-weighted images as early as 24 h post-delivery. These areas were predominantly located medial to the cortical injury, suggesting targeted migration of the MSCs. Importantly, these hypointense signals persisted up to 14 days post-delivery, confirming prolonged retention of the labelled cells at the injury site. While the study successfully visualised the presence of SPIO-MSCs at the injury site via MRI, it did not provide specific quantitative data on the exact percentage of the administered cells that reached the brain. The focus was more on demonstrating the feasibility and tracking capability rather than precise quantification. To corroborate the MRI findings, high-resolution histochemical analysis was performed. Prussian blue staining confirmed the presence of SPIO-MSCs in brain tissue sections, revealing blue-coloured iron deposits in the labelled cells. Additionally, FITC fluorescence microscopy further validated these findings by showing FITC-tagged SPIO-positive cells in the injured cortex, correlating well with the MRI results. These histochemical techniques provided robust evidence supporting the non-invasive imaging findings. The study highlighted the effectiveness of using T2*-weighted MRI for tracking SPIO-MSCs, noting its sensitivity and accurate association with histological findings. This method provided clear and reliable data on the biodistribution and migration of the MSCs within the brain.

Simorgh *et al.* conjugated olfactory ecto-MSCs with Alginate (Alg)-coated SPIOs to target the striatum in the brain 163. The cells utilised both the olfactory and trigeminal nerve pathways to reach the CNS. The magnetic targeting cell delivery method significantly enhanced this process. By using magnetic forces to direct and concentrate the cells to the desired brain regions, the approach minimised cell loss during transit and increased the efficiency of cell delivery. The MRI scans were pivotal in tracing the perfusion of Alg-SPIO olfactory ecto-MSCs. Images captured before and two days after cell infusion revealed significant findings, indicating that the initial uptake and detectable presence of the cells in the brain occurred within this timeframe. The primary uptake region was the striatum, a critical area affected by Parkinson's disease. This was confirmed through MRI imaging, which showed a reduction in signal intensity in the striatum region, indicating the presence and concentration of the labelled stem cells. The study also demonstrated long-term retention of the stem cells. MRI evaluations showed the presence of labelled cells in the brain up to eight weeks post-administration. Histological analyses, including Nissl staining, confirmed the survival of dopaminergic neurons in the substantia nigra for this duration, showcasing the effectiveness of the stem cell integration. The use of magnetic forces significantly increased the number of cells that reached the lesion site. As such, this study highlights the potential of combining IN administration with magnetic targeting for delivering stem cells to the brain. The approach shows great promise for treating Parkinson's disease by ensuring efficient delivery, substantial retention, and functional integration of stem cells in the affected brain regions. The findings, confirmed by MRI and histochemical analyses, demonstrate the effectiveness of this novel therapeutic strategy.

In Kou *et al.*'s study on IN administration of NPs for CNS delivery, PEGylated ultrasmall Fe_3_O_4_ NPs served as a dual-modality tracer, utilising both MRI and fluorescence imaging 164. These NPs, designed to enhance MRI contrast and tagged with the fluorescent dye (Cy5.5), were monitored to understand their distribution and eventual elimination in the rat brain. The MRI data highlighted a dynamic distribution of the NPs, particularly in the olfactory bulb, pons, and cerebellum, revealing temporal variations in their uptake. Notably, the olfactory bulb showed increased MRI signals as early as 5 min post-administration, while the pons and cerebellum exhibited similar increases around 15 min post-administration. This variation in timing suggests different pathways and rates of NP penetration into various brain regions. Complementing the MRI data, fluorescence imaging provided a more detailed view at the tissue level, confirming that the trigeminal and olfactory nerves are significant pathways for the NPs to reach the CNS. The study showed that the NPs primarily travelled along these nerves to enter the brain, reaching the subarachnoid space and CSF. Once in the CSF, the NPs circulated throughout the cerebral cistern, allowing them to spread to other brain regions. The elimination of the NPs from the brain followed the CSF circulation, eventually being excreted via the arachnoid villus and superior sagittal sinus into the venous system. The maximum accumulation of Fe_3_O_4_ NPs in the brain was 5.97 μg/g at 1 h post-administration, indicating a significant initial uptake followed by clearance within a few hours. High-resolution histochemical findings were confirmed through fluorescence microscopy and Prussian blue staining. Fluorescence microscopy of brain slices showed that the NPs were mainly accumulated in the meninges. Prussian blue staining validated the presence of Fe_3_O_4_ NPs in the connective tissues of the trigeminal nerve and brain. No intracellular transport within the axons of olfactory neurons was observed, indicating paracellular transport through the nerve sheaths. Notably, the NPs traverse through the perineural space, ultimately infiltrating the brain parenchyma, and provide valuable insights into effective CNS drug delivery mechanisms, potentially enhancing treatment strategies for CNS diseases.

Law and colleagues (2023) developed a CT contrast agent using iohexol-loaded liposomes with >10% PEG to monitor the nose-to-brain tract using chemical exchange saturation transfer (CEST) MRI 165. The choice of iohexol was due to its ability to produce a distinct CEST contrast at 4.3 ppm on the Z-spectrum and its single CEST property, preventing readout overlaps when tracking multiple components of the *in vivo* delivery system. Which was detectable by CEST MRI due to specific amide protons. *In vitro*, these liposomes generated significant CEST contrasts at 4.3 ppm, 3.4 ppm, and 1.2 ppm. After IN administration, signal increases in CEST contrast were observed in the olfactory bulb and frontal lobe with the 10% PEG formulation, especially prominent at 4.3 ppm. Contrarily, the 1% PEG formulation showed no notable increase. The brain showed a 62% increase in CEST at 4.3 ppm in the olfactory bulb and a 40% increase in the frontal lobe. Additionally, CEST contrast at -3.4 ppm indicated a 11% increase in the olfactory bulb and 9% in the frontal lobe. The estimated dose of iohexol delivered from the olfactory bulb to the frontal lobe was 57%, while 87% of the liposomes were delivered, indicating partial release of iohexol from the liposomes. Elimination of the tracer involved partial release of iohexol, with approximately 36% released within the first hour. It was suggested that elimination likely occurs through the CSF and systemic absorption. Histochemical findings validated the MRI results, with a high rhodamine signal observed in the olfactory bulb and frontal lobe of mice treated with 10% PEG iohexol liposomes. This signal was significantly higher than in mice treated with 1% PEG iohexol liposomes, supporting the MRI findings of higher uptake and retention with the 10% PEG formulation. The histological analysis confirmed the distribution of liposomes in specific brain regions, particularly in CSF-rich areas like the external plexiform layer and the region between the left and right olfactory bulbs. The study concluded that the multiple CEST contrast approach is effective for non-invasively detecting and monitoring the delivery of liposome-based drugs from the nose to the brain. The findings emphasise the importance of PEG coating and particle size in overcoming nasal mucus barriers and achieving significant brain uptake. This approach provides valuable insights for optimising the INDD, highlighting the potential of CEST MRI for guiding the design and monitoring of such systems. The distinctive CEST MRI contrasts can be used to aid in non-invasively tracking the nose-to-brain delivery of liposomes.

A study by Seino *et al.* examined the efficacy of IN administration of SPIO as a contrast agent for magnetic particle imaging 166. They focused on enhancing the delivery and targeting of these NPs to specific brain regions. SPIOs were modified by compositing them with AuNPs, facilitating additional surface modification via gold-sulfur bonds without compromising their magnetic properties. PEG was used to coat the NPs to prevent aggregation and nonspecific adsorption. The NPs were further functionalised with tracer molecules called ABC 595 designed to target Aβ. The resulting tracer, ABC-PEG-Au/FcM, consisted of Ferucarbotran Magnetic (FcM), AuNPs, PEG coating, and the ABC 595 probe molecule. *In vivo* experiments involved administering these PEGylated magnetic NPs intranasally to mouse models. The study employed superconducting quantum interference device measurements to track the migration and accumulation of NPs in the brain. Results showed that approximately 0.3-0.6% of ID successfully reached the brain. The NPs migrate through the nasal mucosa to the olfactory bulb, subsequently accumulating at the target site Aβ plaques, confirmed through fluorescence microscopy. This selective targeting is significant for imaging applications in Alzheimer's disease. Additionally, these NPs maintained their magnetic properties and did not ionise during migration. Over time, the NPs detached from the target sites, suggesting a mechanism for their clearance from the brain, which is beneficial from a safety perspective. The study concluded that IN administration is an effective method for delivering magnetic NPs into the brain, providing a non-invasive alternative to bypass the BBB. These NPs also retain their magnetic properties upon delivery and accumulate at target sites, offering promise for early diagnosis and monitoring of neurological conditions.

#### 2.4.4. Remarks on MRI

MRI has become a crucial imaging modality in neuroscience, particularly in monitoring INDD. Its ability to differentiate between various soft tissues with high contrast makes it an invaluable tool for detecting diseases and evaluating therapeutic outcomes. In the context of nose-to-brain pathways, MRI provides real-time, non-invasive visualisation and tracking of drug movement, typically achieved using contrast agents involving metals.

The contrast agents, such as Gd-based chelates, USPIO, MPIOs, SPIONs, and Mn-based agents, significantly affect the relaxation times of tissues, altering T1 and T2 relaxation times for enhanced imaging. For instance, studies have shown that Gd agents can access the brain rapidly post-IN administration, with changes in T1 relaxation times observable within 30 min. A notable study by Yadav *et al.* compared the uptake of Gd encapsulated NE (Gd-NE) and aqueous solution of Gd-DTPA in the rat brain, revealing significant uptake in various brain regions and suggesting transport through both trigeminal and olfactory pathways.

MRI has also been instrumental in tracking Mn^2+^-based agents, typically taken up by active neurons, allowing for studies on brain function and neuronal connections. However, neurotoxicity concerns around the use of Mn^2+^ have led to the investigation of Mn^2+^-based NPs and chelates. These agents, when given intranasally, show dynamic and quantitative data on trans-synaptic translocation and nose-to-brain migration, with visual stimulation enhancing the transport of Mn^2+^ from the olfactory bulb to the visual cortex.

In addition to what may be considered as traditional MRI contrast agents, studies have also utilised USPIO, SPIO, and MPIO. For example, USPIO conjugated with astrocyte-derived extracellular vesicles demonstrated the ability to bypass the BBB through the olfactory and trigeminal pathways, leading to efficient CNS delivery. Similarly, MPIOs have been used to track the IN administration of MSCs to the brain, highlighting the potential of this route for drug delivery to specific brain regions.

These MRI studies offer clear insights into the dynamics of drug transport and distribution in the brain following IN administration. The ability to visualise and track these processes in real time has significant implications for the development of targeted therapies for CNS disorders, providing a better understanding of the potential and limitations of INDD.

### 2.5. Focused ultrasound

Research using FUS in combination with IN administration has made significant strides over the last decade. FUS is a non-invasive therapeutic technology that uses frequencies above human hearing and up to 18 Mhz to target tissue deep in the body without incisions or radiation. Its technique has been gaining traction as a potential tool for enhancing INDD. FUS leverages the mechanical and thermal effects produced by US waves to transiently open the BBB, enabling targeted delivery of therapeutic agents 167-169. To ensure the effective and safe translation of US technologies in drug delivery to clinical applications, Isaac's theranostics review outlines crucial guidelines for clinical translation 170.

#### 2.5.1. FUS and microbubbles

FUS was primarily spearheaded by Chen *et al.* and Ye *et al.* conducted studies 171-173. The method entails the IV delivery of microbubbles succeeded by the utilisation of FUS to induce an opening of the BBB 168, 169. The process of FUS involves the external transmission of US pulses through the skull, which are then focused onto a small area, usually measuring a few millimetres, located in the subcortical structures 174. In their seminal 2014 and 2016 studies, Chen H *et al.* developed an innovative strategy that leverages FUS to enhance the delivery of IN administration drugs to specific brain regions. They provided proof-of-concept for delivering a model drug (Dex) and brain-derived neurotrophic factor (BNDF) to the caudate putamen of mouse brains. Their approach achieved drug delivery efficiency comparable to the nose-to-brain alone, indicating that FUS could potentially enhance the nose-to-brain delivery through a process of active pumping 171, 172.

#### 2.5.2. Gold nanoclusters

**Figure 12:**
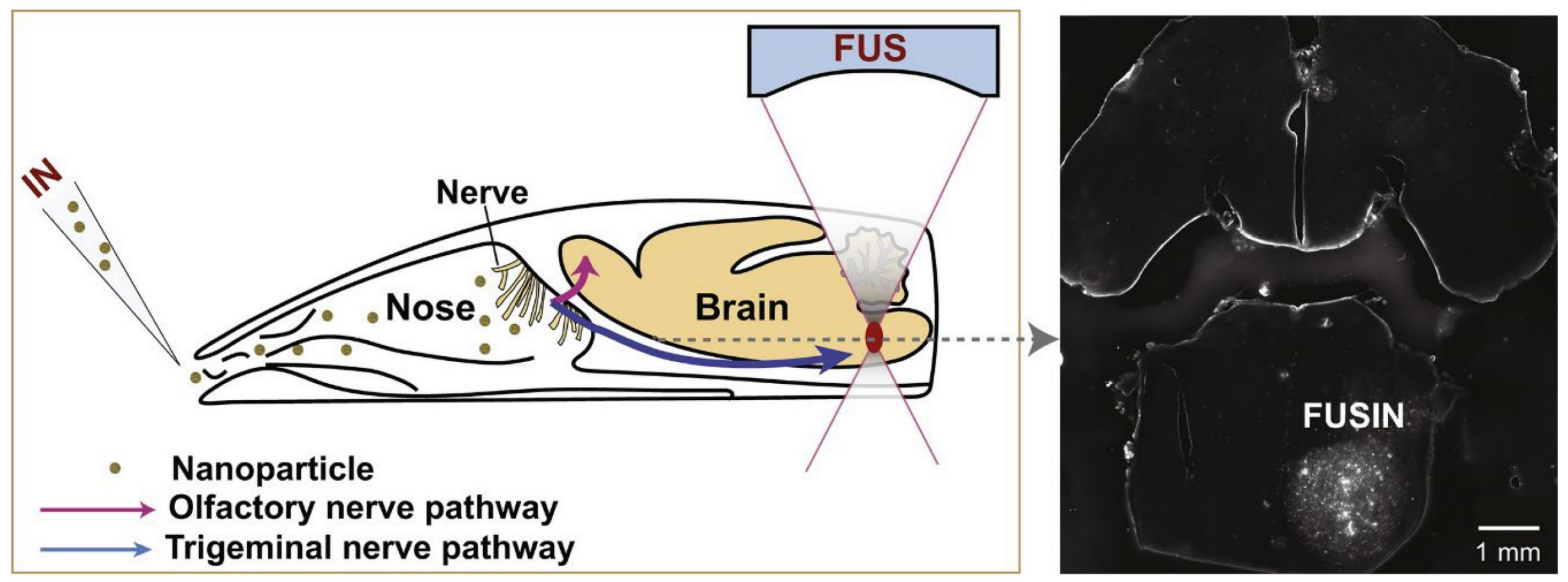
Demonstrating FUS+IN administration of NP and the potential pathways from nose-to-brain. "Reproduced with permission from 173, copyright 2018 ELSEVIER".

In 2018, a study by Ye *et al.* used FUS combined with microbubble-mediated IN delivery (FUS+IN) to administer gold nanoclusters (AuNCs) to the brain 173, as shown in Figure 12. The IN-delivery device involved administering 24 µL of AuNCs. The AuNCs used in the study had a hydrodynamic size of 5.60 ± 1.50 nm. The delivery pathway for the AuNCs was through the nasal route, specifically targeting the olfactory and trigeminal nerve pathways. This approach led to the NPs bypassing the BBB and directly entering the brain. FUS-induced microbubble cavitation further enhanced the transport of these NPs to the targeted brain regions. This mechanism, referred to as the "microbubble pump effect," is similar to the perivascular pump effect and enhances the distribution of intranasally administered agents to the FUS-targeted brain location. The uptake time of the AuNCs was evaluated using various imaging techniques. PET/CT imaging and *ex vivo* gamma counting were performed 1 h post-administration to assess the biodistribution of [^64^Cu]AuNCs. The data indicated that the amount of radioactive tracer was >10-fold higher in the blood, lungs, liver, spleen, kidneys, and heart when IV injection was used, compared to IN administration. The study found significant fluorescence in the brainstem at 1 h post-administration, indicating successful uptake of the NPs. The primary uptake region identified was the brainstem, specifically the left side targeted by FUS. Comparisons were made between the FUS-treated side and the contralateral side to evaluate the effectiveness of the technique. The retention time of the NPs was studied as well. Enhanced fluorescence signals in the trigeminal nerve were observed 1 h post-administration, which significantly diminished by 24 h, suggesting the clearance of the NPs from the nerve. This rapid clearance indicated that the NPs did not remain in the nerve tissue for extended periods, reducing potential toxicity risks. The study used two types of imaging agents: [^64^Cu]AuNCs and TR-labelled AuNCs (TR-AuNCs). The distribution of these agents was evaluated using multiple imaging modalities. PET/CT imaging provided whole-body biodistribution data, autoradiography assessed the spatial distribution of [^64^Cu]AuNCs in brain slices, and fluorescence microscopy examined the distribution of TR-AuNCs in the brainstem. The findings revealed a significantly higher accumulation of both [^64^Cu]AuNCs and TR-AuNCs in the FUS-targeted brain regions compared to the non-targeted regions, demonstrating the effectiveness of FUS in enhancing localised delivery. Quantification of the drug/tracer reaching the brain showed that FUS+IN significantly increased the delivery efficiency. The radioactivity of [^64^Cu]AuNCs in the FUS-targeted brainstem was 2.72 times higher than IN delivery alone. Similarly, the fluorescence intensity of TR-AuNCs in the FUS-targeted region was 2.32 times higher than in the non-targeted side. These results indicated that FUSIN effectively enhanced the localised delivery of NPs to the brain. The elimination of [^64^Cu]-AuNCs was characterised by minimal presence in the blood and major organs after IN administration. That is, the NPs were predominantly cleared via mucosal removal to the nasopharynx and subsequent accumulation in the stomach and intestines for fecal excretion. Fluorescence signals from TR-AuNCs in the trigeminal nerve diminished by 24 h, indicating effective clearance from the nerve tissue. Histochemical analysis was conducted to assess any potential tissue damage resulting from the FUS+IN treatment. The study found no histological-level tissue damage in the nose, trigeminal nerve, or brain. Hematoxylin and eosin staining of nasal tissue and LFB-CV staining of the trigeminal nerve showed no changes compared to the control groups. These findings highlight the short-term safety of FUS+IN, with no observed histological damage following treatment, and the study supports the potential of FUS+IN as a promising technique for non-invasive, spatially targeted, and safe delivery of NPs to the brain with minimal systemic exposure. The combination of PET/CT, autoradiography, and fluorescence microscopy provided comprehensive insights into the distribution, retention, and clearance of the NPs in the brain. Furthermore, the study aligns with previous research findings that IN administration delivery of larger NPs, such as micelles with a diameter of approximately 600 nm, is associated with minimised systemic exposure 175. This study extends understanding on NP biodistribution by demonstrating that small-size AuNCs share with larger size NPs the advantage of minimising systemic exposure. This comparison on the versatility of NP size in achieving reduced systemic toxicity through targeted delivery methods offers valuable insights into the design of safer nanotherapeutics. Notably, this appears to be the only study employing [^64^Cu] in the context of nose-to-brain. Future research should continue to validate these findings through direct quantification methods and long-term safety evaluations, including histochemical analyses to confirm imaging results. This group further expanded the potential applications of FUS+IN administration in two separate studies. They explored the effectiveness of the method in concentrating the delivery of anti-programmed cell death ligand 1 (aPD-L1) antibody to the brain labelled with near-infrared fluorescent dye (IR800), and in facilitating adeno-associated viral delivery (AAV5) labelled with EGFP to the brain. Both studies yielded highly encouraging results, reaffirming the potential of FUS-assisted nose-to-brain delivery as a versatile, efficient method for targeted drug delivery within the CNS 176, 177.

#### 2.5.3. Remarks on FUS

The mechanism of FUS+IN administration is indeed an active area of research. The nose-to-brain delivery method utilises the olfactory and trigeminal nerves ending in the nasal cavity as direct routes for medication delivery into the brain. The most likely transport mechanism from the nose-to-brain is bulk transport along the channels surrounding these nerves, a conclusion drawn from the rapid speed of transport observed. After reaching brain entry points, the IN administration agents are then distributed throughout the brain along the cerebral perivascular spaces. This distribution is aided by the perivascular pump effect, where the heartbeat-driven pulsations of the blood vessel wall drive the agents through these spaces. This innovative approach combines the precision of FUS in opening the BBB and the direct drug delivery advantages of the IN administration method. The existing series of studies have opened new frontiers in non-invasive therapeutics and hold value for the treatment of various CNS disorders having localised distributional characteristics.

### 2.6. Computed tomography

CT provides detailed anatomical pictures by combining X-ray technology and tomographic image reconstruction. Despite its high-resolution imaging capability, CT's role in the nose-to-brain pathway is relatively limited due to the scarce contrast agents available and its low sensitivity and lack of soft tissue contrast 178. However, its value significantly increases when combined with modalities such as PET or SPECT, offering a more comprehensive understanding of nose-to-brain applications. The CT scan supplies detailed anatomical images for reference, while the PET or SPECT scan provides dynamic information. This combination can offer complementary insights into the contrast agent's delivery process and its distribution profile 178.

#### 2.6.1. Gold nanoparticles

The application of AuNPs as contrast agents in CT imaging has been explored in the INDD pathway primarily because AuNPs enhance the visibility of the administered drugs and their biodistribution. In 2018, Betzer *et al.* addressed a key challenge in exosome research tracking these vesicles *in vivo* 179. They labelled exosomes derived from MSCs with AuNPs and successfully used *in vivo* micro-CT imaging to track the labelled exosomes in an animal model of acute striatal stroke. They found an accumulation of the exosomes in the murine model's brain within 1 h of administration, with a concentration at the stroke site apparent after 3 h. These exosomes remained at the lesion site for up to 24 h, while in contrast, the control group showed no specific exosome accumulation and clearance from the brain began after 24 h. These findings suggest AuNPs could serve as effective markers for tracking drug distribution and have potential for use in brain theranostics.

**Figure 13:**
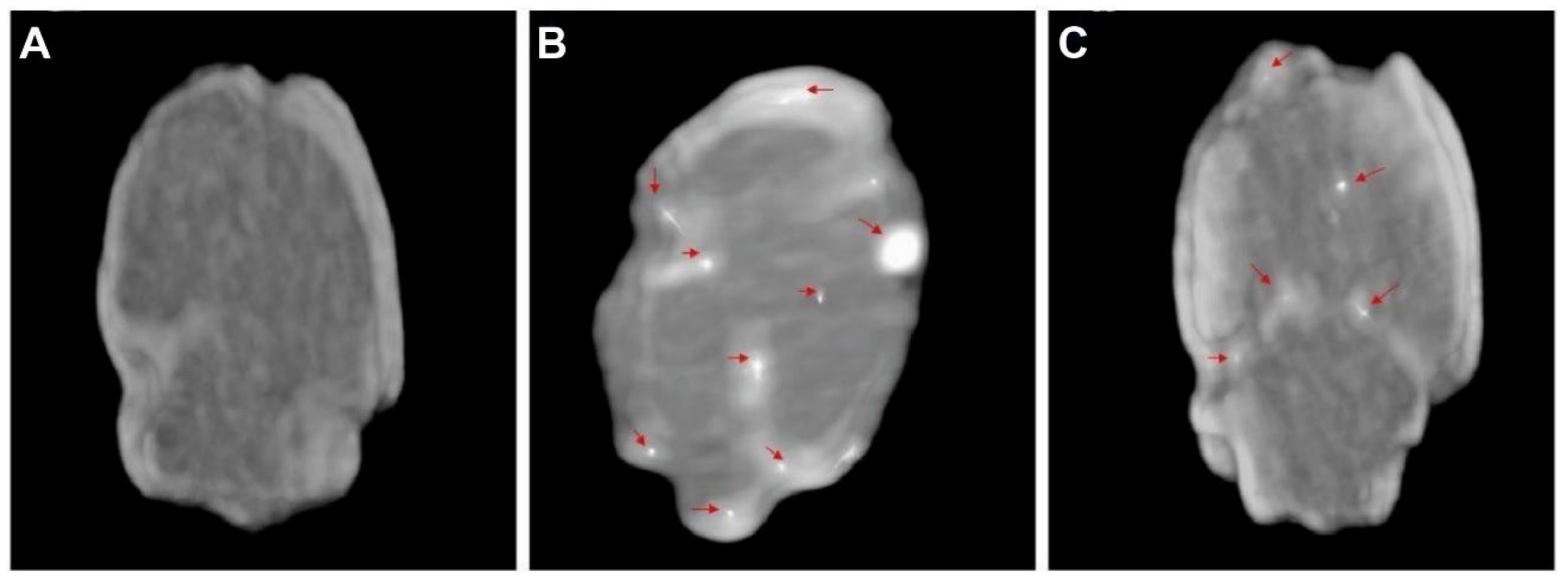
Demonstrates CT images depicting the uptake of AuNPs in the brain. The images compare three scenarios: (A) an untreated rat, (B) a resveratrol transferosome capped with AuNPs treated rat, and (C) a resveratrol NE AuNPs treated rat. The red arrow points to the observed uptake of AuNPs in the brain. Adapted with permission from 180, copyright 2019 Taylor & Francis.

In 2019, Salem *et al.* expanded on this work by investigating brain-targeted resveratrol delivery via nose-to-brain pathway lipid vesicles labelled with AuNPs 180. The AuNPs enabled validation of the cellular uptake of the formulated nanomaterials in the brain. Particularly, transferosomes displayed superior permeation rates and a higher fluorescence intensity compared to NEs, thereby demonstrating a significant improvement in behaviour acquisition and spatial memory function in amnesic rats. The authors successfully demonstrated AuNP accumulation in the brains of all CT-treated rats (see Figure 13). By comparing the CT images, they could distinctly identify bright regions in the brain, reflecting the accumulation of AuNPs (indicated by red arrows). These brightly illuminated areas are distinguishable from the surrounding tissues due to the X-ray attenuation induced by the AuNPs. CT images could distinguish specific bright cerebral areas that represent the accumulation of AuNPs.

Later, Bekhet *et al.* formulated citicoline-loaded niosomes that were labelled with AuNPs with the goal of improving the management of epilepsy disorders 181. They used CT imaging to examine brain uptake and cellular translocation of this formulation. The results of the study indicated that a low dosage of IN administration citicoline-loaded niosomes in a soluble gel had a protective effect, significantly extending the latency period before the onset of convulsions. Importantly, the CT images displayed high attenuation of the X-ray beam in different brain regions to indicate AuNP accumulation. The bright patches on the CT scans suggest that IN administration of the AuNP-labelled citicoline-loaded niosomes effectively bypassed the BBB and reached various regions of the brain.

#### 2.6.2. Remarks on CT

Collectively, studies using CT underscore the potential of AuNPs as contrast agents in the INDD pathway. By integrating AuNPs with various delivery vehicles such as exosomes, transferosomes, and niosomes, the targeted delivery of therapeutics can be traced and validated. AuNPs can, by bypassing the BBB, reach various regions of the brain effectively. This approach not only enhances the efficacy of therapeutic agents, but also provides a powerful tool for real-time tracking and verification of drug delivery through CT imaging. As such, AuNPs promises to be an indispensable asset in optimising the nose-to-brain pathway, boosting the potential of IN administration for improved disease diagnosis, monitoring, and therapy. However, the exact time it takes for AuNPs to reach these regions, their retention time in the brain, and their distribution across the brain can vary significantly and depend on multiple factors. These factors include the specific formulation used (e.g., exosomes, transferosomes, or niosomes), the disease model, and the specific imaging technique used. As for whether AuNPs can provide full brain coverage in nose-to-brain delivery, more research is needed to form a conclusive view. Given the complex and heterogeneous nature of the brain, it may be challenging for any single delivery system to achieve complete brain coverage. However, AuNPs certainly show promise in enhancing the reach and efficacy of therapeutically active agents in various brain regions. Further research is necessary to fully understand the kinetics, biodistribution, and clearance of AuNPs in nose-to-brain delivery and to optimise their use for different therapeutic agents and disease states.

## 3. Methods

### 3.1. Literature search methods

**Figure 14:**
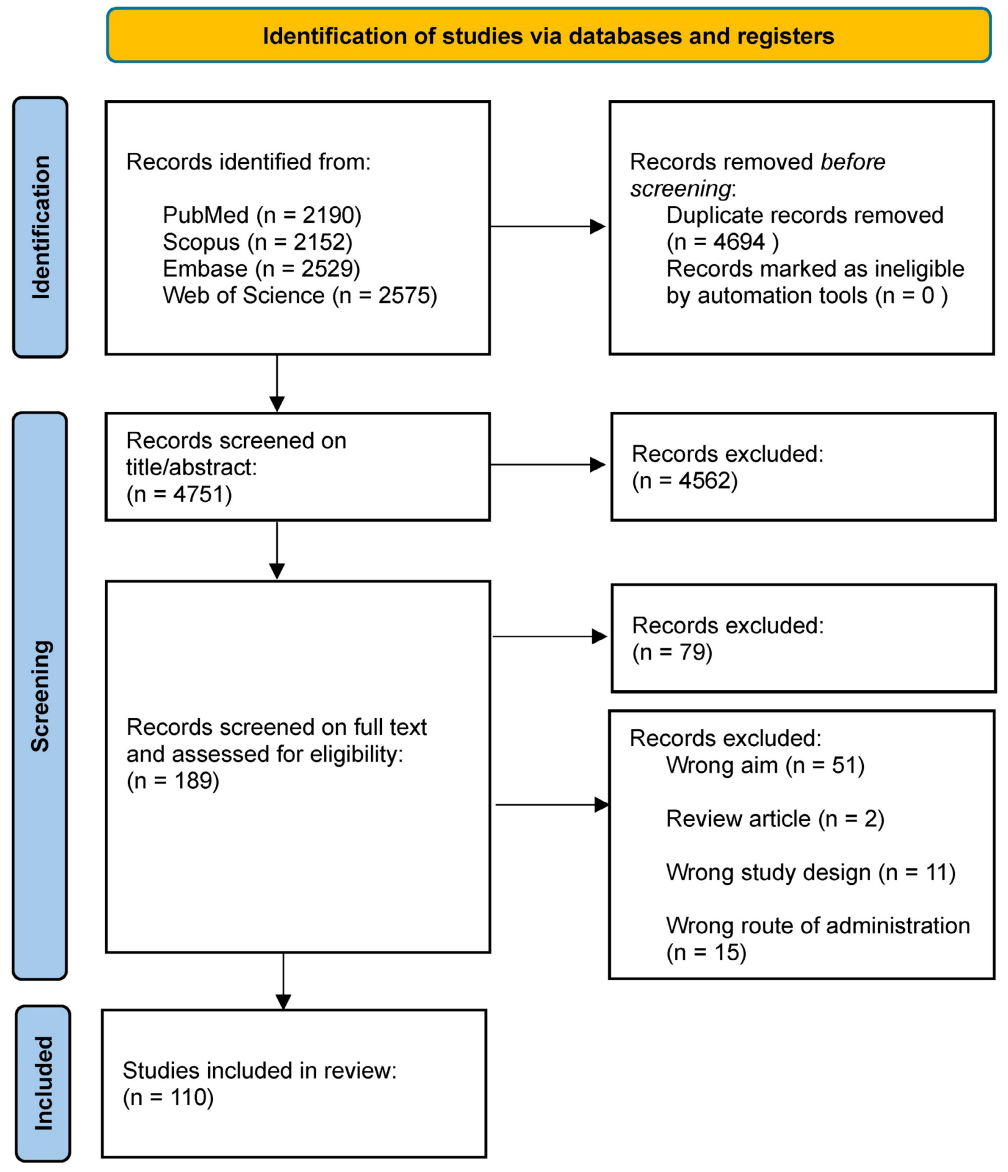
Diagram of literature search based on PRISMA-S guidelines.

Our literature search adhered to the PRISMA-S guidelines 200. We conducted a comprehensive search of PubMed, Scopus, Embase, and Web of Science, covering publications from 1989 to 2024 (see Figure 14). The initial phase involved the removal of duplicate studies from the database search results. Subsequently, we screened the remaining articles by examining titles and abstracts. The final stage entailed a thorough review of the full texts of these articles to determine their relevance and suitability for inclusion in our review.

### 3.2. Literature search strategy

The search strategy was designed to encompass a broad range of studies pertinent to our review. Utilising the resources of the University of Queensland library, we searched the PubMed, Scopus, Embase, and Web of Science databases using a combination of specific and alternative terms. Our search query was as follows:

("nose to brain" OR nose-to-brain OR intranasal OR intranasally OR "intranasal drug delivery" OR intramucosal) AND (Imaging OR Image OR "Magnetic resonance Imaging" OR MRI OR "Positron emission tomography" OR PET OR "Single photon emission computerised tomography" OR SPECT OR "Computed tomography" OR CT OR "Optical Imaging" OR OI OR "Fluorescence imaging" OR FLI OR "Bioluminescence imaging" OR BLI OR PET OR PET-CT OR PET-MRI OR PET) NOT (sinuses OR sinusitis OR facial OR respiratory OR sedation OR lung).

The literature search included studies published in English employing one or more imaging techniques, such as MRI, PET, SPECT, CT, OI, FLI, BLI, or combinations like PET-CT or PET-MRI, where the imaging agent was administered intranasally. Studies were excluded if they were review articles, brief communications, or abstracts lacking detailed methodology. Additionally, studies published in languages other than English or those focusing on IN administration for purposes other than brain imaging were not considered.

### 3.3. Literature search results

The results of our literature search are summarised in the figure below and structured according to the PRISMA-S guidelines 200.

## 4. Conclusions

The exploration of IN administration as a route for delivering imaging agents to the brain has been significantly advanced by diverse imaging modalities, each offering unique insights into the dynamics of agent delivery, distribution, and nasal cavity-to-brain interactions. The choice of imaging modality, contrast agent, and formulation like NEs, MEs, and NPs play a pivotal role in determining the efficiency, specificity, and safety of the IN administration process. PET Imaging is a cornerstone in understanding IN administration's molecular mechanisms. Utilising radioisotopes such as ^18^F and ^11^C, PET imaging is particularly valuable, as it offers a detailed view of the metabolic processes and direct transport of imaging agents within brain region parenchyma.

In examining the formulations used in these studies, the role of NE, ME, and NPs is particularly noteworthy. These engineered formulations enhance the bioavailability and brain targeting of imaging agents. NEs, for instance, demonstrate superior permeation rates and have been effectively employed in tracking agents like Gd and various drugs. MEs, with their unique composition, aid in stabilising and delivering imaging agents, as seen in studies involving citicoline-loaded niosomes. NPs, particularly AuNPs, have revolutionised CT imaging, offering enhanced visibility and precise tracking of drug distribution.

Each imaging modality and formulation offers distinct advantages and challenges. PET and SPECT are highly sensitive and provide rapid detection, making them suitable for real-time monitoring. OI, with its cellular-level resolution, is invaluable in toxicity studies and detailed tissue distribution analysis. MRI, with its superior resolution and non-invasive nature, is ideal for detailed anatomical and functional studies, while CT's value lies in its anatomical clarity, especially when combined with PET or SPECT.

The exploration of IN administration for imaging agents is a multidimensional field that benefits significantly from the diverse capabilities of these imaging modalities. The synergy between these techniques and the careful selection of appropriate formulations like NE, ME, and NPs are crucial in advancing our understanding of IN administration's potential in brain imaging and drug delivery. As this field evolves, further studies are needed to optimise the various imaging modalities and formulations, with the positive outcome of enhancing their efficiency, specificity, and safety in clinical applications.

The exploration of IN administration for delivering imaging agents to the brain is a growing field that has significantly advanced our understanding of drug delivery mechanisms, particularly where bypassing of the BBB is important. This review considered the SPECT, PET, OI, MRI, FUS and CT imaging modalities. The integration of these imaging techniques has offered unprecedented insights into the pharmacokinetics, biodistribution, and efficacy of IN administration. Advancements to date are pivotal not only in tracking and validating the efficacy of drug delivery but also in understanding the interaction of the agents used in accessing the brain tissues. Moreover, the review underscores the potential of IN administration in offering a non-invasive, efficient, and potentially safer alternative for drug delivery to the brain.

The ability of IN administration to bypass the BBB opens new avenues for treating neurological disorders, enhancing the efficacy of therapeutics, and reducing systemic side effects. It also paves the way for innovative diagnostic and therapeutic strategies, particularly in the context of neurodegenerative diseases, brain injuries, and brain tumours.

The field of IN administration in neuroimaging and drug delivery is on the brink of transformative advancements. Central to these developments is the need for new tracers and contrast agents optimised for the nose-to-brain pathway, focusing on enhanced targeting capabilities to improve efficacy. The integration of multiple imaging modalities promises a more comprehensive analysis, combining the strengths of each technique for a holistic understanding of drug dynamics in the brain.

Advancements in NP technology is poised to play a pivotal role, with multifunctional NPs offering exciting possibilities in improving the delivery and visualisation of imaging agents. These NPs, trackable by various imaging modalities and capable of carrying therapeutic payloads, are ripe for exploration. Safety and efficacy remain paramount, with long-term studies crucial for understanding the systemic effects and clearance mechanisms of IN administration agents. Innovations in delivery systems that can precisely regulate dosage, penetration, and release of imaging agents will further enhance the precision of IN administration.

**Figure 15:**
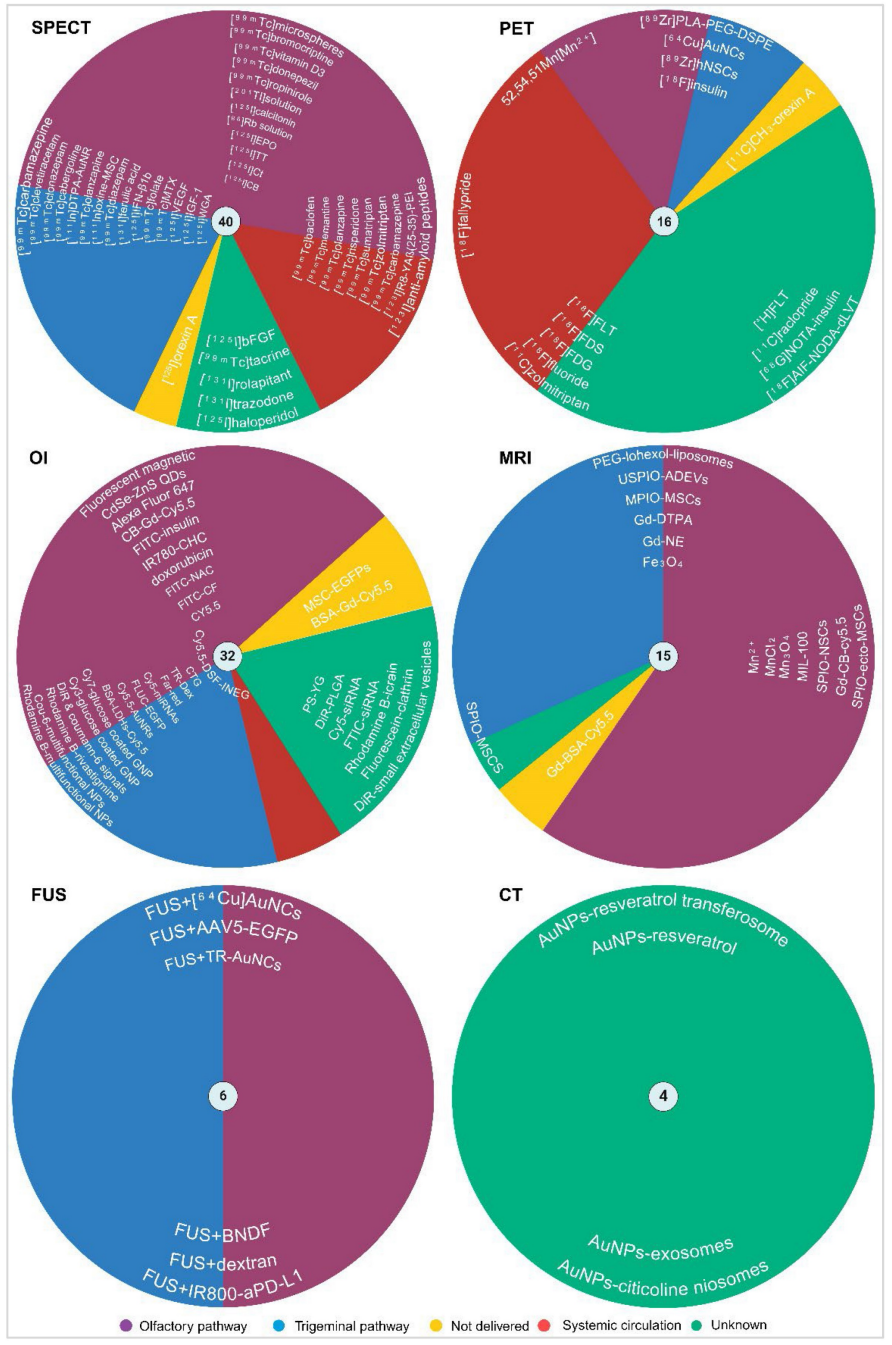
Comparative analysis of the imaging modalities in INDD to the Brain. This figure presents six pie charts, each representing a different imaging modality utilised to track the migration of specific tracers from the nasal cavity to the brain. Each chart details the tracer used, highlighting its unique pathway through the olfactory and trigeminal nerve routes. These visualisations underscore the diverse mechanisms and efficiency of nose-to-brain delivery pathways across different imaging agents. [created with BioRender.com].

In conclusion, the integration of advanced imaging modalities and innovative formulations in IN administration research has opened new frontiers in neurophysics and pharmacology. Figure 15 visually summarises the contributions of each imaging modality, elucidating the pathways and tracers used in the exploration of INDD.

This representation not only complements the detailed remarks within this review but also highlights the practical application and comparative benefits of these technologies in enhancing our understanding of drug dynamics in the brain. Table 2 provides a consolidated overview of the extensive work conducted in this field, underscoring the multi-dimensional nature of this research and its potential to revolutionise CNS diagnostics and therapeutics. This approach offers hope for more effective delivery of treatments for psychiatric and neurological disorders, with non-invasive techniques that may better target and mitigate these complex conditions. Future research is essential to address the highlighted challenges and to fully realise the potential of nose-to-brain delivery of imaging agents. Continued advancements in this area are crucial for improving the precision, efficiency, and safety of these delivery systems, ultimately enhancing patient outcomes in clinical settings. While the current review provides a comprehensive overview of the strengths and weaknesses of various imaging modalities and their applications in INDD, future research should also consider the development and optimisation of delivery devices and methods. These advancements are essential for maximising the efficacy of nose-to-brain delivery systems. Innovative delivery devices, such as micro-needles, nebulisers, and advanced nasal sprays, can significantly enhance the precision and efficiency of intranasal administration. Furthermore, the integration of real-time monitoring systems within these devices could provide immediate feedback on the delivery process, ensuring optimal dosing and minimising potential side effects. By focusing on both the imaging techniques and the delivery mechanisms, future studies can provide a more holistic approach to INDD, ultimately improving therapeutic outcomes and patient safety.

## Authorship contributions

AA, HP, BP and VV contributed to the conception and design of the work, the acquisition, analysis, or interpretation of data, and are accountable for all aspects of the work, ensuring that questions related to the accuracy or integrity of the work are appropriately investigated and resolved. KT helped draft and review the manuscript for its criticality. All authors provided approval for publication of the final version.

## Figures and Tables

**Table 1:** Comparison of strengths and weaknesses of imaging modalities and neuroimaging agents in the INDD.

SPECT			
Imaging Modality and Agent	Pros	Cons	Reference
SPECT	Due to its high sensitivity to gamma rays, SPECT can detect very low levels of radiopharmaceuticals, making it suitable for studying low-dose treatments delivered intranasally. Provides functional imaging and quantitative data on drug distribution and kinetics of drugs delivered via the nasal route. This helps in understanding the pharmacodynamics and pharmacokinetics of the drug. Can be combined with CT or MRI for comprehensive insights. This multimodal approach enhances the ability to correlate functional data with structural information, improving the understanding of drug distribution.	Limited spatial resolution compared to other modalities. SPECT provides lower spatial resolution, which can be a limitation when trying to visualise small structures or fine details within the brain. Longer scan times, which can be uncomfortable for patients. Exposure to ionising radiation.	182
^99m^Tc	High sensitivity to gamma rays, enabling detection of low-dose treatments Widely used and well-studied in nose-to-brain delivery. Short half-life (~6 h) reduces long-term radiation exposure. Suitable for functional imaging of drug distribution from nose to brain. Requires chelation with ligands, ensuring stability and effective delivery	Short half-life requires timely use, potentially limiting longer-term studies. Exposure to ionising radiation.	21
^86^Rb	High sensitivity, suitable for studying perfusion and drug distribution. Provides clear imaging of dynamic processes in nose-to-brain delivery. It does not require chelation, which simplifies preparation.	Long half-life (~18.6 days) limits the duration of imaging studies. High radiation dose, requiring careful handling and patient management.	23
^201^TI	Longer half-life (~73 h) allows for extended imaging sessions, which is useful in tracking longer-term INDD. Provides functional information on drug distribution. Does not require chelation, used in ionic form	Higher radiation doses can be a concern for patient safety. Lower spatial resolution compared to other tracers.	22
^125^I	High sensitivity, beneficial for detecting low concentrations of drugs delivered intranasally. Suitable for long-term studies, enabling monitoring over extended periods. Lower energy gamma emissions reduce radiation dose to patients. Often attached to proteins or peptides via chelation, enhancing targeting and stability.	lower spatial resolution can limit detailed imaging. Prolonged radiation exposure due to a longer half-life (~60 days) requires careful management.	21
^131^I	High sensitivity for detecting low-dose treatments in nose-to-brain delivery. Useful for both diagnostic and therapeutic purposes, providing versatile applications. Longer half-life (~8 days) allows for monitoring over extended periods. Typically chelated or attached to organic compounds to ensure stability.	High radiation dose can pose risks, particularly to the thyroid, requiring thyroid blockade. Can lead to significant thyroid uptake if not managed properly.	21
^123^I	High sensitivity and excellent imaging of brain perfusion, ideal for nose-to-brain studies. Lower radiation dose compared to 123I, making it safer for patients. Short half-life (13.2 h) reduces long-term radiation exposure. Often chelated or attached to proteins or peptides to enhance targeting and stability.	A shorter half-life requires rapid imaging and precise timing. Higher cost compared to other tracers, which can limit its use.	20, 21
^111^In	High sensitivity for detecting low-dose treatments in nose-to-brain delivery. Suitable for imaging infection, inflammation, and certain cancers. Half-life (2.8 days) allows for extended imaging sessions. Often chelated with DTPA, enhancing stability and targeting.	Intermediate radiation dose requires careful management. Can be costly due to preparation and handling requirements.	19, 21
PET			
PET	Higher sensitivity and resolution from SPECT, allowing for more precise imaging of drug distribution in the brain. This is crucial for accurately tracking the nose-to-brain delivery pathway. Quantitative capabilities allow for precise measurement of drug distribution. Can be combined with CT or MRI to provide both functional and anatomical information, enhancing the understanding of how intranasally delivered drugs interact with brain structures.	Expensive due to the cost of tracers and equipment. Short half-life of some tracers (e.g., [^11^C], [^18^F]) requires on-site cyclotron or rapid delivery. Exposure to ionising radiation. Similar to SPECT, PET involves exposure to ionising radiation, which must be carefully managed, especially in repeated or long-term studies.	182, 183
^18^F	High resolution and sensitivity due to the positron range of 0.6 mm, providing excellent spatial resolution. Suitable for functional imaging of drug distribution from nose to brain. Short half-life (109.8 min) reduces long-term radiation exposure. Widely used and well-studied in nose-to-brain delivery.	Requires on-site cyclotron for production. Short half-life limits time for imaging sessions. Exposure to ionising radiation.	75, 184
^11^C	High sensitivity and resolution with a positron range of 0.7 mm, resulting in very high spatial resolution. Suitable for studying metabolic processes and receptor binding. Short half-life (20.4 min) allows for quick imaging cycles.	Very short half-life requires rapid production and use. Requires on-site cyclotron. Exposure to ionising radiation.	184
^89^Zr	Long half-life (78.4 hours) allows for extended imaging sessions. Suitable for tracking long-term biological processes. High sensitivity for detecting low concentrations. Positron range of 1.3 mm, which may slightly reduce spatial resolution compared to shorter-range isotopes. Radiometal, requiring chelation for stability.	Longer half-life results in higher radiation dose. Requires chelation for stability. Exposure to ionising radiation.	184
^64^Cu	Intermediate half-life (~12.7 h) allows for flexibility in imaging time. Suitable for receptor imaging and tracking biological processes. Can be used in both PET and therapy. Positron range of 0.7 mm, providing excellent spatial resolution. Radiometal, requiring chelation for stability.	Requires chelation for stability. Intermediate radiation dose. Exposure to ionising radiation.	77, 184
^68^Ga	High sensitivity and resolution with a positron range of 3.2 mm, which can significantly reduce spatial resolution. Suitable for rapid imaging due to short half-life (68 min). Can be produced from a generator, making it accessible without a cyclotron. Radiometal, requiring chelation for stability.	Short half-life limits time for imaging. Requires chelation for stability. Exposure to ionising radiation.	78, 184
^51^Mn	Short half-life (46.2 min) suitable for short studies (rapid biological processes). Suitable for studying Mn uptake and biological processes. Provides insights into neurochemical pathways. Positron range of 4.3 mm, which can significantly reduce spatial resolution.	Limited availability and higher cost. Exposure to ionising radiation. Requires specific handling.	80, 110
^52^Mn	Longer half-life (~5.6 days) allows for extended imaging sessions. Suitable for tracking long-term biological processes. High sensitivity. Positron range of 3.5 mm, which can significantly reduce spatial resolution.	Higher radiation dose due to longer half-life. Requires specific chelation for stability. Limited clinical use.	80, 110
^54^Mn	Suitable for detailed imaging of Mn-related processes. Provides insights into brain function and neurochemical pathways.	Long half-life (~312.2 days) results in high radiation dose. Limited availability and high cost. Requires specific handling.	80
OI			
OI	High resolution allows for detailed visualisation at the cellular level, which is beneficial for studying the uptake and intracellular interactions of drugs delivered via the nasal route. Real-time imaging capabilities allow for dynamic studies of drug delivery and distribution, offering immediate feedback on the effectiveness of nose-to-brain delivery strategies. Useful for studying cellular uptake and interaction of drugs, providing insights into mechanisms of action and cellular responses.	Limited depth penetration, suitable mainly for superficial imaging. Issues with photobleaching and phototoxicity can affect results. Limited clinical applicability for deep brain imaging.	185, 186
QDs	High signal intensity, significantly greater than organic fluorophores, enabling detection of targeted biomarkers at lower expression levels. Broad excitation band and narrow, tunable fluorescence emission spectra, allowing for multiplexed imaging. Large surface area accommodating multiple probe molecules, enabling multivalent targeting to one or more biomarkers and increasing the target affinity of individual probes. Proven efficacy *in vivo* targeted imaging, as demonstrated by studies using functionalised QDs for targeting specific biomarkers in animal models.	Potential toxicity, particularly with heavy metal-containing QDs like CdSe-ZnS. Photobleaching issues, although less severe than organic fluorophores. Complex synthesis and functionalisation processes requiring careful handling. Limited clinical use due to regulatory and safety concerns.	187
Cy5.5	High sensitivity, allowing for the detection of low levels of biomarkers. Deep tissue penetration due to its near-infrared fluorescence, making it suitable for *in vivo* imaging. Suitable for long-term imaging studies because of its stable fluorescence signal. Can be conjugated to various targeting molecules, enhancing specificity for nose-to-brain delivery.	Photobleaching can occur, reducing signal over time. Limited clinical use due to regulatory and safety concerns. Requires careful handling and proper storage to maintain stability. Higher cost compared to other fluorophores, which can limit widespread use.	188
CTG	High sensitivity and specificity for mitochondrial targeting. Reactive oxygen species responsive properties allow for the detection of oxidative stress-related changes. Suitable for real-time imaging of cellular processes.	Limited penetration depth, primarily useful for superficial imaging. Potential for photobleaching and phototoxicity, which can affect long-term imaging. Limited clinical applicability for deep tissue imaging in the brain.	120
EGFP	EGFP provides a bright and distinct green fluorescence, which makes it easy to detect and visualise in biological tissues. EGFP is generally considered non-toxic to cells, making it suitable for long-term studies without harming the cells being tracked. EGFP can be stably expressed in cells, allowing for continuous monitoring over extended periods. Compatible with various imaging modalities, such as fluorescence microscopy and flow cytometry, providing flexibility in experimental design.	EGFP fluorescence can be difficult to distinguish from tissue autofluorescence, especially in the brain, which can lead to false-positive results. The fluorescence signal from EGFP may not penetrate deep tissues effectively, limiting its use for imaging deep brain structures. In some cases, EGFP can elicit an immune response, which may affect the viability and behaviour of the labelled cells. Proper controls are required to differentiate between a true EGFP signal and autofluorescence, adding complexity to experimental protocols.	122
FITC	High fluorescence and quantum yield, making it suitable for sensitive detection. Widely used and well-studied in bioanalytics, laboratory diagnostics, and biomedical diagnostics. Can be conjugated to various molecules, such as proteins and peptides, enhancing its versatility for targeted imaging. FITC has good sensitivity for detecting lower concentrations of labelled compounds in tissues. It is a well-established dye in bioimaging and diagnostic applications, providing a reliable option for researchers.	Photobleaching can occur, reducing signal over time. Sensitive to pH changes, which can affect its fluorescence properties. Limited tissue penetration compared to near-infrared dyes. Potential toxicity if not properly managed, particularly in long-term studies. FITC can sometimes contribute to autofluorescence, which may interfere with the specific signal and complicate the interpretation of imaging results.	123, 188
Alexa Fluor 647	It provides high sensitivity. The fluorescent label enables clear and detailed visualisation of the distribution and uptake. It offers high specificity, reducing background noise and enhancing the contrast-to-noise ratio. Fluorescent imaging allows for non-invasive tracking of delivery in live tissues, providing real-time data on the delivery process without the need for more invasive procedures. The fluorescent label is compatible with various imaging modalities.	Restricts visualisation of deeper brain tissues. Prolonged light exposure can fade fluorescence, affecting imaging accuracy and duration. Some background signals may interfere with results, requiring proper controls and calibration. Primarily shows extracellular distribution, providing less detailed information on intracellular uptake and localisation than electron microscopy.	124
TR	It provides strong fluorescence, making it easy to detect and visualise in biological tissues. It is photostable, allowing for prolonged imaging sessions without significant loss of signal. Compatible with various imaging modalities such as fluorescence microscopy and flow cytometry, providing flexibility in experimental design. It reduces background autofluorescence, which enhances the clarity and specificity of imaging results.	The fluorescence signal from TR may not penetrate deep tissues effectively, limiting its use for imaging deep brain structures. Despite its photostability, prolonged exposure to light can still cause photobleaching, which may affect the quality of long-term imaging. Requires proper controls to differentiate between true TR signal and autofluorescence, adding complexity to experimental protocols. Limited clinical applicability for deep tissue imaging in the brain, requiring further research and validation.	125
Far-red	Allows for better visualisation of structures located beneath the surface. Less autofluorescence, results in higher signal-to-noise ratios and clearer images. Low risk of phototoxic effects during imaging. Can be used in various imaging modalities, including fluorescence microscopy, flow cytometry, and photoacoustic imaging.	High-quality far-red imaging agents can be expensive. Can lead to background noise and reduce the specificity of imaging. Susceptible to photobleaching under intense or prolonged illumination. Can limit the duration of imaging sessions and the ability to capture long-term dynamic processes.	132, 189
DiR	High quantum yield and good stability. Near-infrared fluorescence allows for deeper tissue penetration compared to visible spectrum dyes. Low autofluorescence background in biological tissues.	Limited availability of detailed information on its use in nose-to-brain delivery. Potential for aggregation in biological systems, which may affect accuracy.	127
IR800	IR800 is a near-infrared fluorescent dye with high sensitivity and specificity. It allows for the clear and precise imaging of labelled molecules within biological tissues. IR800 has a low autofluorescence background, which enhances the contrast and clarity of the images, making it easier to distinguish the labelled molecules from the surrounding tissue. IR800 provides stable labelling of proteins and other molecules, ensuring that the fluorescence signal remains strong and reliable over time, which is crucial for long-term studies. The use of IR800 allows for non-invasive tracking of the labelled molecules, enabling real-time monitoring of their distribution and accumulation in live subjects. The IR800 is compatible with various imaging modalities, including fluorescence imaging and near-infrared spectroscopy, providing flexibility in experimental design and data collection.	Potential for phototoxicity, especially with prolonged exposure to intense light sources during imaging. The process of conjugating IR800 to proteins or other molecules can be complex and may require optimisation to ensure efficient and specific labelling without affecting the biological activity of the target molecule. While near-infrared dyes have better tissue penetration compared to visible light dyes, the penetration depth is still limited, which can be a disadvantage when imaging deeper tissues or larger animals. The conjugation of IR800 to molecules may alter their biodistribution, potentially affecting the physiological relevance of the imaging results.	176
PS-YG	The fluorescent properties of PS-YG enable clear and detailed visualisation of nanoplastics within biological tissues, facilitating the study of their distribution, accumulation, and clearance. Fluorescence signals from PS-YG allow for precise quantitative analysis of nanoplastic presence in various regions, aiding in the assessment of neurotoxic effects and the effectiveness of potential mitigation strategies. The ability to monitor PS-YG in real-time offers valuable insights into the dynamic processes of nanoplastic movement, interaction with cells, and exocytosis within the brain.	The use of fluorescent dyes can introduce phototoxicity, potentially affecting cell viability and confounding the results related to neurotoxicity. The introduction of fluorescent PS-YG could potentially interfere with normal cellular processes, adding an additional layer of complexity to the interpretation of neurotoxicity and exocytosis data.	133
COU-6	High fluorescence intensity, enabling sensitive detection and imaging of cellular components and pathways. Excellent photostability, allowing for prolonged imaging sessions without significant loss of signal. Effective in tracking intracellular processes and distribution of NPs in vitro and *in vivo*. Provides valuable data on drug delivery efficacy and mechanisms in preclinical studies.	Fluorescence can be quenched under certain conditions, which may complicate data interpretation. May require careful calibration and controls to account for potential quenching effects in different environments. Potential issues with cytotoxicity at higher concentrations or prolonged exposure.	136
Cy5	High sensitivity, useful for detecting low levels of biomarkers. Deep tissue penetration due to near-infrared fluorescence. Suitable for multiplexed imaging due to its distinct emission spectra. Can be conjugated to various targeting molecules, enhancing specificity for nose-to-brain delivery.	Photobleaching can occur, reducing signal over time. Limited clinical use due to regulatory and safety concerns. Requires careful handling and proper storage to maintain stability.	188
Cy3	High fluorescence and quantum yield, suitable for fluorescence resonance energy transfer studies. Useful for detecting low levels of biomarkers. Can be conjugated to various targeting molecules, enhancing specificity for nose-to-brain delivery.	Photobleaching can occur, reducing signal over time. Limited tissue penetration compared to near-infrared dyes. Limited clinical use due to regulatory and safety concerns.	188
Cy7	Long-wavelength emission allows for deep tissue penetration, making it suitable for *in vivo* imaging. Reduced background fluorescence due to near-infrared range. Can be conjugated to various targeting molecules, enhancing specificity for nose-to-brain delivery.	Photobleaching can occur, reducing signal over time. High cost compared to other fluorophores, which can limit widespread use. Limited clinical use due to regulatory and safety concerns.	188
FLUC-EGFP	High sensitivity and specificity, ideal for monitoring biological processes *in vivo*. Combines dual imaging capabilities. Real-time imaging, enables monitoring of dynamic processes. Provides accurate and reliable data. Suitable for tracking tumour growth, gene expression, and cellular interactions.	Fluorescence imaging can suffer from interference. Restricted use in deep tissue imaging. Signal loss over time affects long-term studies. May trigger immune responses in some subjects. Requires sophisticated and costly equipment.	143
Rhodamine B	High fluorescence and quantum yield, making it a sensitive marker for imaging. Commonly used in bioimaging due to its strong fluorescence properties. Effective in various experimental setups for tracking and imaging cellular components. It can be activated under visible light for the degradation of other dyes and antibiotics, illustrating its versatility in research. Utilised fluorescent labelling to study biochemical pathways and cellular structures.	Toxic to the human body, especially when ingested, causing oxidative stress and potential liver dysfunction or cancer. Can cause acute poisoning if exposed to large amounts in a short period. Requires careful handling and proper storage to mitigate toxic effects. Regulatory and safety concerns limit its widespread clinical use.	190
Doxorubicin	The doxorubicin molecule's inherent fluorescence allows for combined therapeutic and imaging capabilities, making it an excellent theranostic agent. Fluorescence imaging of organs or cells after doxorubicin injection provides valuable information on drug biodistribution.	Self-quenching of doxorubicin fluorescence at high concentrations, leading to potential misinterpretation of data. Potential toxicity.	191
MRI			
MRI	Excellent spatial resolution and soft tissue contrast, making it ideal for detailed anatomical imaging of the brain. This helps visualise the precise location and distribution of intranasally delivered drugs. Non-invasive, with no exposure to ionising radiation, making it a safer option for repeated imaging studies. This is particularly advantageous for long-term studies on nose-to-brain delivery.	Expensive and requires extensive infrastructure. Longer scan times can be uncomfortable for patients. Lower sensitivity compared to nuclear imaging techniques like PET and SPECT for detecting low concentrations of tracers, which can be a limitation in some studies.	192
Gd	Gd-based contrast agents have high relaxivity, which enhances the contrast of MRI images by shortening the relaxation times of nearby water protons. Gd contrast agents provide strong and effective contrast enhancement, making it easier to distinguish between different tissues and identify abnormalities. Gd contrast agents are widely used in clinical practice and have been extensively studied, providing a robust understanding of their benefits and risks. Gd contrast agents can be used in various types of MRI scans, including brain, spine, liver, and vascular imaging. They have better solubility and biodegradability than iron oxide NP-based agents. Gd are more suitable for T1-weighted MRI. T1-weighted MRI is particularly effective for brain imaging. Changes in T1-weighted MRI signals are more easily detectable in the brain.	Gd contrast agents are associated with a rare but serious condition called nephrogenic systemic fibrosis in patients with severe kidney dysfunction, which limits their use in this patient population. Gd can be retained in the brain and other tissues, raising concerns about long-term safety, especially with repeated use. Some patients may experience allergic reactions to Gd contrast agents, ranging from mild to severe anaphylactic reactions. Gd contrast agents can be expensive, adding to the overall cost of MRI procedures. Requires careful administration and monitoring, particularly in patients with renal impairment or other risk factors. Free Gd ions are toxic, so Gd contrast agents must be carefully formulated to keep Gd bound within the chelate structure.	118, 193
Mn	Mn-based contrast agents have high sensitivity, enhancing the contrast of MRI images. Mn provides effective T1-weighted imaging, which is particularly useful for brain imaging and detecting small lesions. Mn can act as a calcium analog, entering active neurons and providing insights into neuronal activity and brain function. Useful for functional imaging studies, as Mn can highlight areas of active metabolism and ion exchange. Mn-based agents are generally considered to have minimal toxicity at low doses, making them safer for certain applications than other contrast agents.	At high concentrations, Mn can be neurotoxic, potentially causing adverse effects on the nervous system. Mn-based agents may have limited solubility, which can affect their efficacy and administration. The imaging window for Mn-based agents can be relatively short, requiring precise timing for optimal imaging results. Requires careful dosage control to avoid toxicity while ensuring effective imaging. Mn-based agents are not as widely used or studied as Gd-based agents, limiting their availability and familiarity among clinicians.	149, 151
USPIO	Less than 50 nm in size. High sensitivity, enhancing contrast and enabling detection of small lesions. Long circulation time, allowing extended imaging periods. Useful for macrophage imaging and inflammation studies. Biocompatible with minimal toxicity. Suitable for both T1 and T2-weighted imaging. Minimal alteration of cell morphology.	Potential for iron overload with repeated use. Complex interpretation of T2 signal reductions. Limited availability in some clinical settings. Higher cost compared to conventional agents. Requires special handling and storage.	154, 194, 195
SPIO	Less than ~250 nm in size. Effective T2 contrast agent. Useful for imaging the liver and spleen and tracking stem cells. Well-studied and widely used in clinical settings. Biocompatible with low toxicity. Strong reduction in T2 signal intensity.	Shorter circulation time compared to USPIO. Limited effectiveness for T1-weighted imaging. Potential for iron overload with repeated use. Complex interpretation of T2 signal reductions. Requires special handling and storage.	194-196
MPIO	The particle size is greater than 0.9 µm. High sensitivity for detecting small vascular structures. Useful for cell labelling and tracking. Effective T2 contrast agent. Strong reduction in T2 signal intensity. Biocompatible with minimal toxicity.	Potential for iron overload with repeated use. Larger size may limit use in some applications. Shorter circulation time compared to USPIO. Requires special handling and storage. Limited availability in some clinical settings.	195
Iohexol	High solubility in water Generally well-tolerated Versatile in imaging modalities (CT and CEST MRI) Strong CEST contrast from amide protons Non-invasive tracking of drug delivery	Expensive Short duration of contrast effect Potential allergic reactions Risk of nephrotoxicity Requires careful handling and storage	165
MIL-100 (Fe)	High surface area and porosity, allowing for drug loading and release. Useful for both imaging and therapeutic applications. Biocompatible and can be functionalised for targeted delivery. Provides strong MRI contrast and can be used for T1 and T2 imaging. Versatile applications in drug delivery and imaging.	Potential for toxicity due to metal ion release. Limited clinical data on safety and efficacy. Potential for instability under physiological conditions.	134
FUS			
FUS	A noninvasive method to temporarily disrupt the BBB, enhancing the delivery of therapeutic agents to the brain. This is particularly useful for facilitating the uptake of intranasally delivered drugs. Can be used to target specific brain regions, improving the therapeutic efficacy and reducing systemic side effects. No ionising radiation, making it a safer option for repeated use.	Requires precise targeting and advanced technical expertise, Inaccurate targeting may lead to unintended damage to brain tissues. Limited availability in clinical settings. Potential thermal and mechanical effects on tissues if not properly controlled.	197
Microbubble	Microbubbles can improve the delivery of therapeutic agents across the BBB when combined with FUS. This is due to the cavitation effect, which increases the permeability of the BBB. When used with FUS, microbubbles can target specific brain regions, enhancing the localisation of drug delivery and minimising systemic exposure. The use of microbubbles in combination with the US offers a non-invasive method for INDD, reducing the need for surgical interventions. Microbubbles can be used to deliver a wide range of agents, including proteins, NPs, and small molecules, making them a versatile tool in medical treatments.	The cavitation effect, while useful for increasing permeability, can also cause damage to surrounding tissues if not carefully controlled. Microbubbles have a relatively short half-life in the circulatory system, which can limit the duration of their therapeutic effects and necessitate repeated administrations. Combining microbubbles with FUS requires specialised equipment and expertise, which may not be readily available in all clinical settings. While effective for targeting specific brain regions, the penetration depth of microbubbles is limited, which might restrict their use in treating deeper or more diffuse brain pathologies​.	168, 173, 176, 177
CT			
CT	High spatial resolution, particularly useful for structural imaging of the brain and visualising the anatomical pathways of intranasally delivered drugs. The rapid imaging capabilities of CT are beneficial for patient comfort and for obtaining quick results, making it suitable for dynamic studies. CT can be combined with PET and SPECT to provide comprehensive functional and anatomical imaging, enhancing the overall understanding of drug distribution and effects.	Exposure to ionising radiation, which can be a concern for repeated imaging. Compared to MRI, CT has lower contrast resolution for soft tissues, which can limit its ability to provide detailed anatomical information. While excellent for structural imaging, CT alone does not provide functional information, which can limit its utility for certain studies without additional modalities.	198
AuNPs	AuNPs have a high X-ray attenuation coefficient, making them highly effective as contrast agents for CT imaging. This allows for clear and detailed imaging of internal structures​​. AuNPs are biocompatible, reducing the risk of adverse reactions when used as contrast agents. This makes them safer for repeated use in clinical settings​​. The surface of AuNPs can be easily modified with various functional groups, allowing for targeted imaging and the attachment of therapeutic agents. This versatility enhances their application in targeted imaging and therapy​​. AuNPs can be used in combination with other imaging modalities, such as MRI, PET, and fluorescence imaging, to provide comprehensive diagnostic information from multiple techniques​​.	Potential Toxicity​​. The preparation and functionalisation of AuNPs can be complex and require precise control over particle size, shape, and surface chemistry, which may increase the cost and complexity of their production​​. Despite their effectiveness in CT imaging, AuNPs may have limited penetration depth in certain tissues, potentially restricting their use in imaging deeper anatomical structures​​ AuNPs can aggregate under certain conditions, which might reduce their effectiveness as contrast agents and could pose challenges in ensuring consistent performance​​. The use of AuNPs as clinical contrast agents may face significant regulatory hurdles, requiring extensive safety and efficacy data before they can be approved for widespread clinical use​.	199

**Table 2:** Summary of key research findings on INDD systems for enhanced brain targeting, monitored by medical imaging: a systematic review of imaging agents, modalities, objectives, and achievements.

Section	Imaging agents	Modalities	Brain retention time/ scan window time	Validation method	Objectives	Achievements	Study
2.1.1	[^99m^Tc]	SPECT/Gamma	8 h	Biodistribution study.	To prepare and evaluate MEs containing zolmitriptan for rapid drug delivery to the brain. It also aimed to characterise these ME and assess their biodistribution in rats through different administration routes.	The result demonstrated substantial uptake of the drug into the brain following IN administration of the zolmitriptan mucoadhesive ME. This imaging evidence supports the effectiveness of the IN administration of zolmitriptan mucoadhesive ME for targeted drug delivery to the brain.	Vyas *et al.* (2005) 24
2.1.1	[^99m^Tc]	SPECT/Gamma	8 h	Biodistribution study & transmission electron microscopy.	To develop and assess clonazepam MEs for INDD, specifically targeting the treatment of acute status epileptic patients.	The study indicated that the IN administration of clonazepam mucoadhesive ME resulted in superior brain uptake of the drug, as evidenced by higher brain/blood uptake ratios and clear visualisation in rabbit brain scintigraphy imaging.	Vyas *et al.* (2006) 26
2.1.1	[^99m^Tc]	SPECT/Gamma	8 h	Biodistribution study & electron microscopy.	To formulate ME of sumatriptan and sumatriptan succinate and compare their effectiveness in delivering the drug rapidly to the brain during acute migraine attacks.	The imaging results confirmed that sumatriptan succinate was uptaken in the brain more efficiently when administered through IN administration of both formulated MEs than in other forms and delivery methods.	Vyas *et al.* (2006) 27
2.1.1	[^99m^Tc]	SPECT/Gamma	8 h	Biodistribution study.	To formulate an NE containing risperidone for targeted INDD drug delivery. Aimed to compare the biodistribution and brain localisation of NE-containing risperidone in different formulations for IV injection and IN administration in Swiss albino rats.	The result demonstrated rapid and extensive transport of the drug to the brain, and bypassing the BBB. This was evident from higher brain/blood uptake ratios and drug transport efficiency when compared to the other formulations and administration routes.	Kumar *et al.* (2008) 28
2.1.1	[^99m^Tc]	SPECT/Gamma	4 h	Biodistribution study.	To prepare and characterise cabergoline IN administration ME formulations for effective brain drug delivery and assess their performance in weight control.	Imaging confirmed the localisation of the cabergoline in the brain following IN, IV, and oral administrations. The [^99m^Tc]cabergoline formulations showed higher brain/blood uptake ratios, drug targeting efficiency, and direct drug transport to the brain via IN administration compared to IV injection or oral administration.	Sharma *et al.* (2009) 29
2.1.1	[^99m^Tc]	SPECT/Gamma	8 h	Biodistribution & pharmacokinetic studies.	To evaluate the drug biodistribution of thiolated chitosan NPs of tizanidine hydrochloride after IN administration.	Results showed that the brain uptake of the drug was significantly enhanced after the thiolation of chitosan, indicating improved transnasal permeation.	Patel *et al.* (2012) 30
2.1.1	[^99m^Tc]	SPECT/Gamma	8 h	Biodistribution study.	To evaluate the effectiveness of direct nose-to-brain delivery of tacrine, a cholinesterase inhibitor, in order to enhance its bioavailability, circumvent the first-pass hepatic metabolism, and reduce hepatotoxicity, aiming to improve its therapeutic profile for Alzheimer's disease treatment.	The study demonstrated that IN administration of tacrine when compared to IV injection, resulted in faster delivery to the brain, higher brain/blood concentration ratios, and significant direct transport of the drug to the brain.	Jogani *et al.* (2007) 32
2.1.1	[^99m^Tc]	SPECT/Gamma	6 h	No validation.	To develop mucoadhesive microspheres containing tramadol hydrochloride. Additionally, the study sought to evaluate the nose-to-brain delivery of these radiolabeled microspheres through a gamma scintigraphic study.	They confirmed a significant accumulation of [^99m^Tc]microspheres in the brain over a prolonged period following IN administration.	Belgamwar *et al.* (2011) 31
2.1.1	[^99m^Tc]	SPECT/Gamma	9 h	Transmission electron microscopy.	The study aimed to assess the brain-targeting effects and fate of ropinirole loaded in a true NE when IN-administered. It also aimed to evaluate the impact of homogenisation on drug uptake by olfactory nerves.	The study found differences in brain localisation and maximum concentration between the two radiolabelled nano-formulations tested. These differences suggested that the formulation was directly transported from nose-to-brain.	Mustafa *et al.* (2012) 33
2.1.1	[^99m^Tc]	SPECT/Gamma	8 h	Biodistribution & pharmacokinetic studies.	To explore the effectiveness of chitosan NPs as a delivery vehicle to improve the targeting of bromocriptine to the brain when administered IN.	The study successfully demonstrated that chitosan NPs enhance the delivery of bromocriptine to the brain via IN administration.	Md *et al.* (2013) 34
2.1.1	[^99m^Tc]	SPECT/Gamma	8 h	Biodistribution study.	To develop and evaluate carbamazepine-loaded MEs for IN administration to treat epilepsy, they wanted to determine the drug's uptake and distribution in the brain when delivered via this nose-to-brain.	The IN administration of carbamazepine loaded in a mucoadhesive ME form showed 2-3 times higher distribution in the brain compared to its IV injection counterpart. DTE% and DTP% were highest for carbamazepine-loaded MEs after IN administration, and rat brain imaging confirmed higher IN administration uptake of carbamazepine into the brain.	Patel *et al.* (2014) 35
2.1.1	[^99m^Tc]	SPECT/Gamma	8 h	Biodistribution study.	To enhance the delivery of diazepam to the brain by IN administration using PLGA NPs.	Gamma scintigraphy images demonstrated effective nose-to-brain delivery of diazepam when encapsulated in PLGA NPs. The images showed significant uptake of the drug in the brain, corroborating the biodistribution results and indicating that the optimised NP formulation successfully enhances the direct transport of diazepam from nose-to-brain.	Sharma *et al.* (2015) 36
2.1.1	[^99m^Tc]	SPECT/Gamma	8 h	Biodistribution & pharmacokinetic & targeting studies.	To use SPECT imaging for visual validation and confirmation of brain uptake of the developed zolmitriptan-loaded nanostructured polymeric carriers.	The SPECT/CT images provided evidence of significant accumulation and uptake of the nanocarriers in the brain.	Mandlik *et al.* (2018) 37
2.1.1	[^99m^Tc]	SPECT/Gamma	1 h	Biodistribution study.	To develop a stable radiolabeled levetiracetam for brain imaging, particularly targeting SV2A receptors, and compare its effectiveness across different administration routes.	The team successfully created a [^99m^Tc]levetiracetam ME with a high radiochemical yield. It demonstrated that its IN administration led to superior brain targeting compared to other routes, making it a promising candidate for SPECT imaging of SV2A receptors.	Rashed *et al.* (2018) 38
2.1.1	[^99m^Tc]	SPECT/Gamma	24 h	Biodistribution study.	To develop and optimise baclofen-loaded PLGA NPs using the nanoprecipitation method, and to evaluate their potential as a carrier for baclofen in treating neuropathic pain, focusing on the nose-to-brain delivery pathway.	*In vivo* studies in rats showed maximum uptake of the NPs from the nose-to-brain at 3 h post IN administration, with significant dispersion in the brain and blood, confirming the potential of the developed PLGA NPs as a carrier for baclofen in treating neuropathic pain via the nose-to-brain pathway.	Nigam *et al.* (2018) 39
2.1.1	[^99m^Tc]	SPECT/Gamma	8 h	Biodistribution study.	To develop and assess a [^99m^Tc]olanzapine complex as an atypical antipsychotic agent for brain imaging and drug delivery.	Imaging results indicated substantial brain uptake after both IN administration and IV injection, providing clear imaging windows at specific times post-administration.	Ibrahim *et al.* (2020) 40
2.1.1	[^99m^Tc]	SPECT/Gamma	24 h	Transmission electron microscopy & pharmacokinetic study & biodistribution study.	The study aimed to develop an NE loaded with memantine, a drug used against Alzheimer's disease, for IN administration in mice and to explore the efficacy of this delivery method compared to oral and IV.	The [^99m^Tc]memantine NE resulted in higher brain uptake at 1.5 h and maximum drug uptake in the brain compared to the other administration routes.	Kaur *et al.* (2020) 41
2.1.1	[^99m^Tc]	SPECT/Gamma	24 h	Biodistribution study.	To develop and evaluate an NE loaded with donepezil for IN administration.	The NE loaded with donepezil demonstrated effective IN administration for Alzheimer's treatment. Imaging studies confirmed successful drug delivery from the nose-to-brain in animal models.	Kaur *et al.* (2020) 42
2.1.1	[^99m^Tc]	SPECT/Gamma	24 h	Biodistribution & pharmacokinetic & targeting studies.	To develop and evaluate a vitamin D3-loaded NE for the effective treatment of cerebral ischemia.	They successfully developed a stable and effective vitamin D3-loaded NE. This formulation showed improved brain deposition and efficacy when IN-administered, as compared to IV injection. These results suggest a promising nasal delivery method for treating cerebral ischemia.	Kumar *et al.* (2020) 43
2.1.1	[^99m^Tc]	SPECT/Gamma	4 h	Biodistribution study.	To address the challenge of delivering therapeutic agents across the BBB for the treatment of glioblastoma. The approach involved formulating chemotherapeutic agents, specifically MTX, conjugated with a bifunctional chelating agent and radiolabeled with [^99m^Tc], within micelles for IN administration.	The study successfully created [^99m^Tc] MTX-loaded micelles. It enhanced the nose-to-brain uptake of these micelles compared to the solution form of the drug. Moreover, Increased anti-cancer activity (3-fold) in drug-conjugate loaded micelles versus the drug solution.	Upadhaya *et al.* (2023) 44
2.1.1	[^99m^Tc]	SPECT/Gamma	4 h	Biodistribution study.	To develop a non-invasive diagnostic tool for brain tumours, particularly targeting gliomas, by exploiting the overexpression of folate receptors in these tumours. The approach involved creating a radiolabeled folate encapsulated in micellar carriers for IN administration.	The study successfully developed micellar carriers encapsulating a radiolabeled folate conjugate. The *in vivo* studies demonstrated high brain uptake of the micelles (around 16% in 4 h) compared to the radiolabeled folate solution. Enhanced permeation and mucoadhesivity of the micelles, making them effective for brain targeting. Safety and biocompatibility of the micelles for IN administration. SPECT imaging in higher animals (rabbits) confirmed enhanced brain uptake of the micelles.	Upadhaya *et al.* (2023) 45
2.1.2	[^86^Rb] , [^201^TI]	SPECT/Gamma	24	Pharmacokinetic & autoradiography studies.	To investigate the axonal transport of radioactive ^86^Rb and ^201^TI through the olfactory nerve in mice, specifically examining how these substances are transported from nose-to-brain following IN administration.	They found a pronounced accumulation of [^86^Rb] and [^201^TI] in the olfactory bulb on the same side as the administration within 6 h of IN delivery. The study provided strong evidence of direct axonal transport via the olfactory nerve pathway, potentially reflecting similar transport behaviour of potassium in the olfactory system.	Kanayama *et al.* (2005) 52
2.1.2	[^201^TI], [^54^Mn]	SPECT/Gamma	3 h	Neuronal tracer & autoradiography studies.	To explore the transport mechanisms of [^201^Tl] and [^54^Mn] ions in the olfactory system of mice, focusing on their movement from the nasal cavity to the olfactory bulb following IN administration.	They concluded that both [^201^TI] and [^54^Mn] ions are transported to the olfactory bulb through the olfactory nerve fibres. This transport was significantly reduced in mice with transected olfactory nerve fibres, demonstrating the critical role of these nerve fibres in the transportation process.	Kinoshita *et al.* (2008) 53
2.1.2	[^201^TI]	SPECT/Gamma	24 h	No validation.	To create a visual representation of the olfactory pathway through which smells are transported to the brain in humans.	The study successfully demonstrated that 24 h after IN administration of [^201^TI], its highest concentration was observed in the olfactory bulb located at the front base of the skull, passing through the cribriform plate. This was accomplished without causing any impairment in the participants' sense of smell.	Shiga *et al.* (2011) 54
2.1.3	[^125^I]	SPECT/Gamma	6 days	Immunohistochemistry study.	To investigate whether Ct and CB with IN administration could target the olfactory nerves, epithelium and olfactory bulbs, in addition to nasal-associated lymphoreticular tissues.	They demonstrated that these compounds did indeed enter and persist in the olfactory regions for up to 6 days, but were not present in nasal-associated lymphoreticular tissues beyond 24 h.	Van Ginkel *et al.* (2000) 62
2.1.3	[^125^I]	SPECT/Gamma	3 h	Immunohistochemistry study.	To develop a noninvasive method for imaging Aβ deposition in Alzheimer's disease using IN administration of radiolabeled compounds.	They revealed that IN administration [^125^I] labelled bFGF accumulated significantly in the brains of transgenic mice modelling Alzheimer's disease, specifically labelling Aβ plaques in the cortex and microvessels.	Shi *et al.* (2002) 63
2.1.3	[^125^I]	SPECT/Gamma	24 h	Immunohistochemistry, immunoblotting and autoradiography studies.	To explore how IGF-1 is delivered to the CNS following IN administration in rats, focusing on the transport pathways from the nasal passages to the CNS.	It was found that IGF-1 rapidly reaches the CNS through two main routes: the peripheral olfactory system and the peripheral trigeminal system, bypassing the BBB. IN administration also affected the deep cervical lymph nodes but not the CSF.	Thorne *et al.* (2004) 64
2.1.3	[^125^I]	SPECT/Gamma	45 min	Autoradiography study.	To examine the targeting of the nervous system by IFN-β1b, particularly following its IN administration in monkeys,	The study found that IN administration IFN-β1b rapidly targeted the nervous system, with significant localisation in the olfactory bulbs and trigeminal nerve.	Thorne *et al.* (2008) 65
2.1.3	[^125^I]	SPECT/Gamma	30 min	Autoradiography study.	To assess the potential of delivering VEGF directly into the CNS following IN administration in rats.	The study demonstrated substantial delivery throughout the CNS. The highest concentrations were found in the trigeminal nerve, optic nerve, olfactory bulbs, and several brain regions, including the striatum, medulla, and cortex.	Yang *et al.* (2008) 66
2.1.3	[^125^I]	SPECT/Gamma	2 h	OI tracers validation & biodistribution study.	To investigate the pathways to transport WGA-NPs into the brain following IN administration.	The study found rapid absorption across the olfactory epithelium and subsequent transfer to the olfactory bulb. The NPs also reach the brain via the trigeminal nerve pathway, particularly influencing the distribution in the caudal brain areas. However, the CSF pathway appeared to contribute minimally to this process.	Liu *et al.* (2012) 67
2.1.3	[^125^I]	SPECT/Gamma	2 h	Biodistribution study.	To investigate the brain delivery efficiency of Haloperidol when labelled with [^125^I] and administered through different formulations: IV injection, IN administration, and IN administration ME.	The study found that the IN administration ME formulation showed superior brain uptake compared to the other formulations. Specifically, the percentages of brain uptake were higher for the IN solution and ME than for the IV solution.	Sayad *et al.* (2019) 68
2.1.3	[^123^I]	SPECT/Gamma	48 h	Biodistribution study.	To evaluate the efficiency of IN administration of a radiolabeled anti-Aβ peptide for targeting the brain, particularly for potential treatment of Alzheimer's disease.	The study found that the peak brain uptake of the peptide was at 0.5 h post-treatment, indicating rapid absorption. The uptake rate then gradually decreased over the next 48 h.	Lo *et al.* (2022) 69
2.1.3	[^131^I]	SPECT/Gamma	-	Biodistribution study.	To develop [^131^I]trazodone hydrochloride as a new radiopharmaceutical for brain imaging, evaluating its effectiveness when delivered through IN and IV routes.	The study concluded that [^131^I]trazodone, especially in the IN administration ME formulation, showed higher brain uptake than IN and IV solutions, surpassing the uptake levels of commonly used radiopharmaceuticals.	Motaleb *et al.* (2017) 70
2.1.3	[^131^I]	SPECT/Gamma	24 h	Biodistribution study.	To develop a novel radiopharmaceutical probe, ^131^I-rolapitant, using spanlastic nanovesicles for efficient brain imaging through IN administration, targeting neurokinin-1 receptors.	The outcome demonstrated significantly enhanced brain uptake and bioavailability of the probe, offering a promising method for non-invasively diagnosing and monitoring brain-related disorders.	Fayez *et al.* (2023) 71
2.1.3	[^131^I]	SPECT/Gamma	2 h	Biodistribution study.	To develop a SPECT imaging tracer targeting the endothelin 1 receptor A in the brain by radiolabeling ferulic acid with [^131^I] and formulating it into nanosized polymeric micelles for IN delivery.	The results demonstrated that these IN [^131^I]-ferulic acid polymeric micelles achieved significantly higher brain uptake compared to both IN and IV [^131^I]-ferulic acid solutions. This indicates effective brain targeting and suggests their potential as a promising imaging tracer for endothelin 1 receptor A in the brain.	Fayez *et al.* (2024) 72
2.2.1	[^18^F]	PET	30 min	Pharmacokinetic study.	To assess how hydroxypropyl cellulose viscosity and volume affect intranasal delivery of ^18^F-FDG in rats using PET imaging.	The results showed that higher viscosity improves absorption without changing membrane permeability and highlighted the significance of nasal cavity regional permeability differences for effective drug delivery.	Shingaki *et al.* (2016) 83
2.2.1	[^18^F]	PET	60 min	No validation	To test if IN administration is a feasible method for delivering radiotracers for brain PET imaging in rodents, comparing it to IV injection.	The IN-administration method showed limited effectiveness, with radiotracers mostly staying in the olfactory bulb and not spreading to other brain areas. Additionally, this method poses potential high radiation risks for human use, making it unsuitable for brain PET imaging.	Singh *et al.* (2018) 86
2.2.1	[^18^F]	PET	60 min	No validation	To use imaging to measure the transfer of materials from nose-to-brain using FLT and FDG.	Different transfer and retention patterns of FLT and FDG in the brain and olfactory bulb were successfully identified depending on the administration method (IN administration vs. tail-vein).	Ponto *et al.* (2017) 88
2.2.1	[^18^F]	PET	40 min	*Ex vivo* analysis.	To investigate how nucleoside transporters affect the movement of FLT from the nose-to-brain in rats using PET/CT.	FLT uptake concentrations were higher in the olfactory bulb than in other brain areas after IN administration. The presence of an ENT1 inhibitor affected the distribution of FLT. The results suggest that nucleoside transporters, especially ENT1, significantly influence the transport of substances from the nose-to-brain.	Ponto *et al.* (2017) 89
2.2.1	[^18^F]	PET	105 min	No validation.	To develop and test a method that non-invasively measures how the brain clears waste via the glymphatic system, using specific imaging tracers and PET/MRI scanning.	They discovered that delivering these imaging tracers, IN, was more effective for studying brain waste clearance than through the IV. Additionally, [^18^F]FDS was superior for these studies as it didn't show any unnecessary accumulation in the skull, enabling a more comprehensive and sensitive analysis of the entire brain.	Smith *et al.* (2021) 93
2.2.1	[^18^F]	PET	90 min	Biodistribution study.	To create a new imaging tool for oxytocin receptors in the brain, this tool was used to show that oxytocin can be directly transported from nose-to-brain when administered nasally.	[^18^F]-based radiotracers showed higher uptake in the olfactory bulb after IN administration of oxytocin; they did not progress to deeper brain regions within 90 min. Additionally, the uptake patterns after IN administration were similar to IV injection. Thus, the results are inconclusive about the effectiveness of using ^18^F-based radiotracers to specifically monitor the nose-to-brain delivery pathway.	Beard *et al.* (2018) 94
2.2.1	[^18^F]	PET	120 min	No validation.	To evaluate the effectiveness of different nose-to-brain delivery methods for insulin, specifically using [^18^F]-insulin, and to track its uptake and distribution in the brain using PET imaging in non-human primates.	The study found that liquid instillation of [^18^F]-insulin via catheter was the most effective method for both delivery to the subject and subsequent brain uptake. The [^18^F]-insulin was quickly transferred to key brain regions involved in emotional and memory processing. The method proved effective in tracking insulin delivery and is considered broadly applicable for evaluating other therapeutic agents, potentially advancing clinical evaluation in humans for Alzheimer's disease treatment.	Smith *et al.* (2024) 96
2.2.2	[^11^C]	PET	1.5 h	Pharmacokinetic study.	To investigate the distribution and absorption of IN administration [^11^C]zolmitriptan to healthy volunteers, using PET scanning.	Almost 100% of the dose was detected in the nasopharynx, which decreased over time. Some radioactivity was observed in the upper abdomen, very minimal in the lungs, and detection in the brain indicated central penetration of zolmitriptan. The plasma levels of zolmitriptan peaked 15 min post-dose, suggesting initial absorption across the nasal mucosa contributed to its early systemic availability.	Yates *et al.* (2005) 100
2.2.2	[^11^C]	PET	90 min	No validation.	To use PET to assess the uptake and distribution of zolmitriptan, into the CNS by IN administration in humans.	The drug was rapidly uptaken into the brain, with significant concentrations observed in all the studied brain regions. Notably, the concentration levels in the CNS were found to be compatible with a central mode of action, implying that zolmitriptan does affect the brain directly. This confirmed that zolmitriptan can enter the brain tissue in humans.	Wall *et al.* (2005) 103
2.2.2	[^11^C]	PET	90 min	*Ex vivo* imaging.	To understand the pharmacokinetic mechanism of IN administration of Orexin A, a potential antinarcoleptic drug. They used [^11^C] radioactive methylation to assess its brain uptake after IN administration in both rodents and nonhuman primates.	They found that brain exposure to orexin A is poor after IN administration. However, *ex vivo* analysis using [^125^I] showed that IN administration of Orexin A gives similar brain uptake as IV injection across most brain regions. There was potentially increased brain uptake in the olfactory bulbs.	Van de Bittner *et al.* (2018) 104
2.2.2	[^11^C]	PET	-	Autoradiography study.	To understand how oxytocin reaches the brain when given through different routes and to explore its effect on addiction-related processes.	Administering oxytocin IN resulted in significant amounts in key brain areas.	Lee (2022) 201
2.2.1	[^3^H]	Gamma	60 min	Pharmacokinetic study.	To evaluate the effectiveness of delivering [^3^H]FLT to brain tissue for imaging glioblastoma using IN administration compared to IV injection in a rat model.	IN administration of FLT showed increased efficacy in delivering the drug to all brain regions compared to IV injection. Particularly, the drug penetrated more efficiently to the olfactory bulb, spinal cord, and hippocampus with IN administration.	Carlisle *et al.* (2019) 91
2.2.3	[^89^Zr]	PET	-	Immunocytochemistry study.	To understand how therapeutic stem cells (NSCs and MSCs) delivered IN administration towards brain tumours and to enhance this movement for optimal treatment strategies.	They discovered that MSCs and NSCs have different migration paths in the brain. They identified the epidermal growth factor as a potential factor influencing this migration. They successfully modified NSCs to improve their movement towards the epidermal growth factor, potentially enhancing their therapeutic effects on brain tumours.	Yu *et al.* (2016) 105
2.2.3	[^89^Zr]	PET	4 h	Droplet digital polymerase chain reaction and immunohistochemistry study.	To develop a sensitive and reliable method for tracking the migration of hNSCs into the brain via IN in a preclinical setting, using droplet digital polymerase chain reaction, after traditional methods like BLI imaging and PET/CT proved insufficiently sensitive.	The study successfully utilised droplet digital polymerase chain reaction to effectively track the migration of hNSCs into the brain, confirming their presence and supporting their potential use in treating Parkinson's disease in an animal model. Traditional methods like BLI imaging and PET/CT were found to be insufficiently sensitive for this purpose.	Wang *et al.* (2020) 106
2.2.3	[^89^Zr]	PET	2 h	*Ex vivo* autoradiography.	To develop a protocol for tracking the delivery of NPs from the nose-to-brain in rats, using PET/CT imaging. They aim to compare IN administration to IV injection and evaluate its potential for improved brain targeting.	The study found that IN administration of LPNPs led to increased brain activity compared to IV injections, and they developed a protocol for better targeting the olfactory epithelium.	Veronesi *et al.* (2021) 107
2.2.4	[^68^Ga]	PET	12 weeks	Autoradiography study.	To evaluate the *in vitro* serum stability and autoradiography of [^68^Ga]-NOTA-insulin in rodent and non-human primate models of Alzheimer's disease pathology. Additionally, they seek to investigate the potential of [^68^Ga]-NOTA-insulin as a PET imaging probe to monitor insulin distribution and uptake *in vivo*.	The study found that [^68^Ga]-NOTA-insulin maintained over 97% stability in human serum for up to 5 h post-production. Autoradiography results showed significantly lower brain uptake of the radiotracer in transgenic mice and Alzheimer's disease vervets compared to their respective controls. The hippocampus and hypothalamus uptake was inversely correlated with Aβ density. These findings suggest that [^68^Ga]-NOTA-insulin is a specific and stable radiotracer for monitoring insulin distribution in the brain and could potentially serve as a diagnostic tool for Alzheimer's disease.	Gollapelli *et al.* (2023)^109^
2.2.5	Mn	PET	7 days	No validation.	To determine if low-dose Mn-based radiotracers can be effectively used for PET imaging to assess tissue anatomy, function, and neuronal connectivity, potentially offering a safer alternative to current MRI methods.	The study found that PET imaging with low-dose Mn-based radiotracers produced results consistent with those of higher-dose MRI methods, suggesting their potential for safely assessing anatomy, function, and connectivity in both pre-clinical and clinical settings.	Saar *et al.* (2018) 110
2.3.1	QDs	OI	24 h	Immunohistochemistry & confocal studies.	To investigate the pathways used by fluorescent QDs within the olfactory epithelium of mice following IN administration.	The outcome revealed that QDs primarily traverse the olfactory epithelium through extracellular pathways rather than neuronal routes.	Garzotto *et al.* (2010) 116
2.3.1	Cy5.5	OI	2 h	No validation.	To compare how the fluorescent imaging agent Cy5.5 is distributed in the body when IN-delivered versus through IV injection, particularly focusing on its pathway to the brain.	The study found distinct distribution patterns for Cy5.5 based on the delivery method: IN administration showed a significant presence in the stomach and olfactory bulbs, while IV injection led to a higher concentration in the liver and multiple brain regions.	Chen *et al.* (2011) 117
2.3.1 & 2.4.1	Cy5.5 & Gd	OI	24 h	Hematoxylin and eosin Staining and Nissl staining study.	To develop and evaluate a stable protein-based NPs system, CB-Gd-Cy5.5, for INDD to the brain, using Cy5.5 as a model drug for tracking distribution and metabolism.	The study successfully demonstrated that the CB-Gd-Cy5.5 system efficiently delivered drugs to the brain via the nose-to-brain pathway. This was evidenced by widespread and persistent fluorescence signals in the brain, particularly in the hippocampus, after IN administration, indicating effective targeting and potential for treating neurological disorders.	Chen *et al.* (2019) 118
2.3.1	Cy5.5 & ^111^In	OI & SPECT/Gamma	7 days & 24 h	*Ex vivo* OI analysis & inductively coupled plasma mass spectrometry & autoradiography study.	To investigate the spatiotemporal distribution of AuNRs following IN administration and assess their potential as INDD carriers for CNS applications.	The outcomes revealed a rapid and efficient delivery mechanism of AuNRs to the brain via IN administration, with specific accumulation in the olfactory bulbs.	Han *et al.* (2023) 119
2.3.1	CTG BODIPY	OI	2 h	OI analysis & scintillation analysis.	To investigate the potential of the rostral migratory stream as a pathway for delivering drugs directly to the brain via IN administration.	Their primary outcome revealed that the rostral migratory stream plays a crucial role in this process, as substances administered intranasally were effectively transported to the brain when the rostral migratory stream was intact.	Scranton *et al.* (2011) 120
2.3.1	EGFP	OI	3 h	Light microscopy & immunohistochemistry study.	To investigate whether bone marrow-derived MSCs, labelled with EGFP, could be effectively IN-delivered to treat neuropathologies in mice with striatal lesions.	The outcome of the study was that the MSC-EGFPs were undetectable in the brain from 3 h to 2 months post-administration, suggesting that they did not survive or migrate effectively when administered intranasally.	Chartoff *et al.* (2011) 122
2.3.1	FITC	OI	24 h	No validation.	To explore the feasibility of delivering insulin directly to the brain using an IN ME method, potentially offering a new treatment approach for CNS disorders.	It demonstrated that this method resulted in a significantly higher uptake of insulin in the brain compared to traditional IN administration, aqueous solutions, and IV injection.	Botner *et al.* (2012) 123
2.3.1	Alexa Fluor 647	OI	30 min	Electron microscopy.	The study focused on understanding the specific pathways through which IN insulin reaches the CNS.	They found that insulin administered via IN successfully reached the olfactory bulbs via the olfactory nerve pathway. Microscopic analysis revealed that the insulin was endocytosed within certain brain cells, indicating functional activity.	Renner *et al.* (2012) 124
2.3.1	FITC	OI	3 h	Immunohistochemistry study.	To evaluate the effectiveness of clathrin-based NPs in delivering antibodies and imaging agents into the CNS of rats.	They showed that these NPs, specifically fluorescent-tagged clathrin triskelia, could noninvasively cross the BBB and rapidly distribute throughout the brain, especially when IN-administered.	Stanojević-Vitaliano *et al.* (2016) 126
2.3.1	TR-Dex	OI	30 min	*Ex vivo* OI study & immunofluorescence & confocal microscopy.	To monitor how fluorescent tracers, specifically TR-DEXs of different sizes (3 and 10 kDa), are distributed within the brain following IN administration in rats.	They revealed that these tracers, especially the smaller 3 kDa TR-Dex, are rapidly and widely distributed within the brain through convective transport in cerebral perivascular spaces.	Lochhead *et al.* (2015) 125
2.3.1	DiR	OI	16 h	Biodistribution study & confocal laser scanning microscopy & *ex vivo* analysis.	To evaluate NEs for delivering drugs from the nose-to-brain.	The key outcome was that smaller NEs (around 100 nm) were more effective in retaining the nasal cavity and partially reaching the brain. However, the study primarily found that the drugs (cargoes) within the NEs were successfully delivered to the brain, even if the NEs themselves were not transported extensively.	Ahmad *et al.* (2016) 127
2.3.1	DiR	OI	24 h	Ultra-performance liquid chromatography-tandem mass spectrometry.	To improve the delivery and effectiveness of edaravone by using NP technology. Specifically, the researchers aimed to enhance the brain bioavailability of edaravone by formulating it into PLGA NPs and administering it intranasally.	They showed that the IN administration of these NPs led to higher and more sustained levels of edaravone in the brain than traditional IV injection.	Lu *et al.* (2023) 128
2.3.1	Rhodamine B	OI	24 h	Hematoxylin and eosin staining for histopathology study.	To enhance the antidepressant-like activity of icariin by improving its delivery to the brain using an IN nanogel-thermoresponsive hydrogel system	Successful delivery of icariin to the brain within 30 min, demonstrating significant antidepressant effects in animal models, indicating the potential for more effective depression treatment.	Xu *et al.* (2020) 129
2.3.1	Cy5.5, Cou-6, GFP	OI	6 h	Brain targeting ability & histopathology studies.	To target glioblastoma more effectively, a disulfiram-loaded NE gel, IN-administered to bypass the BBB.	Enhanced targeting and accumulation of the drug in the brain, evidenced by strong fluorescence signals. The treatment showed promising results in inhibiting glioblastoma growth and extending survival in animal models.	Qu *et al.* (2021) 130
2.3.1	IR780	OI	3 h	Biodistribution & *ex vivo* permeation studies.	To develop a co-delivery system using NPs for the targeted treatment of glioblastoma via nose-to-brain delivery.	Effective delivery of the drug combination to the brain, with significant fluorescence, was observed 30 min after IN administration. The system showed potential in inhibiting tumour growth and could be a promising approach for glioblastoma treatment.	Ferreira *et al.* (2021) 131
2.3.1	far-red	OI	-	Computational simulation.	The researchers sought to enhance INDD using transcranial magnetic stimulation and fluorescent magnetic NPs by overcoming the limitations of traditional drug delivery methods and the BBB.	They demonstrated that applying transcranial magnetic stimulation could significantly increase the delivery of fluorescent magnetic NPs to the brain, achieving up to a two-fold increase compared to passive delivery methods. Additionally, they found that smaller fluorescent magnetic NPs were more efficiently delivered, and the technique was safe, with no significant damage to major organs.	Ye *et al.* (2023) 132
2.3.1	PS-YG	OI	24 h	Immunofluorescence analysis & quantitative reverse transcription polymerase chain reaction analysis.	To investigate the uptake, accumulation, and neurotoxic effects of polystyrene NPs in the brain, specifically following IN administration in mice.	The study revealed that polystyrene NPs can rapidly accumulate in the brain, with significant uptake observed within 3 h of administration. This accumulation led to an acute neurotoxic response, indicated by increased levels of neurotoxic and inflammatory markers.	Han *et al.* (2023) 133
2.3.1	MIL-100 & FITC	MRI & OI	24 h	*In vivo* FLI & MRI.	To develop and evaluate an IN-delivery using MIL-100 combined with domperidone, aiming to improve the delivery of therapeutic agents to the brain. The system intended to leverage near-infrared irradiation and magnetic fields to enhance brain permeability and achieve targeted drug release.	The study demonstrated that the IN-delivery effectively delivered the therapeutic agents to the brain, primarily accumulating in the olfactory bulbs and subsequently distributing to the prefrontal cortex and hippocampus within the first 24 h. MRI and *in vivo* FLI confirmed significant brain uptake and behavioural experiments showed notable improvements in depression, anxiety, and cognitive behaviours in mice.	Hu et al (2024) 134
2.3.1	Doxorubicin	OI	24 h	No validation.	To develop and evaluate an intranasal chitosan hydrogel for delivering morphine to achieve fast and prolonged analgesic effects.	FLI demonstrated that the IN chitosan hydrogel effectively transported the drug to the brain, with 74% absorption in the olfactory bulbs compared to 15% in control groups. The imaging confirmed prolonged retention and sustained presence of the hydrogel in the nasal cavity and brain, contributing to the extended analgesic effects observed.	Kamali *et al.* (2024) 135
2.3.1	Cou-6 & Rhodamine B	OI	24 h	*Ex vivo* biodistribution study.	To develop and evaluate multifunctional NPs that target mitochondria and respond to reactive oxygen species for treating ischemic stroke.	The study successfully demonstrated, using FLI, that the multifunctional NPs could rapidly accumulate in the brain's ischemic regions, particularly the ischemic penumbra. The NPs showed sustained release and prolonged therapeutic effects, effectively alleviating oxidative stress, reducing inflammation, repairing mitochondrial function, and decreasing apoptosis.	Zhang *et al.* (2023) 136
2.3.1	FITC	OI	6 h	Biodistribution study.	To enhance the delivery of NAC from nose-to-brain, the researchers aimed to create multifunctional hydrogels by incorporating dopamine polydopamine into hyaluronic acid silk fibroin hydrogels.	FLI showed a 9-fold increase in NAC delivery to the brain within 2 h using multifunctional hydrogels with near-infrared irradiation compared to NAC solution alone. Near-infrared irradiation effectively opened tight junctions in nasal epithelial cells, facilitating NAC delivery. NAC release from multifunctional hydrogels was sustained over 6 h, with increased release rates under near-infrared irradiation.	Chung *et al.* (2023) 137
2.3.1	Cy3 & Cy7	OI	6 h	Near-infrared FLI & immunofluorescence imaging.	To investigate the distribution and delivery efficiency of MSC-derived small extracellular vesicles administered intranasally for CNS therapy.	The researchers found that IN MSC-derived small extracellular vesicles rapidly and widely distributed throughout the brain, particularly in the subcortex, and were taken up by neurons, astrocytes, and microglia.	Shen *et al.* (2023) 138
2.3.1	Rhodamine B	OI	5 h	Drug distribution & pharmacodynamics studies.	To evaluate the effectiveness of rivastigmine nasal spray for enhancing brain delivery in Alzheimer's disease treatment.	The optimised nasal spray formulation provided rapid and efficient drug uptake into the brain, with fluorescence imaging showing strong signals in key brain regions within 15 min, peaking at 60 min. The study confirmed that the drug was effectively transported via olfactory and trigeminal pathways, highlighting the importance of optimising formulation viscosity and deposition for improved brain delivery.	Guo *et al.* (2024) 139
2.3.1	Cy5.5	OI	24 h	Pharmacokinetic study.	To improve the efficiency of PHT delivery to the brain for treating epileptic seizures using the IN administration of BSA-LDHs-PHT.	The study successfully demonstrated that BSA-LDHs-PHT significantly improved brain targeting and retention of PHT. FLI showed that the NPS provided over twice the brain uptake compared to the control at 15 min post-administration, maintaining high levels for up to 24 h. The highest brain concentration of phenytoin was 404.3 ng/mL at 4 h, remaining high for up to 8 h.	Zhang *et al.* (2024) 140
2.3.1	Cy5, FITC	OI	48 h	Western blot & immunohistochemistry studies.	To investigate the targeted delivery and therapeutic efficacy of anti-apoptotic siRNA, complexed with FBP and 9R, in reducing neuronal cell death in brain ischemia via IN administration.	The IN administration of FBP9R/siRNA complexes successfully targeted the ischemic brain region, significantly localising within 12 h and remaining retained for up to 48 h. This targeted delivery effectively reduced apoptosis in the ischemic region, demonstrating promising therapeutic potential for treating brain ischemia.	Chung *et al.* (2024) 141
2.3.1	DiR	OI	24 h	*In vivo* & *ex vivo* biodistribution & immunohistochemistry studies.	To investigate the therapeutic potential of miRNAs carried by small extracellular vesicles derived from Wharton's jelly MSCs for treating premature white matter injury in a preclinical rat model.	The study found that intranasally administered Wharton's jelly MSCs small extracellular vesicles rapidly reached the brain, where they remained detectable for at least 24 h, primarily accumulating in the corpus callosum and external capsule.	Tscherrig *et al.* (2024) 142
2.3.2	FLUC-EGFP, Cy5.5	OI	72 h	Cell viability & immunoblot & MRI, CT and* ex vivo* studies.	To develop and test a novel method for delivering therapeutic microRNAs directly to glioblastoma cells in the brain using IN-administered polyGIONs.	The researchers demonstrated that the IN administration of polyGIONs loaded with therapeutic microRNAs (miR-100 and antimiR-21) resulted in efficient targeting and accumulation of these agents in glioblastoma cells in the brain.	Sukumar *et al.* (2019) 143
2.4.1	Gd	MRI	1 h	No validation.	Using MRI to validate and assess the IN migration of the positively charged CSA-NE to and within the CNS.	MRI revealed a distinctive and extensive dispersal in most brain areas post-IN administration of NE-encapsulated Gd ions, as manifested by the reduction in T1 values compared with the Gd-DTPA solution.	Yadav *et al.* (2018) 148
2.4.2	Mn	MRI	72 h	No validation.	To determine if Mn^2+^ could be used as a T1 contrast agent for MRI to trace neuronal pathways in live mice.	The study's outcome demonstrated that Mn^2+^ could effectively highlight specific neuronal pathways in the olfactory and visual systems of mice. Mn^2+^ has transported anterogradely and provided clear MRI signal enhancements in the olfactory bulb, primary olfactory cortex, pituitary gland, optic nerve, and superior colliculus. This indicated that Mn^2+^ is a viable agent for *in vivo* neuronal tract tracing using MRI, providing detailed visualisation of neuronal connections and pathways.	Pautler *et al.* (1998) 149
2.4.2	Mn	MRI	1.5 h	No validation.	To use Mn-enhanced MRI to trace neuronal activation from the olfactory epithelium to the olfactory bulb in mice exposed to specific odours.	The study successfully demonstrated that Mn-enhanced MRI effectively traced Mn^2+^ transport from the olfactory epithelium to the olfactory bulb. Different odours caused distinct, localised enhancements in the olfactory bulb, indicating odour-specific neuronal activation. Mn-enhanced MRI provided detailed, high-resolution maps of activated brain regions, with Mn^2+^ remaining in these regions long enough for thorough imaging. The study confirmed that Mn^2+^ entered neurons through calcium channels and was transported anterogradely, providing mechanistic insights into the pathways of odour-induced neuronal activation.	Pautler *et al.* (2002) 150
2.4.2	Mn	MRI	-	Inductively coupled plasma mass spectrometry.	To determine if Mn, after IN administration, can bypass the BBB and further transfer from the olfactory bulb to higher brain regions.	The study found that visual stimulation allows Mn to travel from the olfactory bulb to the visual cortex. Rats with intact olfactory bulbs showed significantly higher Mn content and signal intensities in the visual cortex compared to rats without olfactory bulbs. This indicates that Mn can migrate from the olfactory bulb to the visual cortex following IN administration.	Fa *et al.* (2010) 151
2.4.2	Mn	MRI	24 h	Transmission electron microscopy & inductively coupled plasma atomic emission spectroscopy.	To use the Mn_3_O_4_-NPs & Fe_3_O_4_-NPs as MRI tracers to study and map the transport of NPs from nose-to-brain.	They found that Mn_3_O_4_-NPs can be transported from the nose-to-brain, with their movement influenced by neuronal activity, olfactory stimuli, and conditions like aging and Parkinson's disease. This offers potential for new diagnostic methods and drug delivery routes.	Romashchenko *et al.* (2023) 152
2.4.2	Mn	MRI	24 h	PET & OI studies.	To develop and evaluate a novel INDD system, which targets the olfactory region in cynomolgus monkeys for efficient drug transport directly to the brain.	They successfully demonstrated that the nose-to-brain system, comprising a mucoadhesive powder formulation and a dedicated nasal device, significantly enhanced the delivery and retention of drugs in the olfactory region compared to other nasal delivery systems. This targeted approach resulted in greater drug accumulation in the brain, particularly in the olfactory bulb, suggesting a more efficient method for delivering drugs across the BBB, particularly for compounds with poor permeability.	Sasaki *et al.* (2023) 153
2.4.3	USPIO	MRI	24 h	Transmission electron microscopy.	To develop a method of loading USPIO-ADEVs and trace them *in vivo* MRI.	The MRI results revealed that USPIO-ADEVs accumulate in the brain, liver, and kidneys within a 24-h timeframe.	Kutchy *et al.* (2022) 154
2.4.3	SPIO	MRI	120 h	Histology, fluorescent microscopy, and confocal microscopy studies.	Using MRI to demonstrate the potential of using methimazole to extend the residence time NSCs.	They observed localised hypointensity and an increase in T2* in all subjects treated with methimazole, indicating the migration of SPIO.	Spencer *et al.* (2019) 155
2.4.3	MPIO & ^111^In	MRI & SPECT/Gamma	2 days	BLI & Prussian blue staining.	To investigate the feasibility and effectiveness of IN administration of MSCs to deliver these cells to the brain.	The study provided promising evidence that MSCs could be delivered to the brain via the nasal route and that this method could be visualised and tracked using advanced imaging techniques. These findings suggest the potential for IN administration of MSCs to serve as a therapeutic delivery method for brain tumours, with the possibility of enhancing treatment efficacy, particularly in irradiated environments.	Balyasnikova et al (2014) 156
2.4.3	SPIO	MRI	14 days	Prussian blue staining.	To conduct longitudinal *in vivo* tracking of MSCs to advance stem cell therapies for treating brain injuries.	MRI images demonstrated a reduction in signal in the regions surrounding the injury site for the group that received the SPIO-MSCs. Indicative of the presence of SPIO particles at the injury site. These findings suggest the feasibility of real-time MRI tracking to monitor the distribution of SPIO through the nose-to-brain pathway.	Shahror *et al.* (2019) 162
2.4.3	SPIO	MRI	5 days	Prussian Blue staining & immunofluorescence studies.	To explore the nose-to-brain pathway as a potential method to deliver anti-cancer treatments to diffuse intrinsic pontine glioma, by using SPIO-MSCs to bypass the BBB.	They noticed that the MSCs were transported via the trigeminal pathway to the xenografts in the brainstem, which was evidenced by a localised hypointensity in the brainstem.	Chastkofsky *et al.* (2017) 161
2.4.3	SPIO	MRI	2 days	Prussian blue staining. & transmission electron microscopy & inductively coupled plasma mass spectrometry studies.	To employ the IN of Alg-SPIO olfactory ecto-MSCs, as an intervention for Parkinson's disease.	The T2 MRI showed hypointense signals in the Alg-SPIOs-OE-MSCs, which became increasingly pronounced with the rise of SPIO concentration, indicating that the MRI effectively detected these labelled cells.	Simorgh *et al.* (2021) 163
2.4.3	Fe_3_O_4_ & Cy5.5	MRI	2 h	*Ex vivo* study & fluorescence microscopy & hematoxylin and eosin staining.	To understand the pathways and mechanisms of IN NPs delivery to the CNS for potential improvements in CNS disease treatments.	They identified the trigeminal and olfactory nerves as major pathways for CNS delivery. The study offered significant insights into efficient drug delivery mechanisms to the CNS, which could enhance treatment strategies for CNS diseases.	Kou *et al.* (2023) 164
2.4.3	Iohexol-liposomes	MRI	1.5 h	Histology study.	CEST MRI can be used to track and monitor liposome-based drug delivery to the brain non-invasively via nose-to-brain.	They demonstrated the ability to noninvasively detect nose-to-brain delivery of liposomes using CEST MRI. Specific contrasts were observed in the brain after IN administration, particularly with 10% PEG Iohexol-liposomes, indicating that this imaging technique can effectively track drug delivery to the brain.	Law *et al.* (2023) 165
2.4.3	SPIO	MRI	72 h	Superconducting quantum interference device & fluorescence microscopy studies.	To evaluate the effectiveness of IN administration in delivering magnetic NPs to the brain, specifically targeting Aβ plaques for magnetic particle imaging, thereby bypassing the BBB.	The study demonstrated that approximately 0.3-0.6% ID successfully migrated to the brain, maintained their magnetic properties, and selectively accumulated at Aβ plaques. This indicates the potential of nasal administration as a non-invasive method for brain imaging and targeted delivery of therapeutic agents.	Seino *et al.* (2024) 166
2.5.1	Microbubble	FUS	1 h	FLI	To enhance the delivery of IN drugs to specific regions within the brain rather than allowing widespread distribution	FUS+IN administration led to 8-fold more efficient delivery to the specified region than the delivery achieved through IN administration alone.	Chen *et al.* (2014) 171
2.5.1	Microbubble	FUS	1 h	FLI	To demonstrate the underlying mechanism by which FUS enhances nose-to-brain delivery and to evaluate the feasibility of utilising this approach to deliver therapeutic molecules.	They discovered that applying FUS sonication as a pre-treatment, followed by IN administration, didn't significantly alter the delivery results. This research indicates that FUS could potentially enhance the delivery of drugs from nose-to-brain, potentially through active pumping transport.	Chen *et al.* (2016) 172
2.5.2	Microbubble & [^64^Cu]	FUS	24 h	PET & gamma counting & autoradiography & FLI studies.	To provide and assess the biodistribution profile of IN-administered AuNCs and assess the feasibility and short-term safety of FUS+IN administration for the delivery of the NP to the brainstem.	The study demonstrated that IN administration of ^64^Cu-alloyed AuNCs resulted in significantly lower accumulation in blood and major organs than IV injections, indicating minimal systemic exposure. Additionally, the combination of FUS+IN significantly enhanced the local delivery of AuNCs to the FUS-targeted brain region, with increased accumulation observed in the brainstem. Importantly, no histological damage was detected in the nose, trigeminal nerve, and brain after FUS+IN treatment, suggesting that this technique is safe for noninvasive, targeted brain delivery.	Ye *et al.* (2018) 173
2.5.2	Microbubble	FUS	1 h	*Ex vivo* FLI & immunofluorescence staining & enzyme-linked immunosorbent studies.	To assess the ability of FUS for INDD to have more localise area for aPD-L1 antibody	When compared to IN alone, the enhancement of aPD-L1 uptake at the brainstem was observed to be approximately 4.03-fold and 3.74-fold higher in non-tumour and glioma mice, respectively.	Ye *et al.* (2021) 176
2.5.2	Microbubble	FUS	1 month	Immunohistological staining & Droplet digital polymerase chain reaction.	To assess and determine the efficiency and viability of using the FUS+IN administration method to deliver AAV5 to the brain.	FUS+IN administration could safely and effectively deliver AAV5-EGFP to targeted regions of the brain, including both superficial and deep areas. FUS+IN administration achieved comparable results to the direct intracranial administration. FUS+IN administration showed dramatically higher efficiency, specifically 414.9 times more than FUS-BBB disruption and 2073.7 times more than IN.	Ye *et al.* (2022) 177
2.6.1	AuNP	CT	24 h	Inductively Coupled Plasma spectrometer.	Developing an imaging technique to trace the pathway of MSC-derived exosomes that were labelled with glucose-coated AuNPs.	The study showed that exosomes started accumulating in the mouse model's brain within an hour of being administered. After 3 h, there was a notable concentration of these exosomes at the site of the stroke, which was observed to last for as long as 24 h.	Betzer *et al.* (2018) 179
2.6.1	AuNP	CT	-	*Ex vivo* using fluorescence microscopy & histopathological studies.	*In vivo* evaluation and bioaccumulation investigation of brain-targeted resveratrol delivery via nose-to-brain lipid vesicles labelled with AuNPs.	They effectively showed that AuNPs built up in the brains of all the rats treated with CT scans. From the CT images, they were able to distinctly pinpoint bright areas in the brain, indicative of AuNP accumulation.	Salem *et al.* (2019) 180
2.6.1	AuNP	CT	-	Confocal laser scanning microscopy.	They aimed to use sol-gel for effective nose-to-brain delivery of citicoline, which will improve the treatment and management of epilepsy disorders. Moreover, they optimised a formulation labelled with AuNPs and subjected it to CT to assess brain uptake and cellular translocation following IN administration of citicoline.	The CT images revealed significant attenuation of the X-ray beam across various brain regions, which implies the accumulation of AuNPs. The bright areas seen on the CT scans suggest that IN administration of AuNP-labelled citicoline-loaded niosomes bypassed the BBB and effectively reached different parts of the brain.	Bekhet *et al.* (2022) 181

Abbreviations: [^11^C]: carbon-11; [^18^F]: fluorine-18; [^3^H]: tritium; [^68^Ga]: gallium-68 [Mn]: manganese; [^64^Cu]: copper-64; [^86^Rb]: rubidium-86; [^89^Zr]: zirconium-89; [^99m^Tc]: technetium-99m; [^111^In]: Indium-111; [^123^I]: Iodine-123; [^125^I]: Iodine-125; [^131^I]: Iodine-131; [^201^Tl]: thallium-201; AAV5: adeno-associated virus serotype 5; ADEVs: astrocyte-derived extracellular vesicles; Aβ: amyloid β; Alg: Alginate; AuNCs: gold nanoclusters; AuNRs: gold nanorods; BBB: blood-brain barrier; BLI: bioluminescence imaging; BODIPY: boron-dipyrromethene; BSA: bovine serum albumin; CB: cholera toxin B subunit; CEST: chemical exchange saturation transfer; CNS: central nervous system; CSF: cerebrospinal fluid; CSA: cyclosporine A; CT: computed tomography; CTG: CellTracker Green; Cy: cyanine dye; Ct: cholera toxin; Cou-6: coumarin-6; DIPG: diffuse intrinsic pontine glioma; DTPA: diethylenetriamine pentaacetate; Dex: dextran; DiR: 1,1'-dioctadecyl-3,3,3',3'-tetramethylindotricarbocyanine iodide; EGFP: enhanced green fluorescent protein; ENT1: equilibrative nucleoside transporter 1; FBP: Fas-signaling blocking peptides; FDG: fluorodeoxyglucose; FDS: 2-deoxy-2-fluorosorbitol; FITC: fluorescein isothiocyanate; FLI: fluorescence imaging; FLUC: firefly luciferase; FLT: fluorothymidine; Fe3O4: iron oxide; FUS: focused ultrasound; AuNP: gold nanoparticle; Gd^+3^: gadolinium ion; GFP: green fluorescent protein; GM1: monosialoganglioside; IGF-1: Insulin-like growth factor-1; IFN-β1b: interferon-β1b; INDD: intranasal drug delivery; IN: intranasal; IR780: near-infrared dye 780; IV: intravenous; LDH: layered double hydroxide; LPNPs: lipid-polymer nanoparticles; MEs: microemulsions; MRI: magnetic resonance imaging; MIL-100: a type of metal-organic framework; MSCs: mesenchymal stem cells; MTX: methotrexate; Mn^2+^: manganese ion; miRNA: micro RNA; MPIOs: micron-size paramagnetic iron oxide; NOTA: ((1,4,7-triazanonane-1,4,7-triyl) triacetic acid); NE: nanoemulsion; NAC: N-acetyl-L-cysteine; NPs: nanoparticles; NSCs: neural stem cells; OI: optical imaging; PET: positron emission tomography; PHT: phenytoin; PLGA: polylactic-co-glycolic acid; PS-YG: polystyrene nanoparticles labelled with yellow green; QDs: quantum dots; SPIONs: superparamagnetic iron oxide nanoparticles; SV2A: synaptic vesicle glycoprotein 2A; siRNA: small interfering RNA; SPECT: single photon emission computed tomography; TR-Dex: Texas Red dextran; USPIO: ultrasmall superparamagnetic iron oxide; VEGF: vascular endothelial growth factor; WGA: wheat germ agglutinin; bFGF: basic fibroblast growth factor; hNSCs: human neural stem cells; polyGIONs: polyfunctional gold-iron oxide NPs.

## Abbreviations

[^11^C]: carbon-11
[^18^F]: fluorine-18
2D: two dimensional
3D: three dimensional
[^3^H]: tritium
5-HT_1B/1D_: subtypes of serotonin receptors
[^68^Ga]: gallium-68
[^51^Mn]: manganese-51
[^52^Mn]: manganese-52
[^54^Mn]: manganese-54
^54^MnCl_2_: manganese-54 dichloride
[^64^Cu]: copper-64
[^86^Rb]: rubidium-86
[^89^Zr]: zirconium-89
[^99m^Tc]: technetium-99m
[^111^In]: Indium-111
[^123^I]: Iodine-123
[^125^I]: Iodine-125
[^131^I]: Iodine-131
[^201^Tl]: thallium-201
^201^TlCl: thallium-201 chloride
AAV5: adeno-associated virus serotype 5
ADEVs: astrocyte-derived extracellular vesicles
AlF-NODA-dLVT: aluminum fluoride-NODA-oxytocin derivative
Aβ: amyloid β
Alg: alginate
AuNCs: gold nanoclusters
AuNRs: gold nanorods
AUC: area under the curve
BBB: blood-brain barrier
BLI: bioluminescence imaging
BNDF: brain-derived neurotrophic factor
BODIPY: boron-dipyrromethene
BSA: bovine serum albumin
CB: cholera toxin B subunit
CEST: chemical exchange saturation transfer
CHC: hydroxycinnamic acid
CNS: central nervous system
CNT: concentrative nucleoside transporters
CSF: cerebrospinal fluid
CSA: cyclosporine A
CF: carbonised framework
CT: computed tomography
CTG: CellTracker Green
Cy: cyanine dye
CdSe-ZnS: cadmium selenide-zinc sulfide
Ct: cholera toxin
Cou-6: coumarin-6
C_max_: maximum concentration
DIPG: diffuse intrinsic pontine glioma
DSF-INEG: disulfiram-loaded ion-sensitive nanoemulsion gel
DTP%: direct transport percentage
DTPA: diethylenetriamine pentaacetate
DTE%: drug targeting efficiency percentage
Dex: dextran
DiR: 1,1'-dioctadecyl-3,3,3',3'-tetramethylindotricarbocyanine iodide
EPO: erythropoietin
EGFP: enhanced green fluorescent protein
ENT1: equilibrative nucleoside transporter 1
FA-TEPA: folic acid tetraethylenepentamine conjugate
FDG: fluorodeoxyglucose
FDS: 2-deoxy-2-fluorosorbitol
FBP: Fas-signaling blocking peptides
FcM: Ferucarbotran Magnetic
FITC: fluorescein isothiocyanate
FLI: fluorescence imaging
FLUC: firefly luciferase
FLT: fluorothymidine
Fe: iron
Fe3O4: iron oxide
FUS: focused ultrasound
AuNP: gold nanoparticle
Gd^+3^: gadolinium ion
GFP: green fluorescent protein
GM1: monosialoganglioside
IGF-1: Insulin-like growth factor-1
IFN-β1b: interferon-β1b
ID/g: injected dose per gram
INDD: intranasal drug delivery
IN: intranasal
IR780: near-infrared dye 780
IV: intravenous
LDH: layered double hydroxide
LPNPs: lipid-polymer nanoparticles
MEs: microemulsions
MRI: magnetic resonance imaging
MIL-100: a type of metal-organic framework
MSCs: mesenchymal stem cells
MTX: methotrexate
Mn^2+^: manganese ion
miRNA: micro RNA
MPIOs: micron-size paramagnetic iron oxide
NOTA: ((1,4,7-triazanonane-1,4,7-triyl)triacetic acid)
NBMPR: nitrobenzylmercaptopurine riboside
NE: nanoemulsion
NMDA: N-methyl-D-aspartate
NAC: N-acetyl-L-cysteine
NPs: nanoparticles
NSCs: neural stem cells
OI: optical imaging
PET: positron emission tomography
PHT: phenytoin
PLA-PEG-DSPE: polylactic acid -polyethylene glycol-distearoylphosphatidylethanolamine
PLGA: polylactic-co-glycolic acid
PRISMA-S: Preferred Reporting Items for Systematic Reviews and Meta-Analyses extension for Scoping Review
PS-YG: polystyrene nanoparticles labelled with yellow green
QDs: quantum dots
R8-YAβ(25-35)-PEI: polyarginine-amyloid beta peptide-polyethyleneimine
SPIONs: superparamagnetic iron oxide nanoparticles
SV2A: synaptic vesicle glycoprotein 2A
siRNA: small interfering RNA
SUV: standard uptake value
SPECT: single photon emission computed tomography
TACs: time-activity curves
T_max_: time to reach maximum
TR: Texas Red
TT: tetanus toxoid
USPIO: ultrasmall superparamagnetic iron oxide
VEGF: vascular endothelial growth factor
WGA: wheat germ agglutinin
bFGF: basic fibroblast growth factor
cpm: counts per minute
hNSCs: human neural stem cells
polyGIONs: polyfunctional gold-iron oxide NPs
